# Classification of Common Food Lipid Sources Regarding Healthiness Using Advanced Lipidomics: A Four-Arm Crossover Study

**DOI:** 10.3390/ijms24054941

**Published:** 2023-03-03

**Authors:** Milena Monfort-Pires, Santosh Lamichhane, Cristina Alonso, Bjørg Egelandsdal, Matej Orešič, Vilde Overrein Jordahl, Oda Skjølsvold, Irantzu Pérez-Ruiz, María Encarnación Blanco, Siv Skeie, Catia Martins, Anna Haug

**Affiliations:** 1 Faculty of Chemistry, Biotechnology and Food Science, Norwegian University of Life Sciences, 1430 Ås, Norway; 2 Turku Bioscience Centre, University of Turku, 20520 Turku, Finland; 3 OWL Metabolomics, 48160 Derio, Spain; 4 School of Medical Sciences, Örebro University, 702 81 Örebro, Sweden; 5 Department of Neuromedicine and Movement Science, Norwegian University of Science and Technology (NTNU), 7034 Trondheim, Norway; 6 Centre for Health Innovation, 6509 Kristiansund, Norway; 7 Obesity Research Group, Department of Clinical and Molecular Medicine, Faculty of Medicine and Health Sciences, Norwegian University of Science and Technology (NTNU), 7034 Trondheim, Norway; 8 Centre for Obesity and Innovation (ObeCe), Clinic of Surgery, St. Olav University Hospital, 7030 Trondheim, Norway; 9 Department of Nutrition Sciences, University of Alabama at Birmingham (UAB), Alabama, AL 35294, USA; 10 Faculty of Biosciences, Norwegian University of Life Sciences, 1433 Ås, Norway

**Keywords:** animal fat, cheese, pork meat, beef meat, lipidomics, cardiovascular risk markers, liquid chromatography–mass spectrometry (LC-MS)

## Abstract

Prospective studies have failed to establish a causal relationship between animal fat intake and cardiovascular diseases in humans. Furthermore, the metabolic effects of different dietary sources remain unknown. In this four-arm crossover study, we investigated the impact of consuming cheese, beef, and pork meat on classic and new cardiovascular risk markers (obtained from lipidomics) in the context of a healthy diet. A total of 33 young healthy volunteers (23 women/10 men) were assigned to one out of four test diets in a Latin square design. Each test diet was consumed for 14 days, with a 2-week washout. Participants received a healthy diet plus Gouda- or Goutaler-type cheeses, pork, or beef meats. Before and after each diet, fasting blood samples were withdrawn. A reduction in total cholesterol and an increase in high density lipoprotein particle size were detected after all diets. Only the pork diet upregulated plasma unsaturated fatty acids and downregulated triglycerides species. Improvements in the lipoprotein profile and upregulation of circulating plasmalogen species were also observed after the pork diet. Our study suggests that, within the context of a healthy diet rich in micronutrients and fiber, the consumption of animal products, in particular pork meat, may not induce deleterious effects, and reducing the intake of animal products should not be regarded as a way of reducing cardiovascular risk in young individuals.

## 1. Introduction

Diet is one of the most important modifiable risk factors associated with obesity and non-communicable chronic diseases (NCDs) [1,2], the latter being the leading cause of mortality worldwide [1,2]. Among the dietary factors, the intake of saturated fatty acids (SFAs) has been implicated in increased inflammation, impaired insulin signaling, and increased cardiovascular disease (CVD) risk [3,4,5]. Although the deleterious metabolic effects of SFAs have been widely demonstrated in experimental models [3,4,6,7], prospective studies and meta-analyses have failed to establish a causal relation between overall SFA consumption and CVD or all-cause mortality in humans [8,9,10,11], possibly due to the synergistic effects of human dietary habits. Nevertheless, dietary guidelines have been recommending limiting the consumption of animal products containing SFA, especially red meat and regular-fat dairy products to reduce all-cause and CVD mortality risks [12,13].

Studies conducted in the last 15 years investigating the effects of meat and dairy products on the CVD risk profile have shown conflicting results [10,14,15,16,17,18]. Although some studies have found an increased risk for CVD from red-meat intake [10,14], others observed an increased risk only for processed meat intake [15], or no effect at all [18]. The source of red meat (pork, beef, or other types of meat), as well as the degree of processing (such as salting, smoking, or the inclusion of additives), could, at least in part, explain the different results across studies [10,14,15,18]. Moreover, controversial findings have also been reported for cheese consumption [16,17,19]. Some studies, but not all, associated cheese and/or dairy consumption with lower CVD risk, although the mechanisms remain elusive [17,19]. It has been hypothesized that the elevated calcium content in cheese (which may lead to higher fecal fat excretion rates), or the fermentation process, could offer protective effects on CVD outcomes [16,20,21], despite the high content of palmitic and myristic acids, which has been previously associated with inflammation and insulin resistance [3,7,20].

More recently, advanced techniques, such as lipidomics by mass spectrometry (MS) and lipoprotein subclass analysis using nuclear magnetic resonance (NMR) spectroscopy, have shed new light on the effects of dietary fats on biomarkers of health outcomes [22]. It has been reported that the consumption of SFAs, but not polyunsaturated fatty acids (PUFAs), is associated with higher plasma sphingolipids (including ceramides and sphingomyelins), which are shown to affect metabolic processes related to CVDs, promoting insulin resistance and inflammation [23,24]. Moreover, increased plasmalogens, glycerophospholipids that play a key role in biological functions and act as a potential antioxidant [25], were observed after 18 months of a healthy Nordic diet rich in fiber, fish, and berries, but not after a diet with average nutrient intake in Nordic countries [26]. This indicates the potential for MS/NMR to identify underlying metabolic pathways associated with the intake of nutrients and early CVD risk in young individuals.

Few clinical trials have investigated the effects of products that are major sources of SFAs in the context of a healthy diet [27]. In addition, it is not clear whether different animal products, with their distinct fatty acid composition, would have specific effects on metabolic outcomes, and little is known about the effects of distinct animal products on the plasma lipids species and lipoprotein subclasses. Thus, this study aims to investigate whether some of the main meat and dairy products that contribute to animal fat intake in the Norwegian diet (two cheese varieties—Gouda- and Goutaler-type cheeses, pork, and beef meat) could affect health parameters, lipoprotein subclasses (as measured by two-dimensional proton nuclear magnetic resonance spectroscopy—2D-^1^H-NMR), and lipid species (analyzed with chromatography coupled to mass spectrometry—UHPLC–MS) in healthy non-obese young individuals.

## 2. Results

### 2.1. Characteristics of the Sample

Out of 50 subjects screened, 38 subjects started the dietary intervention study. A total of 5 out of 38 participants dropped out within the first two weeks of the intervention, mainly due to the COVID-19 situation. Of 33 participants, 30 completed the intervention (8 time-points), while two males and one female had only 6 and 4 time-point data, respectively. Baseline clinical and biochemical parameters from the available participants (*n* = 33) according to sex and in the total sample are shown in Table 1. Although we had a higher number of female participants (*n* = 23) compared to males (*n* = 10), the enrolled subjects were age-matched (*p* > 0.05). In addition, the mean values of most clinical and biochemical parameters were within the normal range of being healthy.

The diet registrations for each baseline period (before each diet intervention) are summarized in Supplementary Table S1. The habitual diet remained similar throughout the study in all four diet registration periods for both sexes (Supplementary Table S1). A trend towards increased energy intake (in kilocalories—kcal) during the washout periods was detected among females (*p* = 0.07), while no differences were detected in males. When comparing the test diets with the participants’ habitual diets, we observed similar macronutrient distributions (on average: 40–43% carbohydrates, 36–39% fat, and 16–19% protein of total energy intake—TEI). Moreover, the habitual fiber consumption among participants (11.3 g/1000 kcal for males and 13.4 g/1000 kcal for females) was lower than the amount provided in the test diets (18.3 g fiber/1000 kcal for group F2 and 20 g fiber/1000 kcal for group M2).

Supplementary Table S2 depicts detailed information regarding the comparisons of macro- and micronutrients for the intervention and habitual diets in the sample. Since no differences were detected between the four washout periods, we calculated the habitual diet as the average of the four periods. Because the diet we provided had more fruits, vegetables, and fiber than the habitual diet, differences in nutrient intake were expected when comparing them. Indeed, we detected differences between the test diets and the participants’ habitual diet regarding most micronutrients analyzed, except for retinol, sodium, vitamin B6, and vitamin A, that were similar between the habitual diet and at least one of the test diets (Supplementary Table S2). Interestingly, for B12, the highest intake was observed in the habitual diet when compared to the test diets. Moreover, we compared the four test diets and observed differences among the cheese test diets and beef and pork test diets for the following nutrients: niacin (higher in beef and pork diets), phosphorus (higher in cheese diets), potassium (higher in beef and pork diets), retinol (higher in cheese diets), iron, and sodium (both higher in the beef and pork diets). In addition, the pork diet also showed a higher content of selenium and thiamin compared to others, while the beef test diet had a higher B12 content. Even though there were some differences in the nutrient content among the test diets, the major differences detected were between the habitual and test diets, indicating the significance of the quality of the diets provided during the intervention. It is worth mentioning that the proportion of nutrients and types of fats were similar between the habitual and, at least, one of the test diets. The intake of total fat in the habitual diet was similar to the test diets and the SFA intake was not different from the cheese diets, only from the beef and pork test diets. As mentioned above, the main differences between habitual and test diets were detected in the dietary fiber, MUFA, and PUFA intakes, which were higher in the test diets.

To avoid changes in physical activity levels, participants were instructed to maintain their activities throughout the study. At the end of the four test diets, a new physical activity questionnaire was filled out, and the results showed no changes in leisure-time physical activity or sitting times (Supplementary Table S3).

### 2.2. Impact of the Test Diets on Clinical Parameters

**Figure 1:**
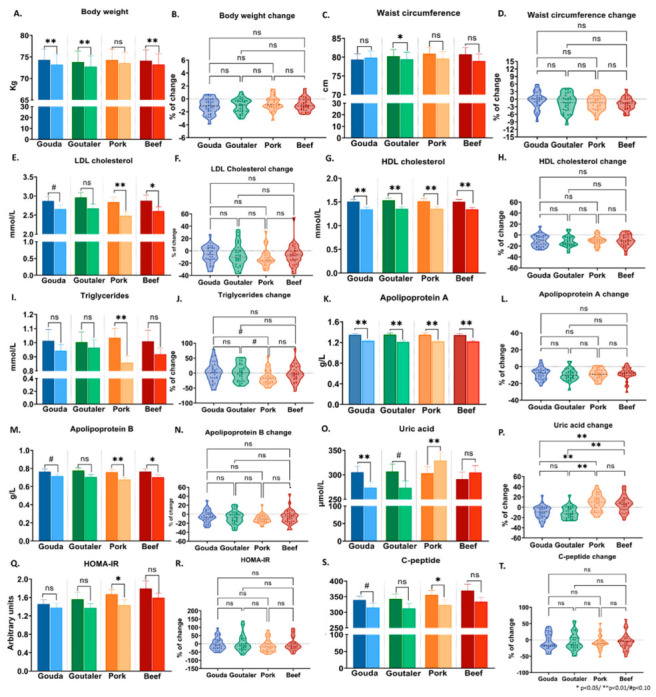
Effects of the four test diets (Gouda- and Goutaler-type cheeses, pork, and beef) on clinical markers. Before and after values for each test diet were analyzed with RM-ANOVA, while differences between test diets were investigated with mixed models for repeated measures. The figure represents the results for body weight (**A**,**B**), waist circumference (**C**,**D**), LDL cholesterol (**E**,**F**), HDL cholesterol (**G**,**H**), triglycerides (**I**,**J**), apolipoproteins A (**K**,**L**) and B (**M**,**N**), uric acid (**O**,**P**), HOMA-IR (**Q**,**R**), and C-peptide (**S**,**T**). Pre- and post-comparisons were analyzed using RM-ANOVA (for individual test diets) with body weight change and gender as covariates and period of diet as a between-subject factor (**A**,**C**,**E**,**G**,**I**,**K**,**M**,**O**,**Q**,**S**). Differences in the percent of change between diets were analyzed using mixed models for repeated measures with body weight change, period, and gender as covariates. Bonferroni’s post hoc test was used for pairwise comparisons (**B**,**D**,**F**,**H**,**J**,**L**,**N**,**P**,**R**,**T**). Symbols: n.s. = non-significant; # *p* < 0.10; * *p* < 0.05; ** *p* < 0.01.

Figure 1 highlights the effects of each test diet on the clinical parameters (Figure 1 and Supplementary Table S4). We found that all test diets promoted weight loss and reductions in body mass index (BMI), although the decline was not significant for the pork diet (Figure 1A, Supplementary Table S4). Remarkably, no significant change in weight between diets was found (Figure 1B, overall diet effect). Furthermore, a significant decrease in waist circumference (Figure 1C) was detected only after the Goutaler-type cheese diet; however, these results should be interpreted cautiously since not all waist circumference measurements were taken due to the COVID-19 contamination risk (around 15% of the measurements are missing). Interestingly, LDL cholesterol (Figure 1E) and apolipoprotein B (Figure 1M) were significantly lowered only after the pork (*p* < 0.01) and beef (*p* < 0.05) test diets. A similar trend was observed after the Gouda-type cheese test diet (Figure 1E, *p* < 0.10), but no differences were observed when comparing the four test diets (Figure 1F,N). Total cholesterol was reduced after all test diets (Supplementary Table S4), and significant reductions in both HDL cholesterol (Figure 1G) and apolipoprotein A (Figure 1K) were observed for all test diets, with there being no differences between them (Figure 1H,L). Furthermore, we observed that triglyceride levels (Figure 1I) were significantly reduced only after the pork test diet (*p* < 0.01, Figure 1I), showing a trend to decrease more than the other dietary interventions (Figure 1J). Similar findings were observed for HOMA-IR (Figure 1Q) and C-peptide (Figure 1S). Additionally, uric acid concentrations showed opposite patterns when comparing pork and beef with the cheese test diets (Figure 1O). Whereas the two cheese diets led to reduced levels, the pork and beef test diets increased uric acid concentrations (Figure 1P). Vitamin D levels (Supplementary Figure S1) increased after all test diets; however, the changes were period-dependent, and there was no difference between the diets (outlined in Supplementary Figure S1).

### 2.3. Impact of the Test Diets on Lipidomics

**Figure 2:**
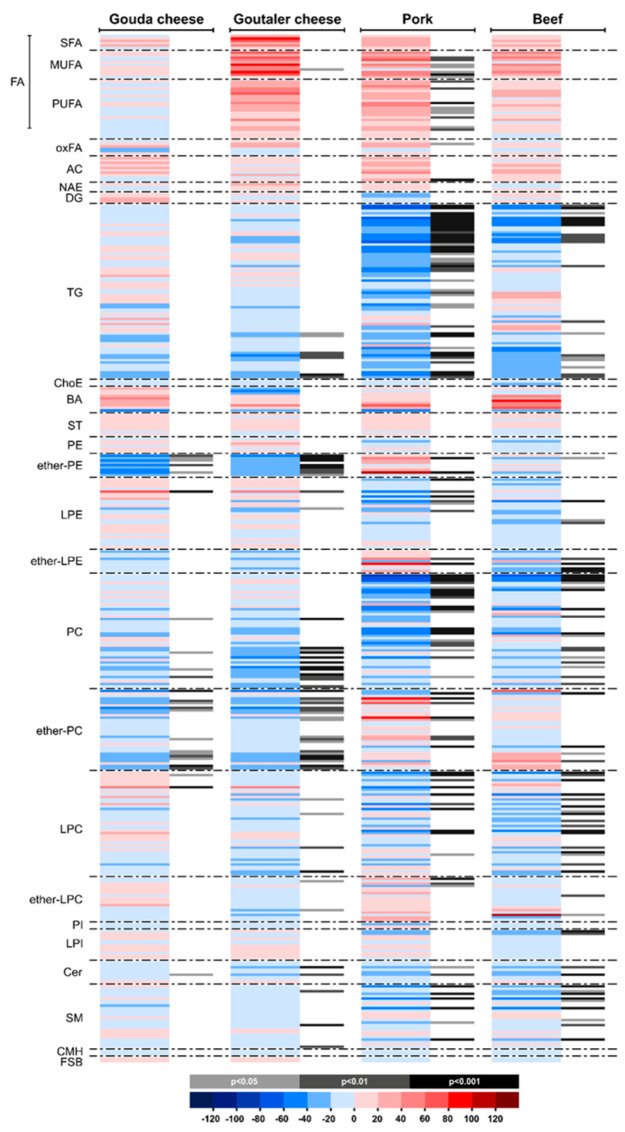
Heatmap representing the results of the lipidomics analysis for the four test diets (Gouda and Goutaler-type cheeses, pork, and beef). Data are represented as median ± SD of the percentage of change (post- versus pre-intervention values) (adjusted Wilcoxon signed-rank test, Holm–Sidak method). Abbreviations: fatty acids (FAs); saturated FAs (SFAs); monounsaturated FAs (MUFAs); polyunsaturated FAs (PUFAs); oxidized FAs (oxFAs); acylcarnitines (ACs); N-acyl ethanolamines (NAEs); diglycerides (DGs); triglycerides (TGs); cholesteryl esters (ChoEs); bile acids (BAs); steroids (STs); phosphatidylethanolamines (PEs); ether-linked PE and vinyl-ether-linked PE (ether PE); lysoPE (LPE); ether-linked LPE and vinyl-ether-linked LPE (ether LPE); phosphatidylcholine (PC); ether-linked PC and vinyl–ether-linked PC (ether PC); lysophosphatidylcholines (LPCs); ether-linked LPC and vinyl-ether-linked LPC (ether LPC); phosphatidylinositols (PIs); lyso-PI (LPI); ceramides (Cers); sphingomyelins (SMs); monohexosylceramides (CMHs); free sphingoid bases (FSBs).

Figure 2 illustrates the serum lipidome alteration during the four different dietary interventions. When comparing the data from before and after the intervention periods, we observed that the pork test diet had a profound impact on the serum lipidomic profile. Out of the 421 lipids investigated, 247 lipids differed after the pork diet (nominal *p* < 0.05, Supplementary Table S5). Of these lipids, 124 passed significance at the selected false discovery rate (FDR) threshold of 0.05. After beef test diet interventions, 223 lipids were altered (nominal *p* < 0.05, Supplementary Table S6), and 84 of these lipids passed significance at the selected FDR threshold. Similarly, 181 and 220 individual lipids were modulated after the Gouda- and Goutaler-type cheese test diets, respectively (nominal *p* < 0.05, Supplementary Tables S7 and S8, respectively). After the FDR adjustment, 27 and 64 lipids remained altered following the Gouda- and Goutaler-type cheese diets, respectively (Figure 2 and Supplementary Tables S7 and S8).

The lipid class-wise percentage of change before and after intervention for each diet is depicted in Figure 2. We found that mono- and polyunsaturated free fatty acids were increased after the pork test diet, while the free fatty acid (FFA) 18:1n increased after the Goutaler-type cheese test diet (Figure 2; Supplementary Figure S2). In addition, the pork test diet elevated the serum concentration of the oxidized fatty acid hydroxyoctadecadienoic acid (HODE) and acylcarnitine 18:1 n-9 (Figure 2). There were no significant (FDR threshold of 0.05) differences in diacylglycerol (DG) levels after the intervention; however, important changes were observed in TGs and ether lipids after the test diets. In particular, TGs were significantly reduced after the pork diet intervention, while some ether-linked lipids remained upregulated (Figure 2). Low levels of sphingomyelins and ceramides were observed after the beef and pork test diets (Figure 2).

**Figure 3:**
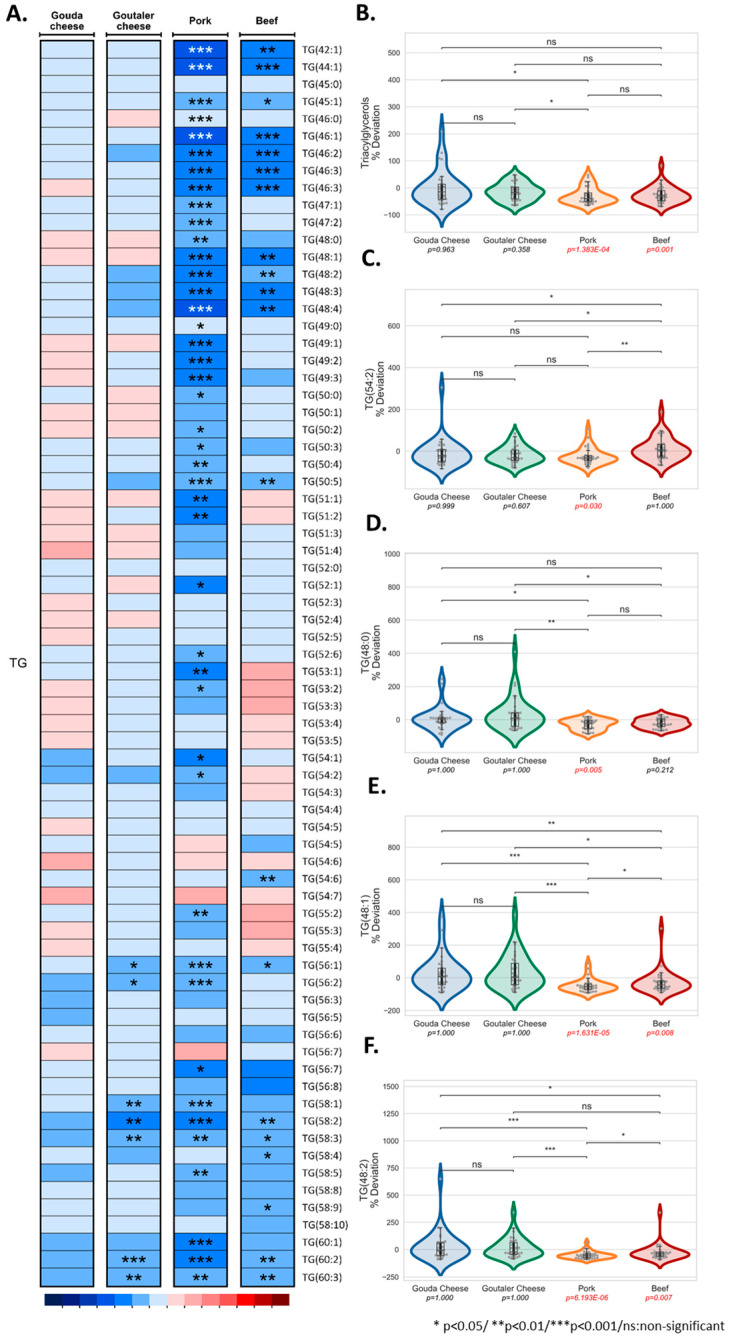
Heatmap representing the results (median ± SD of the percentage of change post- versus pre-intervention values) of the triglycerides from the lipidomic analysis for the four test diets (Gouda- and Goutaler-type cheeses, pork, and beef) (**A**), TG class (**B**) and TG species 54:2 (**C**), 48:0 (**D**), 48:1 (**E**), and 48:2 (**F**) comparisons between test diets.

Figure 3A highlights the individual circulating TG species analyzed before and after each intervention. Interestingly, 43 out of 72 TGs were significantly downregulated after the pork test diet, while 20 were decreased after the beef test diet intake (Figure 3A). For the Goutaler-type cheese test diet, a few TGs were altered. However, no clear changes with respect to the Gouda-type cheese test diet were observed (Figure 3A). At the lipid class level, only the pork and beef test diets promoted significant decreases in TG species (Figure 3B). Of all the downregulated TGs, decreased levels of TG 54:2 (Figure 3C) and TG 48:0 (Figure 3D) after the pork test diet (Figure 3E) may be of metabolic significance, as these have previously been associated with CVD and hypertension. In addition, both the pork and beef test diets also reduced TGs 48:1 (Figure 3E) and 48:2 (Figure 3F), which have been linked to hypertension.

**Figure 4:**
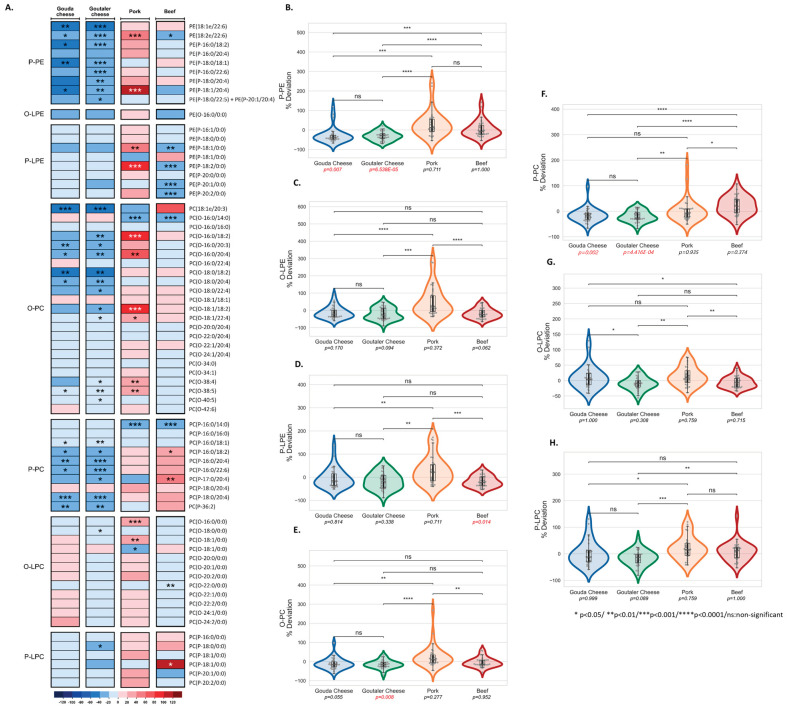
Heatmap representing the results (median ± SD of the percentage of change post- versus pre-intervention values) of the ether lipids from the lipidomic analysis for the four test diets (Gouda- and Goutaler-type cheeses, pork, and beef) (**A**). Comparisons between test diets for lipid classes (**B**–**H**). Abbreviations: phosphatidylethanolamines (PEs); ether-linked PE (O-PE); vinyl–ether-linked PE (P-PE); lysoPE (LPE); ether-linked LPE (O-LPE); vinyl-ether-linked LPE (P-LPE); phosphatidylcholine (PC); ether-linked PC (O-PC); vinyl-ether-linked PC (P-PC); lysophosphatidylcholines (LPCs); ether-linked LPC (O-LPC); vinyl-ether-linked LPC (P-LPC).

Furthermore, we observed that pork consumption had a profound impact on ether phospholipid species (Figure 4A). The heatmap depicts that pork consumption upregulated some vinyl-ether-linked phospholipids (P-PC, P-LPE, and P-PE; also known as plasmalogens) and ether-linked phospholipids (O-PC and O-LPC). On the other hand, the cheese test diets had opposite effects when compared to the pork test diet. We noticed contrasting results for PE P-18:1/20:4, PC O-16:0/18:2, and PC O-38:5. Moreover, Goutaler-type cheese and pork showed contrasting results for a total of eight individual ether phospholipid species (Figure 4A). At the lipid-class level, the P-PEs and P-PCs were downregulated after the cheese test diets (Figure 4B,F) whereas O-PCs were reduced only after the Goutaler-type cheese diet (Figure 4E). Interestingly, the pork test diet showed a non-significant trend of an increase in the O-LPE class, and this was significantly different for the other test diets (Figure 4C). No changes were detected for the O-LPC (Figure 4G) and P-LPC classes (Figure 4H); however, P-LPE (Figure 4F) was reduced after the beef test diet (Figure 4D).

The heatmap for ceramides is shown in Supplementary Figure S3A. Although the ceramide class showed no significant differences after the test diets (Supplementary Figure S3B), we observed that all interventions significantly downregulated Cer 18:1/24:0 (Supplementary Figure S3C), while all but the Gouda diet downregulated Cer 18:1/22:0 (Supplementary Figure S3D).

**Figure 5:**
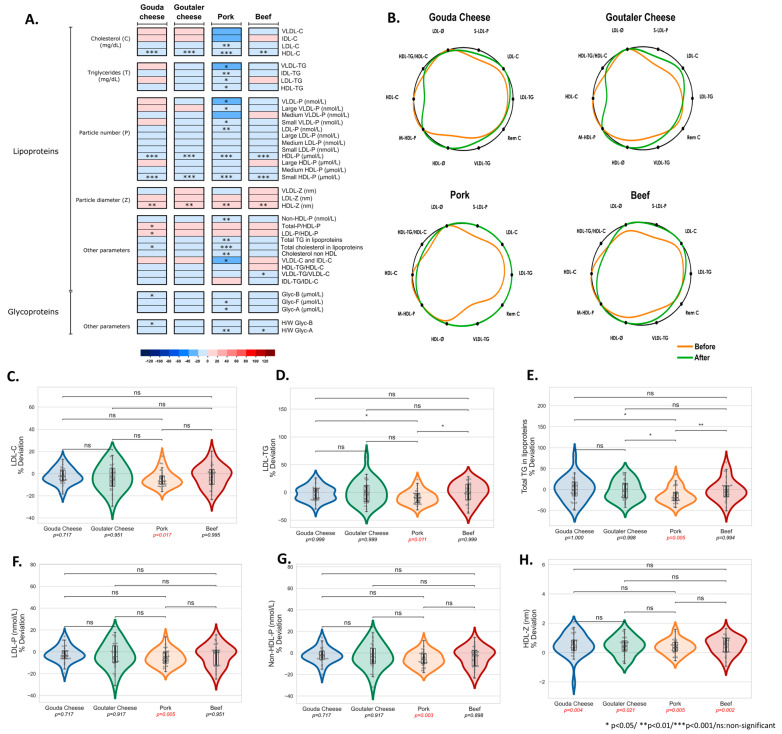
Heatmap representing lipoprotein profiles for the four different test diets (Gouda- and Goutaler-type cheeses, pork, and beef) (**A**); lipidic silhouette summarizing lipoprotein risk patterns for each test diet (**B**); comparisons between diets for LDL-cholesterol (**C**), LDL-TG (**D**), total TG in lipoproteins (**E**), LDL-P (**F**), non-HDL P (**G**), HDL particle diameter (HDL-Z) (**H**).

Then, we sought to determine the impact of the intervention test diets on the lipoprotein profile using NMR spectroscopy (Figure 5). The heatmap presented shows the percentage of fold changes when comparing the data from before and after test diets (Figure 5A), whereas the lipidic silhouettes illustrate the summarized lipoprotein risk profiles for each test diet (Figure 5B). We found that the pork test diet significantly reduced LDL cholesterol (Figure 5C), LDL-TG (Figure 5D), as well as total TG in lipoproteins (Figure 5E). Although no differences were detected among the test diets, pork reduced non-HDL cholesterol (Supplementary Figure S4A) and VLDL and IDL cholesterol levels (Supplementary Figure S4B), whereas all test diets downregulated HDL cholesterol after two weeks of intervention (Supplementary Figure S4C). Along the same line, all test diets downregulated the HDL particle number (HDL-P) (Supplementary Figure S4J) and small HDL-P (Supplementary Figure S4K); however, only pork decreased VLDL-TG and IDL-TG (Supplementary Figure S4D), HDL-TG (Supplementary Figure S4E), VLDL-P (Supplementary Figure S4G), small VLDL-P (Supplementary Figure S4H), large VLDL-P (Supplementary Figure S4I), LDL-P (Figure 5F), and non-HDL-P (Figure 5G). Interestingly, HDL size (HDL-z) was upregulated after all test diets (Figure 5H). The silhouette figures show that all the diets promote benefits to the summarized lipoprotein risk profile. Notably, although all test diets promoted benefits to CV risk (as the green line is closer to the outer ring for several markers), the pork test diet showed a distinct reduction in most of the CV risk markers (Figure 5B).

## 3. Discussion

In this four-arm crossover clinical trial, we showed that adding regular-fat animal products, in particular pork meat, to a healthy diet rich in fiber and micronutrients may not promote deleterious metabolic outcomes. The diets containing pork, beef, or cheese products exhibited positive health effects when compared to the participants’ habitual diets, showing benefits to classical cardiovascular risk markers, as well as two new CVD markers, such as subclasses of lipoproteins and molecular lipid species, as analyzed by lipidomics. We observed that the consumption of a healthy diet with pork meat resulted in the greatest benefits to CVD risk by improving the lipid profile, downregulating TGs and ceramide lipid species, and upregulating ether lipids, especially plasmalogens, when compared to the other test diets.

For the last 50 years, different guidelines have been recommending limiting or reducing saturated fats to less than 10% of TEE to reduce CVD risk [12,13], especially the intake of products rich in lauric, myristic, and palmitic acids that have been associated with deleterious effects [3,4,5]. Nevertheless, the evidence for the association between different sources of SFAs, such as regular-fat dairy or meat, and health outcomes in humans remains controversial [9,20,28,29]. Indeed, the data from prospective studies and meta-analyses indicate that products from different sources could have hindered the associations observed between SFAs and CVDs in humans [10,16,20,28,29] and that dairy products might be inversely correlated with diabetes and CVDs, even when regular-fat products are investigated [16]. On the other hand, most of these studies established a positive association between red meat consumption and CVDs [10,14], albeit the underlying mechanisms remain to be elucidated. Contrary to these findings, in our study, some benefits were detected in participants after the four test diets, including both the cheese and meat test diets, when compared to the participants’ habitual diets that provided similar proportions of SFAs. All test diets, except the pork test diet, led to reductions in body weight when compared to pre-intervention values, and reductions in total cholesterol were observed after the test diets, even after adjusting for body weight changes. Nevertheless, it is worth noting that the statistically significant reductions in body weight were quite minor and should not have an impact on the metabolic effects, especially not on lipids. Indeed, the beneficial effects on body weight can be at least partially attributed to the high content of fiber in the diet we provided. Nonetheless, improvements in the lipid profile were unexpected due to the high quantity of animal products given to the participants (more than 10% was from SFAs in the cheese and beef test diets). In addition, our experiment included non-obese, healthy individuals, and this could have had an impact on our findings since the majority of CVDs are obesity-related. Nevertheless, our findings are consistent with a previous study in which a cheese (96–120 g) or meat diet consumed for 3 weeks showed no effects on LDL and total cholesterol levels [30]. Similar findings were observed by others after 20 weeks of a low-carbohydrate diet, rich in SFAs (21% of TEI), whilst some studies detected increased total and LDL cholesterol levels after high-SFA diets [31,32]. Different from those studies, our test diets were healthy, rich in essential nutrients from natural sources, and had commonly used food items, providing between 8 and 14% of TEI from SFAs, which could explain the different results. Nonetheless, most of our test diets provided more than the recommended 10% of SFAs. Remarkably, we also observed reductions in apolipoprotein B, an important CVD risk marker, after the meat test diets (pork and beef), but not after the cheese interventions. This could be explained, at least in part, by the higher palmitic and myristic acid contents in the cheese products compared to the meat diets (six and ten times more in cheese than in meat, respectively) [7,33] as these fatty acids have been associated with deleterious effects in experimental studies [3,4,7,33]. In contrast to our findings, Bergeron and colleagues [31] detected increased LDL and apolipoprotein B concentrations after the consumption of a red meat diet rich in SFAs. This discrepancy may be associated with the sources of SFAs, product matrices [29], the content of SFAs (8–12% in the meat diets), or the quality of the diet provided to the participants. When we compared pre- and post-intervention values, a secondary aim of our study, other important cardiovascular risk variables were favorably modulated by all interventions. HDL particle size, which has been inversely associated with CVDs [34], was increased after all test diets, despite the recorded reductions in HDL cholesterol. Increased HDL size has been previously reported after diets rich in fatty fish or vegetable oils, but not after cheese, pork, or beef consumption [35,36,37]. In addition to HDL size, the data from the lipidomics analysis indicate that two ceramide species (Cer 18:1/22:0 and Cer 18:1/24:0) were downregulated after the test diets. Ceramides have been shown to impair insulin signaling through different pathways, directly affecting cell metabolism and increasing CVD risk [7,23,26,38,39]. Furthermore, one of the downregulated ceramides, Cer (18:1/24:0), was associated with dysglycemia in participants from the Framingham Heart Study [40]. Similar to our intervention data, ceramides were shown to be reduced after nutrient-rich diets in previous studies [26,35]; however, an increase in ceramides are usually detected after SFA consumption [24].

One of the most interesting findings of our study regards the effects of the pork test diet on lipid species and lipoprotein subclasses. Even though all test diets were healthy and provided benefits, the results from the pork diet suggested increased benefits from this product compared to others. In effect, our results shed new light on the potential CVD benefits of a healthy diet enriched with pork. Unlike the other test diets, the pork intervention showed profound metabolic benefits, improving both classical CVD markers and novel parameters, such as lipid species associated with reduced CVD risk. Intriguingly, reductions in insulin, HOMA-IR, and triglycerides were detected only on the pork test diet. Studies investigating the metabolic effects of diets with pork (and not total red meat) are limited [27,41]; however, recent data from an Australian study indicate that adding pork to a Mediterranean diet does not affect the lipoprotein profile [27]. The plausible explanation for this is that the contents of MUFAs (almost 50% of total fat content) and PUFAs (17% of total fat content), as well as a lower SFA content, in the pork meat could have influenced the outcome. Indeed, the benefits of diets rich in MUFAs and PUFAs on glucose and lipid profiles have been widely described [42,43,44]. Indeed, a recent study showed that an important part of the Mediterranean diet’s benefits could be attributed to the MUFA content [45].

We also found a beneficial impact on the lipoprotein profile after the pork diet. LDL-cholesterol and LDL-TG, as well as total TGs in lipoproteins, non-HDL cholesterol, VLDL and IDL-cholesterol, VLDL-TG, IDL-TG, HDL-TG, VLDL-P, small VLDL-P, large VLDL-P, LDL-P, and non-HDL-P were significantly reduced after the pork diet, resulting in a better overall cardiovascular risk profile. Previous studies suggest that diets high in SFAs can increase LDL cholesterol, Apo B, and total, medium-, and small-sized LDL particles after only 3 weeks [31]. However, it is noteworthy that the researchers provided 18% TEI of SFAs, compared to the 8–10% in our pork diet, and the intervention was conducted for a longer period than our study (3 weeks). In addition, pork was not the main source of SFA in that study. In fact, our results regarding lipoprotein subclasses after the pork diet were remarkable. Lipid subclasses measured with NMR are important cardiovascular risk markers because they provide a better overview of the quality of lipoproteins [36]. Moreover, reductions in some parameters, such as LDL-P, LDL-TG, and HDL-TG, have been previously associated with a decreased risk of CVDs [38]. Although improvements in the lipoprotein profile could be attributed to the consumption of a healthy diet rich in fibers and vegetables [46], this does not fully explain the differences detected among pork and other animal products in our study. The extra benefits associated with this diet could be credited, at least partially, to the distribution of fatty acids, i.e., the higher content of MUFAs and PUFAs, as well as the slightly lower content of SFAs in this diet (8–9% versus 10–14% in beef and cheese, respectively).

Furthermore, the lipidomic analysis provided evidence for the beneficial metabolic effects of the test diets, especially the pork intervention. We detected an increase in circulating MUFAs and PUFAs after the pork diet and a reduction in TG species after the intervention, several of which were saturated. The data from large-cohort studies have identified short-chain TGs with fewer double bonds as markers of increased CV risk [47,48]. Additionally, a previous study demonstrated that saturated and short-chain TG species were reduced after a weight loss program, and this change was directly associated with an increase in insulin sensitivity among individuals with insulin resistance [49]. Indeed, we observed increased reductions in TGs with either lower chains and/or fewer double bonds after the pork test diet. Interestingly, for some of the TG species, a significant reduction was also observed after the beef diet, but not after the cheese diets, indicating a beef meat-product-specific effect. Interestingly, previous studies conducted by Djekic and colleagues [50] observed lower levels of saturated TGs after a vegetarian diet when compared to a meat-based diet. Meanwhile, our finding is in line with a study in which reductions in TG species that contained odd-chain fatty acids were observed after consuming a healthy Nordic diet when compared to a control diet [26]. Similar to this study, our test diets provided elevated consumption levels of MUFA, PUFA, and fiber when compared to the participants’ habitual diets. Interestingly, in our study, reductions in TG species that have been directly associated with CVD risk were detected after the pork diet intervention. TG 54:2, which has been associated with a higher CVD risk [51], and TG 48:0, which has been associated with hypertension [52,53], were reduced only after the pork intervention, while both pork and beef test diets downregulated the TG species 48:1 and 48:2, which have also been shown to be correlated with hypertension [52,53].

One of the most unexpected findings from our study is related to the effects of the test diets on ether phospholipids. We observed opposing results when comparing meat (mainly pork) and cheese diets, the latter showing a trend of decreases in several P-PE and P-PC species. On the other hand, the pork diet, and in some cases the beef diet, were shown to upregulate P-PE, P-PC, P-LPE, O-LPC, and O-PC species. Several important metabolic functions have been attributed to plasmalogens (P-LPE, P-PE, and P-PC species) [25]. It is suggested that the vinyl-ether linkage of the plasmalogens can be oxidized by reactive oxygen species and that they could play a role in protecting lipids’ membranes from oxidation [25,54]. Using a protocol of a high-fat diet enriched with lard for eight weeks in rodents, Gowda and colleagues observed reductions in some lipid species, such as PC and ethereal PC, but not LPC, PE, and LPE [55]. Contrary to our results, the HFD used in this study was rich in palmitic and stearic acids, which was not observed for our pork meat. Moreover, the authors observed that PUFA-derived lipids were inversely associated with obesity, which could also explain some of our findings [55]. In humans, the data from cohorts evidenced negative associations between plasmalogens and CVDs [25,56]. In addition, inverse associations between both ether- and vinyl-ether-linked PC species (P-PC and O-PC species) with prediabetes and type 2 diabetes have been reported [56]. Similarly, one study that investigated the effects of a Nordic diet rich in unsaturated fats and fiber showed an upregulation of plasmalogens after 12 but not 18 weeks [26]. Additionally, the changes were positively associated with n-3 and n-6 intakes. It is somewhat possible that the higher content of PUFAs in the pork diet (9–10% versus 7–8% of TEI), as well as the MUFAs (18% versus 13–15%), or the lower content of SFAs in this diet (8–9% versus 12–14%), could have to some extent influenced these results. Altogether, our data suggest that animal products, which are important sources of SFAs, should not be classified as having similar effects, as their composition can directly affect metabolic outcomes.

It is worth mentioning that we detected differences in the content of micronutrients when comparing the habitual diet of the participants, and that most of the nutrients were higher in the test diets. Even though we cannot exclude that some of these nutrients may have influenced our results, most values were within the normal range for both habitual and test diets and, therefore, are not expected to promote metabolic changes of great magnitude within two weeks. Indeed, a recent meta-analysis showed that only folic acid and omega-3 FA supplementation provided high-quality evidence for reducing CVD risk [57]. Moreover, very few differences were detected among the four test diets. Some micronutrients were higher in the cheese test diets (such as calcium), whereas others in the pork and/or beef diets (such as iron), which could not fully explain the differences observed among the test diets, especially regarding the pork diet.

Nonetheless, not all our results were favorable to CVD risk. Reductions in HDL cholesterol, HDL-P, and small HDL-P, as well as apolipoprotein A, were detected after all test diets to a similar degree. Most studies investigating the effects of diets rich in either SFAs, meat, or cheese reported increases in HDL and apolipoprotein A [5,18,20,58,59], even when the participants received a low carbohydrate/high SFA (21% TEI) dietary intervention [60], which was not the case in this study. Unlike our study, Ebbeling and colleagues detected elevated, large HDL-P after the low-carbohydrate/high-SFA diet when compared to a high-carbohydrate control diet. Furthermore, an increase in uric acid after pork and beef test diets, but not after cheese diets, was detected in our study. Elevated uric acid has been reported to be associated with a higher serum total antioxidant capacity in patients with atherosclerosis, which might indicate a mechanism to reduce oxidative damage [61]. Thus, the observed uric acid reduction after the cheese diets could help explain the underlying mechanisms linking dairy intake and reduced risk for CVDs. It is noteworthy that it is not clear whether these negative effects could counterbalance the benefits of the test diets.

Our study had some limitations. Although we had a strong study design, with four different test diets in a crossover design that allowed us to have a baseline/control diet for each intervention, we could not objectively assess the participant’s compliance with the intervention periods. However, the participants reported no major problems with test products after the intervention periods. Additionally, we obtained information regarding all washout period diets, and no differences were detected throughout the periods, which indicates that the participants went back to their habitual diet during the washout periods and that we provided them with a diet with better nutritional quality. Even though a run-in period and/or controlled washout periods would be ideal, all participants were instructed to maintain their habitual diets and their compliance was confirmed by the diet registration information they provided. Although it might be suggested that the metabolic benefits detected can be attributed to a controlled diet, the participants were allowed to control their energy intake by changing their carbohydrate consumption, and no changes in total energy intake were detected when comparing the test diets with the habitual diet. Furthermore, we assessed both the classical and novel markers of CVD risks, such as lipid species and subclasses of lipoproteins, which yielded important results regarding the effects of the intake of animal products, especially after the pork test diet. Even though three of the test diets promoted weight loss, the absolute values were low, with an average reduction of less than one kilogram (in participants that were mostly lean and healthy according to the initial metabolic profile). Moreover, this small change in body weight does not explain the benefits detected in our study as small changes in body weight have been shown to affect the lipid profile at a low magnitude [62]. In addition, it is worth noting that the pork diet, which showed the greatest benefits, had no significant effect on body weight. However, it is not clear whether the metabolic changes observed could have been different if the participants of the study were older, in the overweight/obese categories, and/or had high CV risks.

## 4. Material and Methods

The intervention study was ethically assessed and approved by the Regional Committee for Medical and Health Research Ethics, REK south-east, Norway, case number 139404. Participants had to sign a consent form before participating in the intervention study. In addition, they were able to withdraw their consent at any time without justification. If they withdrew, their health information and biological material were not researched further. The study was conducted according to the declaration of Helsinki and registered as a clinical trial in the ISRCTN registry (ISRCTN39863778).

### 4.1. Participants

All volunteers were recruited at the Norwegian University of Life Sciences (NMBU), in Ås, Norway. The recruitment was conducted with the help of Internet advertising, as well as information posters distributed at the university campus.

**Figure 6:**
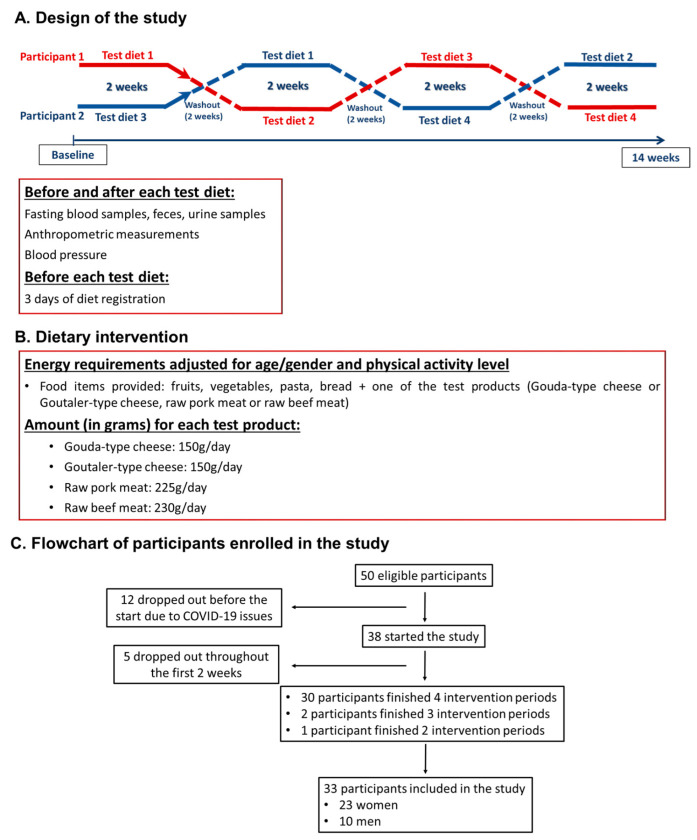
Design of the study (**A**), dietary intervention protocol (**B**), and flowchart of participants enrolled in the study (**C**).

A total of 50 people were recruited (12 men and 38 women), and 33 were included in this analysis (23 women and 10 men, Figure 6C). The higher number of dropouts (*n* = 12) occurred before the study started, due to enhanced COVID-19 restriction measures at baseline.

### 4.2. Inclusion Criteria

Inclusion criteria were men and women aged between 18 and 40 years with a BMI between 18.5 and 30 kg/m^2^, who were healthy, and who performed less than 10 h of moderate/intense physical activity per week.

### 4.3. Exclusion Criteria

Volunteers who were taking any medication except for birth control pills, who did not consume meat and/or dairy products, who were trying to lose weight, or who had food allergies were excluded. In addition, during the first blood withdrawal, participants with vein problems that could affect blood withdrawal were also excluded. Because the length of the menstrual cycle is usually around 28 days, all our test diets were administered in the same phase of the cycle.

### 4.4. Design of the Study and Research Protocol

This study was a four-arm crossover clinical trial in which participants were assigned to four test diets in a random order (Figure 6A) in a Latin square design. Each test diet was conducted for 14 days, followed by 2 weeks of washout between them; thus, the total duration of the study was 14 weeks (3.5 months). This means each test diet had its own control as in pre-intervention values (either baseline or washout values depending on the test diet order). The study was conducted between January and May 2021 (winter and spring), and the last participant was enrolled at the beginning of March.

At the beginning and end of all test diets, the participants attended university facilities to deliver urine and feces samples and to provide blood, anthropometric measurements, and blood pressure data (Figure 6A) on eight different occasions (before and after each test diet). All clinical data were collected on weekdays (Tuesday to Friday). All food items were delivered in plastic bags tagged with the participant’s code (according to their energy needs). The food items were delivered to the NMBU campus once a week and, therefore, they had to pick up the food twice for each intervention. In case the participant had problems coming to the university once a week, they received all food items for two weeks. To increase the adherence to the protocol, researchers provided recipes with all food items.

### 4.5. Test Diets

All participants received a healthy test diet, with a low percentage of ready-to-eat foods and foods with a high degree of processing (less than 20% of TEI), and with food ingredients regularly consumed in Norway: apple, avocado, bouillon powder, wholegrain bread, carrot, celery root, garlic, strawberry jam, margarine, rapeseed oil, onion, orange, parsnip, wholegrain pasta, oatmeal, salmon spread, squash, and tomato. In addition, participants received iodized salt (2 g per week) and vitamin D pearls (15 µg) along with one of the four animal test products: Gouda-type cheese (150 g/day), Goutaler-type cheese (150 g/day), raw pork meat (225 g/day), or raw beef meat (230 g/day) (Figure 6B). The amounts of animal test products were higher than what is usually consumed, since the aim of the study was to investigate the differences between the different animal fats. All test products had similar energy contents, and the amounts were determined to match the same macronutrient distribution. For raw pork and beef, a reduction of 20% of the total weight of the product was expected after the heating process. The amounts of the test products were the same for all participants; however the other food items (fruits, vegetables, oil, and pasta) were adjusted to individual energy needs to maintain the macronutrient composition, as well as the distribution of fats (Figure 6B; Supplementary Tables S9–S11). The energy requirements were determined according to a Norwegian online diet planner that provides information about food and health from the Norwegian Health and Food Authorities (kostholdsplanleggeren.no). To calculate the energy requirements, age, sex, and self-reported physical activity level were considered. Additionally, physical activity level was assessed with the International Physical Activity Questionnaire (IPAQ) [63] that was filled out by the participants at baseline and at the end of the study.

Based on the energy requirements of participants, six groups were created: three for women (F1, F2, and F3) and three for men (M1, M2, and M3). Of the 23 women included, 15 were classified as F2 (2300 kcal/day), 6 as F3 (2600 kcal/day), and 1 as F1 (2200 kcal/day). Among men, seven were classified as M2 (3000/day kcal), two as M3 (3600/day kcal), and one as M1 (2800/day kcal). The nutritional composition of the four test diets provided to groups F2 and M2 (the most frequent groups) and nutritional analysis of one test diet (Gouda cheese) for groups M1, M3, F1, and F3, and all food items provided to participants in groups F2 and M2 (in grams/day or units/day) are depicted in Table 2 and Supplementary Tables S9 and S10, respectively. Due to the fact that not all participants within each group had the exact same energy requirements, they were instructed to adjust their energy intake by slightly increasing or reducing the intake of carbohydrates (pasta and bread).

Apart from the food items described in the Supplementary Table S10, participants were allowed to consume coffee and tea of up to a maximum of 5 cups per day and to abstain from alcohol and soft drinks (especially those with added sugar). In specific situations when the participant had difficulties with adherence, one glass of sugar-free soda per day was allowed. Participants were advised to consume four meals per day, distributing the test product between two and three meals per day. They were also instructed to eat all the test products and inform the researchers if they had any problems with them. Those who found it difficult to eat all test products were asked to estimate deviations. This instruction was continuously given to the participants who stayed sufficiently close to the requested test dietary pattern.

Table 2 shows the energy intake and macronutrient distribution ranges. The energy intake and macronutrient composition range of the test diets for groups F2 (2300 kcal/day) and M2 (3000 kcal/day) were as follows: total energy (in kcal): 2.6% variation between test diets for women and 2.0% variation between test diets for men. Regarding the macronutrient distribution, the test diets presented a variation between 46 to 48% of energy intake from carbohydrates, 15 to 16% of total energy from proteins, and 37–38% of total energy from fat. The proportion of SFAs varied between 8 and 14% of TEI, while monounsaturated fatty acids (MUFAs) represented 13–18% of TEI. Additionally, PUFA intake corresponded to 7 to 9% of TEI.

The test products given to participants provided the following energy values per day: Gouda-type cheese 528 kcal, Goutaler-type cheese 527 kcal, pork 547 kcal, and beef 504 kcal. The food composition analysis is detailed in the Supplementary File. In the test diets, a healthy diet with recommended amounts of vitamins and minerals according to the Nordic Nutrition Recommendations [13] was provided. In addition, as the intakes of iodine and vitamin D were known to be deficient, both micronutrients were supplemented. Participants received Vitamin D pearls from Pharma Nord© throughout the study (14 weeks) and were instructed to take three vitamin D pearls/day (15 µg/day), including washout periods. In addition, iodized salt produced in Denmark for the Swedish market (Jozo©, Hadsundvej 17, 9550 Mariager, Denmark) was given to participants every week (2 g/day, 14 g/week). The salt from Sweden has 50 µg iodine/g salt, while the Norwegian salt has 10 times less.

Although all test diets included one animal product and they had almost identical macro- and micronutrient compositions, there were differences regarding the proportion of different fatty acids among the test products. The test products provided around 40 g of fat (ranging between 37 g from beef to 41.4 g from pork), but the proportion of SFAs was higher for the cheese test products (27.5 and 27.3 g for Gouda- and Goutaler-types, respectively) compared to pork and beef (14.0 and 17.0 g, respectively). On the other hand, MUFA was higher in pork meat (19.4 g compared to 8.1 and 7.8 g for Gouda- and Goutaler-type cheeses, respectively, and 14.7 g for beef). In addition, both cheese products provided 0.8 g of PUFAs, which was similar to beef (1.0 g), but much less than pork, which comprised 7.0 g of PUFAs in its composition.

### 4.6. Food Composition Analysis

The food composition analysis is detailed in the Supplementary File [64,65].

### 4.7. Diet Assessment

At the baseline and during each of the three washout periods, all participants had to complete three days of diet registrations. They were instructed to register their daily intake of food and beverages online at the Norwegian diet planner (kostholdsplanleggeren.no). During the washout periods, the participants were instructed to register their diets from “normal” days and to avoid registrations from “special” days (holidays, celebrations, etc.), when their diets were expected to deviate from normal. Due to COVID-19 restrictions, social activities were rare during the intervention period. The diet registrations were from recurring days.

### 4.8. Health Questionnaire, Risk Analysis, and Physical Activity

A health questionnaire was given to all participants before each test diet, and its content and the risk analysis are detailed in the Supplementary File [66].

Physical activity level was assessed at the beginning and end of the study with the IPAQ [63]. Leisure time and total physical activity (in minutes/week), as well as sitting time, were analyzed.

### 4.9. Anthropometric Measurementsand Clinical Variables

Height was measured in centimeters to one decimal place using a portable stadiometer (Charder HM200P Portstad). Weight was measured in kilograms to one decimal place using the Tanita TBF-300A Body Composition Analyzer scale and with participants wearing light clothes and no shoes. Waist circumference was measured by trained staff at the midpoint between the lowest rib and iliac crest using a Seca 203 Ergonomic Circumference Measuring Tape.

Weight and waist circumference, blood pressure, and blood glucose were recorded before and after each 14-day intervention sequence. Height was measured once, at baseline. Blood pressure was measured using an A&D medical automatic blood pressure monitor (A&D, Tokyo, Japan). After participants rested for 10 min in a sitting position, three consecutive measurements were taken, and the average of the three measurements was used.

If the participant had any contact with anyone who had tested positive for COVID-19 in the previous days, waist circumference and blood pressure measurements were not taken to avoid an increased risk of staff infection.

### 4.10. Blood Sampling

Participants’ blood samples were taken before and after each test diet period, after 12 h of fasting. In addition, the participants provided urine and feces samples. The blood samples were processed on the recommendation of the Fürst medical laboratory (Norway) and/or according to the procedures for lipidomics analysis at OWL Metabolomics (Derio, Spain). The tubes were centrifuged in a swing-out centrifuge (after 0.5–1 h) at 1500 g for 12 min. After the serum and plasma were separated into cryotubes, they were placed in a −80 °C freezer until analysis.

### 4.11. Analytical Procedures

Fasting glucose, insulin, C-peptide, total serum triacylglycerol, HDL-cholesterol, LDL-cholesterol, apolipoprotein A, apolipoprotein B, alanine transaminase (ALT), aspartate transaminase (AST), C-reactive protein, uric acid, calcium, ferritin, iron, vitamin D, and vitamin B12 were measured using accredited methods at a commercial medical laboratory in Norway (Fürst Medical Laboratory, Oslo, Norway), which also provided the method codes and analytical coefficient of variation.

Interleukin 1β (IL-1 β) and interleukin-6 (IL-6) were analyzed using R&D enzyme-linked immunosorbent assays (ELISAs) (R&D Systems, Minneapolis, MN, USA).

### 4.12. Lipidomics and Lipoprotein Profile

Lipidomics and lipoprotein profile NMR were performed at OWL Metabolomics (Spain). The detailed procedures are described in the Supplementary File [67,68,69,70,71,72].

### 4.13. Power Calculation

The power calculation was based on other studies investigating the effects of 2-week dietary interventions on health producing changes in the lipid profile as the main outcome [73]. Based on our own data, we calculated the sample size estimating a change of 0.3 mmol LDL cholesterol /L serum and a standard deviation of 0.8 mmol LDL cholesterol/L serum to ensure an 80% chance of ending up with a *p*-value less than 0.05 (estimated sample of 32 participants). A dropout of 15% was assumed based on former intervention studies of this group and, therefore, to reach the estimated sample size, 37 subjects were deemed necessary. Since we had 4 intervention diets, the closer multiple of 4 was selected (40 participants).

### 4.14. Statistical Analyses: Clinical and Biochemical Data

IBM SPSS Statistics for Windows, Version 27.0. (IBM Corp, Armonk, NY, USA) and GraphPad Prism version 9.0 for Windows (GraphPad Software, San Diego, CA, USA) were used for statistical analyses. Data are presented as means and standard deviations or standard errors of the mean (for figures). The Kolmogorov–Smirnov test was used to analyze the distribution of the data, and for variables without a normal distribution, a non-parametric test was performed. To investigate the effects of each test diet (pre/post comparisons) on clinical variables, a repeated-measures ANOVA (RM-ANOVA) was employed using sex and body weight change as covariates and the order of the intervention as a between-subject factor (for each test diet separately). To compare the effects of the four different test diets (a/b/c/d comparisons) on the clinical variables, the percentage of change from baseline to the end of each intervention period was calculated (100 × (after − before)/before). Thus, a mixed-effect model for repeated measures with sex and diet as the fixed effects and body weight change and intervention order as the random effects was employed. Adjustments for multiple comparisons were performed using the Sidak method. For all analyses, *p* < 0.05 was considered significant.

### 4.15. Lipidomics and NMR Lipoprotein Profile Data

Data are represented as median ± SD of the percentage of change, calculated as 100 × (After−Before)/before. Differences between paired diets (before and after each diet) were tested using the adjusted Wilcoxon signed-rank test (Holm–Sidak method). Differences among diets, as a percentage of the changes, were evaluated using the Kruskal–Wallis H-test. A false discovery rate threshold of *p* < 0.05 was used. Calculations were performed using Python v3.7.4. The statistical analyses were performed using pandas v1.1.3 [70], NumPy v1.20.3, SciPy v1.5.2 5 [71], and Seaborn library v0.11.1 6 [73].

Since no differences were detected between males and females at the baseline visit in the multivariate data analysis and the number of women was much higher to the number of men, no sex-specific analysis was conducted.

## 5. Conclusions

In summary, our study showed that, in the context of a healthy diet rich in micronutrients and fiber, consuming regular-fat animal products, in particular pork, had no major adverse effects on the classical and novel CVD risk markers. On the contrary, we observed that the test diets consumed promoted CVD benefits after only two weeks when compared to the participant’s habitual diet, which reinforces the importance of investigating the effects of animal fats in the context of healthy diets. Remarkably, we showed here that the consumption of a healthy diet including pork meat led to numerous metabolic benefits, including improvements to lipoprotein subclasses, reductions in lipid species associated with CVDs, and the upregulation of some plasmalogens, which play an important role as endogenous antioxidants. Our study suggests that the quality of the diet is more important than the restriction of regular-fat animal products, at least for this age group. Moreover, our findings indicate that the composition of the pork meat is capable of promoting increased benefits when compared to the other animal products analyzed. Although it is difficult to generalize our findings to other populations, our data indicate that the consumption of regular-fat animal products, especially unprocessed pork meat, within the context of nutrient-dense diets, should not be discouraged as a measure to reduce CVD risk. Further studies with similar designs, but including longer intervention periods, are needed to confirm our findings.

## Figures and Tables

**Table 1:** Baseline clinical, dietary, and physical activity data according to sex.

	Men	Women	*p*-Value	Total Sample
Clinical Data				
Number (n; %)	10 (30%)	23 (70%)	.	33
Age (years)	24.6 ± 2.4	23.1 ± 2.7	0.15	23.6 ± 2.7
Height (m)	1.8 ± 0.1	1.7 ± 0.1	<0.01	1.7 ± 0.1
Weight (kg)	87.7 ± 14.5	68.1 ± 10.8	<0.01	74.0 ± 14.9
BMI (kg/m^2^)	27.0 ± 3.7	24.3 ± 3.1	0.04	25.1 ± 3.5
Waist circumference (cm)	87.7 ± 8.1	76.9 ± 7.1	<0.01	79.8 ± 8.7
Systolic blood pressure (mmHg)	132.4 ± 8.5	119.7 ± 10.4	<0.01	123.6 ± 11.4
Diastolic Blood pressure (mmHg) ^&^	79.7 ± 9.5	79.1 ± 8.5	0.74	79.3 ± 8.7
Glucose (mmol/L)	4.9 ± 0.3	4.6 ± 0.3	0.04	4.7 ± 0.3
Insulin (µUI/mL)	7.1 ± 3.2	8.3 ± 4.1	0.41	7.9 ± 3.9
HOMA-IR	1.5 ± 0.7	1.7 ± 0.9	0.57	1.7 ± 0.9
C-peptide (pmol/L)	333.6 ± 92.1	356.3 ± 87.5	0.51	349.4 ± 88.1
AST (U/L) ^&^	22.3 ± 9.2	16.5 ± 6.9	0.01	18.2 ± 8.0
ALT (U/L) ^&^	28.1 ± 11.3	18.9 ± 9.3	<0.01	21.7 ± 10.7
Total cholesterol (mmol/L)	4.7 ± 1.1	4.6 ± 0.9	0.89	4.6 ± 0.9
LDL cholesterol (mmol/L)	3.0 ± 1.1	2.8 ± 0.8	0.64	2.9 ± 0.9
HDL cholesterol (mmol/L)	1.4 ± 0.3	1.6 ± 0.3	0.21	1.5 ± 0.3
Triglycerides (mmol/L) ^&^	1.0 ± 0.6	1.0 ± 0.3	0.71	1.0 ± 0.4
Apolipoprotein A (g/L)	1.3 ± 0.2	1.4 ± 0.2	0.25	1.3 ± 0.2
Apolipoprotein B (g/L)	0.8 ± 0.2	0.8 ± 0.2	0.77	0.8 ± 0.2
ApoB/A ratio ^&^	0.6 ± 0.2	0.6 ± 0.1	0.38	0.6 ± 0.1
C-reactive protein (mg/L) ^&^	1.2 ± 1.9	1.1 ± 1.2	0.55	1.1 ± 1.4
Uric acid	391.9 ± 70.3	277.5 ± 40.9	<0.01	312.2 ± 73.4
Calcium (mmol/L)	2.4 ± 0.1	2.3 ± 0.1	0.01	2.4 ±0.1
Ferritin (µg/L) ^&^	144.5 ± 64	50.9 ± 28.1	<0.01	79.2 ± 60.0
Iron (µmol/L)	21.2 ± 6	23.6 ± 10.1	0.42	22.8 ± 9.0
Vitamin D (nmol/L)	48.2 ± 18.3	51.0 ± 19.0	0.70	50.1 ± 18.5
Vitamin B12 (pmol/L) ^&^	344.2 ± 140.1	275.6 ± 110.6	0.14	296.4 ± 122.3

Independent samples *t*-test. ^&^: non-parametric test. HOMA-IR: homeostatic model assessment for insulin resistance; AST: aspartate transaminase; ALT: alanine transaminase; ApoB/A ratio: apolipoprotein B/A ratio.

**Table 2:** Test diet composition according to groups F2 (women, 2300 kcal, *n* = 15) and M2 (men, 3000 kcal, *n* = 7).

	Women F2 (2300 kcal, *n* = 15)				Men M2 (3000 kcal, *n* = 7)			
	Gouda Cheese	Goutaler Cheese	Pork	Beef	Gouda Cheese	Goutaler Cheese	Pork	Beef
Energy (kcal)	2289	2289	2254	2230	3029	3029	2994	2970
Carbohydrates (gram)	283.2	283.2	283.2	283.2	382.9	382.9	382.9	382.9
Carbohydrates (% of TEI)	46.0	46.0	47.5	47.5	47.0	47.0	47.0	47.0
Protein (gram)	93.2	94.4	89.3	92.9	117.1	118.1	113.0	116.8
Protein (% of TEI)	16.0	16.0	16.0	16.0	15.0	15.5	15.5	16
Fat (gram)	96.5	96.2	94.2	90.2	128.1	127.5	125.8	121.9
Fat (% of TEI)	38.0	38.0	37.5	36.5	38.0	37.5	37.5	37.0
Saturated fatty acids (gram)	35.8	35.7	22.3	25.4	40.7	40.5	27.2	30.3
Saturated fatty acids (% TEI)	14.0	14.0	9.0	10.0	12.0	12.0	8.0	9.0
Trans FA (g)	0.5	0.5	0	0.7	0.5	0.5	0	0.7
Trans FA (% TEI)	0.2	0.2	0	0.3	0.1	0.1	0	0.2
MUFA (gram)	33.4	33.1	44.6	40.0	48.9	48.6	60.2	55.5
MUFA (%TEI)	13.0	13.0	18.0	16.0	15.0	14.0	18.0	17.0
PUFA (gram)	18.0	18.0	24.1	18.0	26.6	26.6	32.8	26.8
PUFA (%TEI)	7.0	7.0	10.0	7.0	8.0	8.0	10.0	8.0
Omega-3 (gram) ^&^	3.3	3.3	3.5	3.3	5.1	5.1	5.3	5.2
Omega-6 (gram) ^&^	13.1	13.1	13.1	13.2	19.6	19.6	19.6	19.6
Cholesterol (mg) ^&^	135	135	128	165	162	162	155	192
Starch (gram) ^&^	156.2	156.2	156.2	156.2	212.8	212.8	212.8	212.8
Mono- and dissaccharides (gram) ^&^	91.9	91.9	91.9	91.9	117	117	117	117
Sugar, added (gram) ^&^	24.1	24.1	24.1	24.1	24.7	24.7	24.7	24.7
Fiber (gram) ^&^	42	42	42	42	60	60	60	60
Salt (gram) ^&^	10.6	10.5	9.1	9.3	11.5	11.4	9.9	10.2
Alcohol (gram)^&^	0	0	0	0	0	0	0	0
Beta-carotene (µg) ^&^	8736	8729	8490	8619	9039	9032	8792	8922
Calcium (mg)	1312	1443	329	334	1429	1560	446	451
Copper (mg) ^&^	1.9	1.9	2	1.9	2.8	2.8	2.8	2.7
Folate (µg) ^&^	361	352	301	305	509	500	446	453
Iodine (µg) ^€^	198	189	166	168	212	203	179	182
Iron (mg)	13	13	14.1	17.4	18.3	18.3	19.3	22.7
Magnesium (mg)	453	459	456	454	614	620	618	615
Niacin (mg) ^&^	20.2	20.9	31.1	28	29.8	30.5	40.4	37.6
Phosphorus (mg) ^&^	2078	2134	1731	1696	2759	2815	2413	2377
Potassium (mg)	3751	3742	4283	4205	5495	5486	6028	5949
Retinol (µg) ^&^	595	596	243	267	693	694	340	365
Riboflavin (mg) ^&^	1.1	1.1	0.9	0.9	1.4	1.4	1.1	1.2
Selenium (µg)	63	63	80	60	86	86	103	83
Sodium (mg)	3271	3385	2880	2872	3567	3681	3176	3168
Thiamin (mg) ^&^	1.7	1.8	2.7	1.7	2.3	2.4	3.3	2.3
Vitamin A (RAE) ^&^	1324	1324	952	984	1447	1447	1075	1107
Vitamin B12 (µg)	3.1	3	2.1	4.6	4.2	4.1	3.2	5.7
Vitamin B6 (mg) ^&^	1.6	1.6	2.5	2.2	2.5	2. 5	3.4	3.0
Vitamin C (mg) ^&^	159	159	159	159	191	191	191	191
Vitamin D (µg) ^&,£^	20.9	20.9	21.3	21.4	25.2	25.2	25.7	25.7
Vitamin E (alfa-TE) ^&^	18	18.3	18.7	18.1	25.7	26	26.3	25.8
Zinc (mg) ^&^	16.2	15.6	12.8	18.5	20	19.4	16.6	22.3

^&^: Calculated according to the nutrients database available at kostholdsplanleggeren.no. All other nutrients were analyzed in the test products. ^€^: All participants received 2 g/day of iodized salt from Sweden (50 μg/g salt). ^£^: All participants received 15 µg per day of vitamin D (3 vitamin D pearls from Pharma Nord© during the test diet and washout periods). TEI: total energy intake; FA: fatty acids; MUFA: monounsaturated fatty acids; PUFA: Polyunsaturated fatty acids.

## Data Availability

In agreement with the approved data protection protocol, the manuscript data that will be used for other publications are stored on an NMBU-defined server and the others will be transferred to NIR Data Storage along with the manuscript publication.

## Supplementary Materials

The following supporting information can be downloaded at: https://www.mdpi.com/article/10.3390/ijms24054941/s1.

## Abbreviations

Acylcarnitines (ACs)
Alanine transaminase(ALT)
Aspartate transaminase (AST)
Bile acids (BAs)
Body mass index (BMI)
Cardiovascular diseases (CVDs)
Ceramides (Cers)
Cholesteryl esters (ChoEs)
Diglycerides (DGs)
Fatty acids (FAs)
Free sphingoid bases (FSBs)
Homeostatic Model Assessment for insulin resistance (HOMA-IR)
Interleukin 1-beta (IL-1β)
Interleukin-6 (IL-6)
International Physical Activity Questionnaire (IPAQ)
Liquid chromatography–mass spectrometry (LC-MS)
Lyso-PE (LPE)
Lyso-PI (LPI)
Lyso-PC (LPC)
Monohexosylceramides (CMHs)
Monounsaturated FAs (MUFAs)
N-acyl ethanolamines (NAEs)
Non-communicable chronic diseases (NCDs)
Nuclear magnetic resonance (NMR)
Oxidized FAs (oxFAs)
Plasmanyl-PC or ether-linked PC (O-PC)
Plasmenyl-PC or vinyl-ether-linked PC (P-PC)
Plasmanyl-LPC or ether-linked PC (O-LPC)
Plasmenyl-LPC or vinyl-ether-linked LPC (P-LPC)
Plasmanyl-PE or ether-linked PE (O-PE)
Plasmenyl-PE or vinyl-ether-linked PE (P-PE)
Plasmanyl-LPC or ether-linked PE (O-LPE)
Plasmenyl-LPE or vinyl-ether-linked LPE (P-LPE)
Phosphatidylcholines (PCs)
Phosphatidylethanolamines (PEs)
Phosphatidylinositols (PIs)
Polyunsaturated FAs (PUFAs)
Saturated FAs (SFAs)
Sphingomyelins (SMs)
Steroids (STs)
Total energy intake (TEI)
Triglycerides (TGs)
