# Supplementary material for: Classification of Common Food Lipid Sources Regarding Healthiness Using Advanced Lipidomics: A Four-Arm Crossover Study
Source: Int J Mol Sci. 2023 Mar 3;24(5):4941. doi: 10.3390/ijms24054941 (PMC10003363; doi:10.3390/ijms24054941)
Supplement: Supplementary file 1 [file ijms-24-04941-s001.zip › ijms-2077998-supplementary.pdf]

Supplementary Material

Classification of Common Food Lipid Sources Regarding Healthiness Using Advanced Lipidomics: A Four-Arm Crossover Study

Milena Monfort-Pires [1,*], Santosh Lamichhane [2], Cristina Alonso [3], Bjørg Egelandsdal [1], Matej Orešič [2,4], Vilde Overrein Jordahl [1,5], Oda Skjølsvold [1,6], Irantzu Pérez-Ruiz [3], María Encarnación Blanco [3], Siv Skeie [1], Catia Martins [7,8,9] and Anna Haug [10]

Food composition analysis

Macro Kjeldahl was used for the measurement of the protein content for beef and pork meats, according to the AOAC method 2001.11, using a conversion factor of 6.25. Block digestion with a copper catalyst and steam distillation into boric acid, using Kjeltec 8400-automated distillation unit + support unit with scrubber (Foss, Hillerød, Denmark) took place.

For the determination of total fat, a Soxtec™ 8000 Extraction system in combination with a Foss Hydrotec™ 8000 Hydrolysis system was used according to the method of the Commission Regulation. Vitamin B12 was analyzed by Eurofins (Moss, Norway) [1]. Minerals (Na, Mg, Ca, P, Fe, Se, I) in the samples were analyzed according to the method described by Jørgensen [2].

The Fatty acid composition was analyzed at Vitas Analytical Services© (Norway). After homogenization of the meat and strips of cheese, internal standard methyl tricosanoate (Methyl-C23) solved in dichloromethane was added to the samples. Samples were then methylated with 3N Methanolic HCl at 80 degrees C for 2 hours and then 50°C overnight. FAMES were extracted to hexane, and samples were then neutralized with 3 N KOH in water. After vortex and centrifugation, the organic phase was injected into the GC. The analysis was performed on an 8890 GC system with a split/splitless injector, a 7693A automatic liquid sampler, and a flame ionization detector (Agilent Technologies, Palo Alto, CA). Separations were performed on a Varian CP7421 (200 m × 0.25 mm i.d.) column from Varian Inc.

Health Questionnaire

This questionnaire was based on the questionnaires from the Norwegian Health Survey in Oslo (HUBRO) [3]ɫ and included questions regarding health, diet, physical activity, tobacco habits, education and work, and the use of medicines. Moreover, the day before the test day, participants were asked about COVID-19 symptoms or contacts with others who had tested positive for the disease. In addition, the description of the

Risk analysis

A standard risk analysis developed for safe work in the laboratory was expanded to include two clinical safety parts. One part dealt with the risks related to blood sampling and treatment and biological sample treatment, as well as clinical staff vaccination. The other part dealt specifically with safety during the COVID-19 pandemic, i.e., the pre-triage form, personal protective equipment, traceability, and routines if an infected person was identified. All the food given to the participants was delivered by companies authorized for food production and commercial sale, so food safety was, therefore, not included in the risk analysis.

The intervention was carried out during the period when Norway experienced the highest infection rates of COVID-19, namely the vaccine-free period of 2021. Our approved risk analysis stated that a close 1:1 situation (<1 m) only could last 10 min despite a face mask being mandatory for both health workers and intervention participants. Due to this restriction, we experienced some incidences where we had to abandon anthropometric measurements/blood pressure assessment because the prioritized blood sampling had taken too long.

All participants were requested to fill in an approved pre-triage form before blood sampling. Most commonly, our participants were quarantined due to close contact with other non-participating people. If so, they were asked to take the PCR test and wait for the PCR results beyond the scheduled intervention test period. Then, they received extra test food for a maximum of 2 days and presented for blood sampling collection after that. Then, the washout period was either shortened, or the test period was started up a maximum of 2 days later. None of the participants tested positive for COVID-19 during the complete intervention.

Lipidomics:

Lipidomics was carried out at OWL Metabolomics (Spain). The detailed lipidomics procedure is described below.

Serum lipids were semi-quantified by ultra-high performance liquid chromatography coupled to mass spectrometry (UHPLC–MS). Briefly, two separate UHPLC–time-of-flight (TOF)–MS-based platforms analyzing methanol and chloroform/methanol serum extracts were used. Identified ion features in the methanol extract platform included fatty acids, lysoglycerophospholipids, free sphingolipid bases, and bile acids. The chloroform/methanol extract platform provided coverage over glycerolipids (di- and triglycerides), cholesterol esters, sphingolipids (ceramides and sphingomyelins), and glycerophospholipids (diacylglycerophospholipids and 1-ether, 2-acylglycerophospholipids). Each extract was spiked with metabolites, not detected in unspiked human serum extracts: tryptophan-d5(indole-d5), PC(13:0/0:0), NEFA(19:0), and dehydrocholic acid in methanol extract; SM(d18:1/6:0), PE(17:0/17:0), PC(19:0/19:0), TAG(13:0/13:0/13:0), TAG(17:0/17:0/17:0), Cer(d18:1/17:0) and ChoE(12:0) in chloroform/methanol extract. Specific lipid extraction procedures performed for each platform, chromatographic separation conditions, and mass spectrometric detection conditions are detailed in [4].

Lipidomics data were pre-processed using the TargetLynx application manager for MassLynx 4.1 (Waters Corp., Milford, MA). Data pre-processing generated a list of chromatographic peak areas for the lipids detected in each sample injection. An approximated linear detection range was defined for each identified metabolite, assuming similar detector response levels for all metabolites belonging to a given chemical class represented by a single standard compound. Those lipids for which more than 30% of data points were found outside their corresponding linear detection range were not used for statistical analyses. Data normalization was performed following the procedure described by Martínez-Arranz et al [5].

Lipoprotein profiling

Lipoprotein analysis was performed using the Liposcale® test (Biosfer Teslab, Reus, Spain), a two-dimensional proton nuclear magnetic resonance spectroscopy (2D-^{1}H-NMR) analysis [6].

Lipidomics data representation

Violin plots were used to represent data distribution and probability density. They included data points and internal boxplots representing median and interquartile ranges. A small random movement was applied to each point to minimize overplotting. Comparisons between pairs of groups (before and after per diet) were tested using the adjusted Wilcoxon signed-rank test (Holm–Sidak method), and among diets were performed using the adjusted Kruskal–Wallis H-test (Holm–Sidak method). Significance was defined as a p-value less than 0.05. Analyses were performed using Python and the pandas v.1.1.3[7], SciPy v1.5.2[8], and pingouin v0.3.8 libraries [9].

Arterial Contours (AC) were used to assess the impact of lipoprotein profile on CV risk. These figures represented patients' lipoprotein profiles concerning the mean and deviation of each lipid group from the 50th percentile in the reference population, before and after the interventions. The area defined by AC is inversely proportional to CV risk. There are 10 variables associated with CV risk that allows a global evaluation of the lipid metabolism: LDL-C, HDL-C, VLDL-TG, LDL-TG, Small LDL particle (LDL-P), Medium HDL-P, LDL particle diameter (LDL-Z), HDL-Z, HDL-TG/HDL-C, and Remnant-C. From these, HDL-C, Medium HDL-P, LDL-Z, and HDL-Z are negatively associated with cardiovascular risk, while the rest of them are positively associated with cardiovascular risk.

Supplementary Table S1. Example food composition (Gouda type cheese) according to groups F1, M1, F3 and M3.

	F1	***M1***	***F3***	***M3***
Kilocalories (kcal)	2239	2850	2566	3670
Fat (%TEI)	37	37	37.5	37.5
SFA (%TEI)	14	12	13	11
MUFA (%TEI)	12	13	14	15
PUFA (%TEI)	7	8	8	7
Carbohydrates (%TEI)	47	47	47.5	47.5
Protein (%TEI)	16	16	15	15

Supplementary Table S2. Food items given to participants of groups F2 and M2.

	F2	***M2***
Gouda-type cheese (g/day)	150	150
Goutaler-type cheese (g/day)	150	150
Pork (g/day)	225	225
Beef (g/day)	230	230
Apple (units/day)	1	2
Avocado (g/day)	30	86
Bouillon powder (g/day)	10	10
Bread, extra coarse (g/day)	100	156
Carrot (g/day)	143	143
Celery root (g/day)	71	71
Garlic (g/day)	71	71
Strawberry Jam, 53 % berries, 30 % sugar (g/day)	75	75
Margarine, rapeseed, and sunflower oil (g/day)	25	35
Oil, rapeseed (g/day)	15	20.5
Onion (g/day)	71	142
Orange (g/day)	1	1
Parsnip (g/day)	30	30
Pasta, whole grain (g/day, up to)	150	200
Oatmeal (g/day)	40	50
Salmon spread, 70 % salmon (g/day)	40	80
Squash (g/day)	40	40
Tomato, canned (g/day)	167	229
Iodized salt (g/day)	2	2
Vitamin D pearls (pearls/day)	3	3

Supplementary Table S3. Nutritional analysis of the test products (per 100g).

	Gouda cheese	***Goutaler cheese***	***Pork***	***Beef***
Weight (gram)	100.0	100.0	100.0	100.0
Kilocalories (kcal)	352.1	351.6	238.0	222.4
Kilojoules (kJ)	1473.3	1471.3	996.1	930.9
Fat (gram)	26.9	26.5	18.4	16.1
SFA (gram)[¥]	18.3	18.2	6.2	7.4
Trans fatty acids (gram)[¥]	0.3	0.3	0.0	0.3
MUFA (gram)[¥]	5.4	5.2	8.6	6.4
PUFA (gram)[¥]	0.5	0.5	3.1	0.4
Conjugated linoleic acids (CLA)[¥]	0.1	0.1	0	<0.1
Carbohydrates (gram)	0.0	0.0	0.0	0.0
Protein (gram)	27.5	28.3	18.1	19.4
Calcium (mg)	660.0	746.6	4.7	5.4
Iodine (µg)	22.7	17.3	0.7	2.0
Iron (mg)	0.1	0.1	0.6	2.2
Magnesium (mg)	24.7	29.3	21.0	18.0
Phosphorus (mg)	483.0	516.7	180.0	160.0
Selenium (µg)	11.0	10.7	16.0	6.8
Sodium (mg)	347.0	423.3	57.3	57.7
Vitamin B12 (µg)	1.3	1.2	0.5	1.6

[¥] g/ 100g fatty acid methyl esters (FAME)

Supplementary Table S4. Energy, macro- and micronutrients' intakes at baseline and during the washout periods according to sex.

	Men (n = 10)					*Women (n =23)*				
	**Before period 1**	**Before period 2**	**Before period 3**	**Before period 4**	**p value**	**Before period 1**	**Before period 2**	**Before period 3**	**Before period 4**	**p value**
Energy (kcal)	2,454.7±543.5	2,427.8±530.6	2,657.2±584.8	2,767.9±417.5	0.39	1,751.8±394.9	1,916.6±531.3	1,884.3±498.9	2,030.7±437.2	0.07
Carbohydrates (% TEI)	38.6±7.5	41.7±6.8	40.8±4.7	38.6±8.2	0.44	43.7±5.7	42.2±4.8	41.8±5.1	42±4.9	0.34
Protein (% TEI)	17.8±5.1	18.5±4.9	17.4±4.2	17.4±3.2	0.7	17.5±2.9	17.1±3.4	17±3.1	18±4.5	0.72
Total fat (% TEI)	40.5±6.6	35.8±5	38.3±5.1	41.2±6.5	0.054	35.3±5.3	36.6±5.4	37.1±5.6	36.9±5.7	0.3
SFA (% TEI)	14.7±4.7	12.6±2.8	14±3.4	13.8±3.4	0.43	12.8±4.4	12.5±4	13±3.6	13.6±3.4	0.44
MUFA (% TEI)	15.2±3.4	13.4±2.4	14.3±3	16.3±2.3	0.12	12.6±3	13.2±2.9	14.2±2.6	13.6±2.8	0.12
PUFA (% TEI)	6.5±2.4	6.4±1.1	6.3±1.9	7.3±3.7	0.76	5.7±2.5	7.1±2.5	6.4±1.8	5.9±1.7	0.03
Alcohol (grams)	2.5±5.8	5.4±10.0	5.7±9.1	2.8±5.7	0.68	2.5±4.9	3.3±6.6	3.5±5.5	1.6±3.5	0.32
Added sugar (g/1000kcal)	11.2±7	11.2±8.7	11.2±5	11.1±7.5	0.99	16±10.3	12.6±9	14.5±7.5	11±8.4	0.23
Fiber (g/1000kcal)	12.5±4.8	11.8±2.3	10.6±2.5	10.3±2.2	0.31	13.3±4.9	13.7±3.9	13.5±4.7	13±3.7	0.87
Calcium (mg/1000kcal)	378±145.6	320.1±107.9	441.1±176.7	384.5±154.3	0.01	406.2±146.5	410.7±123	410.6±144.9	421.1±135.7	0.94
Iron (mg/1000kcal)	6.3±2.9	5.4±1	5.1±1	5.2±0.8	0.2	5.5±1.4	5.4±1	5.5±1.6	5.5±0.8	0.94
Sodium (mg/1000kcal)	1401.4±189.6	1316.1±525.2	1478.8±731.8	1760.2±962.2	0.42	1438.5±427.4	1432.8±561.2	1331.3±422.1	1403.7±496.1	0.84
Vitamin B12 (µg/1000kcal)	2.9±1.3	2.6±1	3.1±0.8	3.1±0.8	0.65	2.8±1.6	3.1±1.2	3.1±1.7	3±1.4	0.68

Mixed models for repeated measures

Supplementary Table S5. Comparisons of macro- and micronutrients of the participants' habitual diets with the test diets.

	Habitual intake	*Gouda cheese*	*Goutaler cheese*	*Pork*	*Beef*	*p value RM-ANOVA*
Energy (kcal)	2099 ± 473.6$^{£}$	2624 ± 412.9	2624 ± 412.9	2600 ± 412.9	2565 ± 412.9	<0.01
Carbohydrates (gram)	216.3 ± 47.48$^{£}$	283.2 ± 46.53	283.2 ± 46.53	283.2 ± 46.53	283.2 ± 46.53	<0.01
Carbohydrates (% of TEI)	41.57 ± 4.5$^{£}$	43.15 ± 0.42	43.15 ± 0.42	43.52 ± 0.42	44.14 ± 0.42	<0.01
Protein (gram)	91.02 ± 25.32$^{£}$	103.1 ± 13.72	104.2 ± 13.72	100 ± 13.72	102.7 ± 13.72	<0.01
Protein (% of TEI)	17.39 ± 2.88$^{£}$	15.78 ± 0.36	15.97 ± 0.37	15.43 ± 0.32	16.07 ± 0.37	<0.01
Fat (gram)	88.57 ± 26.4$^{£}$	109.4 ± 16.62	108.8 ± 16.62	107.9 ± 16.62	103 ± 16.62	<0.01
Fat (% of TEI)	37.53 ± 4.37	37.52 ± 0.49	37.33 ± 0.47	37.39 ± 0.50	36.12 ± 0.49$^{£}$	<0.01
SFA (gram)	31.87 ± 11.25$^{£}$	36.66 ± 2.7	36.47 ± 2.7	24.1 ± 2.7$^{\#}$	25.74 ± 2.7$^{\#}$	<0.01
SFA (% TEI)	13.5 ± 3.15	12.72 ± 0.93	12.65 ±0.92	8.40 ± 6.35$^{\#}$	9.45 ± 0.50$^{\#}$	<0.01
Trans FA (g)	0.73 ± 0.55$^{£}$	3.00 ± 0.01	3.00 ± 0.01	0.00 ± 0.01$^{£}$	2 ± 0.01	<0.01
MUFA (gram)	32.78 ± 10.4$^{£}$	44.44 ± 8.21	44.08 ± 8.21	51.17 ± 8.21$^{£}$	48.22 ± 8.21	<0.01

MUFA (%TEI)	13.87 ± 2.08£	15.19 ± 0.50	15.06 ± 0.48	17.71 ± 0.25#	16.90 ± 0.30#	<0.01
PUFA (gram)	15.05 ± 6.25£	21.16 ± 4.22	21.11 ± 4.22	25.73 ± 4.22£	21.04 ± 4.22	<0.01
PUFA (%TEI)	6.33 ± 1.69£	7.22 ± 0.38	7.20 ± 0.38	8.90 ± 0.25£	7.34 ± 0.37	<0.01
Omega-3 (gram)	3.3 ± 1.94£	3.96 ± 0.87	3.96 ± 0.87	4.18 ± 0.87£	3.98 ± 0.87	<0.01
Sugar, added (gram)	27.36 ± 12.11£	31.97 ± 0.51	31.97 ± 0.51	31.97 ± 0.51	31.97 ± 0.51	<0.01
Fiber (gram)	25.91 ± 7.59£	49.25 ± 12.85	49.25 ± 12.85	49.25 ± 12.85	49.25 ± 12.85	<0.01
Beta-carotene (µg)	3145 ± 157.9£	9543 ± 2946	9536 ± 2946	9297 ± 2946£	9426 ± 2946	<0.01
Calcium (mg)	834.6 ± 267.8£	1367 ± 98.89	1498 ± 98.89	362.2 ± 98.89#	389.2 ± 98.89#	<0.01
Copper (mg)	1.35 ± 0.38£	2.31 ± 0.55	2.31 ± 0.55	2.37 ± 0.55	2.25 ± 0.55	<0.01
Folate (µg)	294.4 ± 93.49£	430.4 ± 126.5	421.4 ± 126.5	369.4 ± 126.5#	374.4 ± 126.5#	<0.01
Iodine (µg)	119.9 ± 48.06£	203.4 ± 8.81	194.4 ± 8.81	170.4 ± 8.81#	173.4 ± 8.81#	<0.01
Iron (mg)	11.32 ± 3.27£	15.45 ± 3.39	15.45 ± 3.39	16.65 ± 3.39£	19.85 ± 3.39£	<0.01
Magnesium (mg)	351 ± 87.07£	526.2 ± 103.4	532.2 ± 103.4	532.2 ± 103.4	527.2 ± 103.4	<0.01
Niacin (mg)	20.22 ± 6.67£	24.19 ± 5.67	24.89 ± 5.67	35.19 ± 5.67£	31.99 ± 5.67£	<0.01

Phosphorus (mg)	1662 ± 378.6£	2359 ± 394.5	2415 ± 394.5	2021 ± 394.5£	1977 ± 394.5£	<0.01
Potassium (mg)	3348 ± 672.5£	4512 ± 1349	4503 ± 1349	5058 ± 1349#	4966 ± 1349#	<0.01
Retinol (µg)	582.5 ± 466.1£	625.2 ± 46.02	626 ± 46.02	274.2 ± 46.02£	297.2 ± 46.02£	<0.01
Riboflavin (mg)	1.65 ± 0.46£	1.25 ± 0.19	1.26 ± 0.19	0.99 ± 0.19#	1.03 ± 0.19#	<0.01
Selenium (µg)	60.75 ± 27.1£	71.95 ± 11.6	71.95 ± 11.6	89.95 ± 11.6£	68.95 ± 11.6	<0.01
Sodium (mg)	2946 ± 1031£	3443 ± 246.1	3557 ± 246.1	3055 ± 246.1#	3044 ± 246.1#	<0.01
Thiamin (mg)	1.53 ± 0.4£	1.98 ± 0.36	2.07 ± 0.36	3.03 ± 0.36£	1.95 ± 0.36	<0.01
Vitamin A (RAE)	858.6 ± 530.5£	1421 ± 268.8	1421 ± 268.8	1049 ± 268.8#	1081 ± 268.8#	<0.01
Vitamin B12 (µg)	6.23 ± 2.48£	3.42 ± 0.54	3.32 ± 0.54	2.42 ± 0.54£	4.92 ± 0.54£	<0.01
Vitamin B6 (mg)	1.82 ± 0.58	1.96 ± 0.54	1.95 ± 0.54	1.89 ± 0.54	2.52 ± 0.54£	<0.01
Vitamin C (mg)	106.6 ± 43.12£	172 ± 27.2	172 ± 27.2	172 ± 27.2	172 ± 27.2	<0.01
*Vitamin D (µg)**	6.41 ± 3.34£*	22.16 ± 2.06	22.16 ± 2.06	22.66 ± 2.06	21.75 ± 2.06	<0.01
Vitamin E (alfa-TE)	14.62 ± 5.41£	21.13 ± 4.53	21.43 ± 4.53	21.93 ± 4.53	21.23 ± 4.53	<0.01
Zinc (mg)	11.44 ± 3.39£	17.95 ± 2.46	17.35 ± 2.46	14.75 ± 2.46£	20.25 ± 2.46£	<0.01

Mixed models for repeated measures

£ Different from all other diets

Meat diet different from cheese diets

*After the first intervention, participants were instructed to consume 15µg of vitamin D

Supplementary Table S6. Leisure-time and total physical activity (minutes/week) and sitting time (minutes/day) at baseline and the end of the four intervention periods.

	Baseline	***End of the study***	***p value***
Leisure-time physical activity (min/week)	336.7±358.1	313.9±376.9	0.85
Total physical activity (min/week)	889.3±630.1	618.3±408.8	0.09
Total sitting time (min/day)	561.7±173.3	546.9±158.8	0.63

Paired sample Wilcoxon test

Supplementary Table S7. Effects of the four test diets on clinical markers. Before and after values for each test diet were analyzed with RM-ANOVA adjusted for body weight and period of intervention.

	Gouda			***Goutaler***			***Pork***			***Beef***		
	**pre**	**post**	**p value**	**pre**	**post**	**p value**	**pre**	**post**	**p value**	**pre**	**post**	**p value**
BMI (kg/m2)	25.12±3.33	24.74±3.22	0.02	25.03±3.37	24.67±3.33	<0.01	25.11±3.2	24.87±3.11	0.54	25.14±3.36	24.84±3.31	<0.01
SBP (mmHg)	76.97±8.44	76.86±9.72	0.64	79.45±9.2	76.35±6.46	0.42	77.72±8.21	77.69±7.47	0.53	77.21±7.9	77.54±8.52	0.23
DBP (mmHg)[&]	121.03±9.97	118.21±11.65	0.17	122.52±11.2	117.06±10.51	0.15	123.79±11.66	120.1±9.87	0.15	119.21±9.84	118.79±11.67	0.10
Glucose (mmol/L)	4.58±0.3	4.52±0.24	0.09	4.59±0.24	4.54±0.24	0.20	4.62±0.31	4.57±0.25	0.08	4.62±0.3	4.5±0.29	0.26
Insulin (µUI/mL)	49.41±16.14	46.99±18.74	0.29	52.55±28.08	46.76±18.42	0.11	56.26±17.88	48.63±23.13	0.01	60.44±30.01	54.07±19.11	0.63
ASAT (U/L)	22.31±11.32	20.53±8.88	0.61	20.25±8.77	19.72±7.66	0.10	21.38±9.53	22.06±11.33	0.87	20.13±9.95	19.66±9.49	0.44
ALAT (U/L)	17.5±6.25	15.31±4.55	0.18	16.09±5.19	15.81±6.82	0.40	16.75±6.04	17.19±6.08	0.51	16.69±8.24	16.25±8.47	0.49
Total chol. (mmol/L)	4.58±0.76	4.2±0.63	<.001	4.66±0.8	4.2±0.67	<0.01	4.57±0.76	4.06±0.65	<.001	4.55±0.83	4.11±0.62	<.001
APOB/A ratio	0.56±0.1	0.58±0.11	0.32	0.57±0.11	0.58±0.11	0.28	0.56±0.12	0.55±0.12	0.08	0.57±0.12	0.57±0.12	0.47
CRP (mg/L)	1.13±1.32	0.64±0.57	0.06	1.19±1.86	0.9±1.15	0.39	1.28±1.56	0.76±0.84	0.80	1.25±1.54	0.93±1.32	0.43
Calcium (mmol/L)	2.34±0.09	2.33±0.07	0.06#	2.34±0.09	2.33±0.08	0.29	2.33±0.08	2.34±0.06	0.28	2.33±0.08	2.32±0.07	0.08
Ferritin (µg/L)	66.06±52.48	66.56±55.43	0.76	65.22±56.17	61.41±55.68	0.07#	68.53±57.78	72.41±55.33	0.70	61.88±49.12	66.91±54.35	0.37
Iron (µmol/L)	21.08±9.13	20.54±6.91	0.92	22.44±9.81	19.84±8.46	0.81	22.21±8.81	21.95±8.37	0.36	19.9±7.46	19.13±6.76	0.73
Vitamin B12 (pmol/L)	270.5±99.15	278.58±120.34	0.90	274.42±107.45	274.34±117.51	0.43	271.7±95.42	267.4±114.46	0.49	274.26±127.63	274.65±135.61	0.88
Interleukin 1β (pg/mL)	0.24±0.12	0.26±0.12	0.32	0.24±0.10	0.30±0.23	0.47	0.23±0.08	0.23±0.14	0.98	0.24±0.08	0.25±0.14	0.20
Interleukin-6 (pg/mL) [&]	0.53 ± 0.35	0.56 ± 0.42	0.31	0.64±0.71	0.51±0.33	0.24	0.67 ±0.55	0.59 ± 0.52	0.73	0.54 ± 0.40	0.44±0.29	0.07#

Repeated measures ANOVA: gender and body weight change as covariates and the period of the intervention as a between-subject factor

[&] n = 23

[#] Significant interaction with one or more covariates

Supplementary Figure S1. Vitamin D blood levels before and after test diets according to diet (A) and test period (B). Data were analyzed with repeated-measures ANOVA.

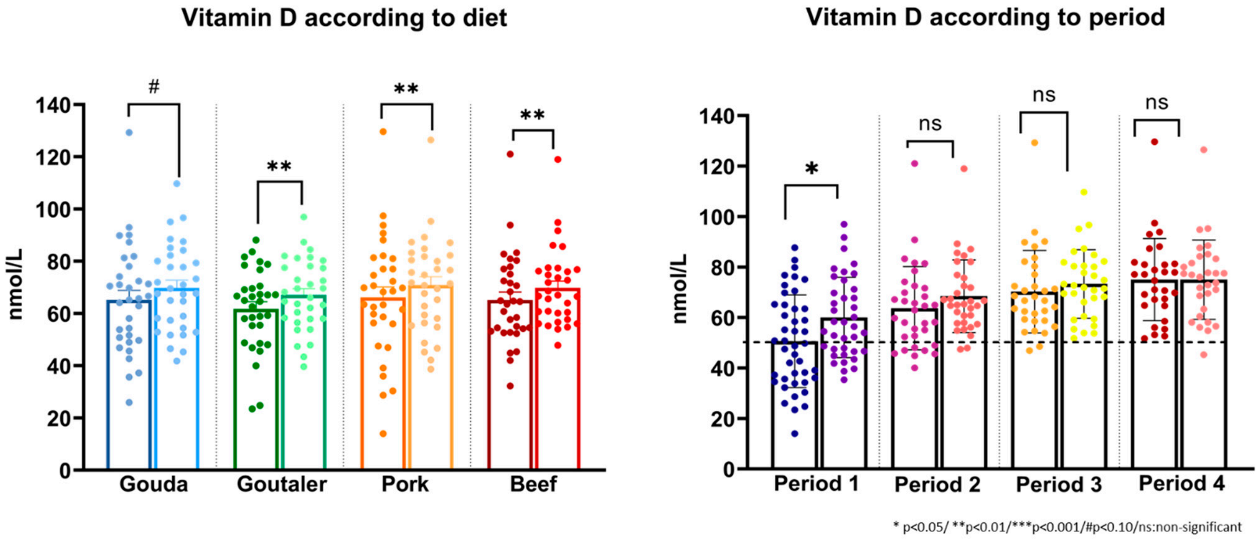

Supplementary Table S8. Description of all lipid species that passed nominal p-value in the Lipidomic analysis for pork diet. Data are represented as median and mean FC (%) ± SD of the percentage of change (post versus pre-intervention values).

Class	Subclass A	Individual notation	Median FC (%)	Mean FC (%)	SD	Nominal p value	FDR-correction p value
Fatty acids	*Saturated fatty acids*	*14:00*	26.8	59.6	96.3	**0.03412**	**0.99854**
Fatty acids	*Saturated fatty acids*	*15:00*	26.6	37.5	59.8	**0.01945**	**0.98216**
Fatty acids	*Saturated fatty acids*	*16:00*	22.2	30.3	41.9	**0.00167**	**0.35407**
Fatty acids	*Saturated fatty acids*	*17:00*	22.7	41.1	62.5	**0.00295**	**0.52472**
Fatty acids	*Monounsaturated fatty acids*	*14:1n-5*	43.5	58.8	90.6	**0.03249**	**0.99805**
Fatty acids	*Monounsaturated fatty acids*	*14:1n-5 trans*	30.1	69.2	103.5	**0.00689**	**0.80051**
Fatty acids	*Monounsaturated fatty acids*	*16:1n-7*	46.7	58.3	81	**0.00002**	**0.00609**
Fatty acids	*Monounsaturated fatty acids*	*16:1n-9*	63.7	92.2	98.5	**0.00001**	**0.00493**
Fatty acids	*Monounsaturated fatty acids*	*17:1n-x*	43.9	68.8	77.2	**0.00011**	**0.03287**
Fatty acids	*Monounsaturated fatty acids*	*18:1n-9*	30.1	45.1	49.4	**0.00003**	**0.01036**
Fatty acids	*Monounsaturated fatty acids*	*19:1n-x*	46.4	61.5	69.8	**0.00008**	**0.02516**
Fatty acids	*Monounsaturated fatty acids*	*20:1n-6*	47.6	63.8	61.1	**0**	**0.00036**
Fatty acids	*Monounsaturated fatty acids*	*24:1n-9*	22.5	25.7	29	**0.00008**	**0.02516**
Fatty acids	*Polyunsaturated fatty acids*	*14:2n-x*	35.3	97.1	108.8	**0.00003**	**0.00936**
Fatty acids	*Polyunsaturated fatty acids*	*16:3n-x*	28	33.4	52.3	**0.00828**	**0.85117**
Fatty acids	*Polyunsaturated fatty acids*	*16:3n-x*	54.5	66.2	91.6	**0.00071**	**0.17873**
Fatty acids	*Polyunsaturated fatty acids*	*18:2n-6*	31.5	41.8	43.5	**0.00001**	**0.00493**
Fatty acids	*Polyunsaturated fatty acids*	*18:2n-x*	34.7	57.2	78.4	**0.00124**	**0.2811**
Fatty acids	*Polyunsaturated fatty acids*	*18:3n-3*	28.5	29.5	39.6	**0.00028**	**0.07863**
Fatty acids	*Polyunsaturated fatty acids*	*18:3n-6*	35.2	44.9	59.4	**0.00223**	**0.43741**
Fatty acids	*Polyunsaturated fatty acids*	*20:2n-6*	42.8	68	64.3	**0**	**0.00008**
Fatty acids	*Polyunsaturated fatty acids*	*20:2n-x*	45.8	51.4	61.1	**0.0004**	**0.10794**
Fatty acids	*Polyunsaturated fatty acids*	*20:3n-3*	27.4	31	35.3	**0.0001**	**0.03023**
Fatty acids	*Polyunsaturated fatty acids*	*20:3n-9*	36	51	59.6	**0.00013**	**0.03937**
Fatty acids	*Polyunsaturated fatty acids*	*20:3n-x*	29.7	35	38.5	**0.00006**	**0.01882**
Fatty acids	*Polyunsaturated fatty acids*	*20:4n-3*	31	44.3	51.5	**0.00066**	**0.16821**
Fatty acids	*Polyunsaturated fatty acids*	*20:4n-6*	16.4	27.2	33.7	**0.00003**	**0.00936**
Fatty acids	*Polyunsaturated fatty acids*	*20:4n-x*	29	39.8	63.3	**0.04134**	**0.99954**
Fatty acids	*Polyunsaturated fatty acids*	*22:3n-x*	19.8	31.7	50	**0.00536**	**0.72316**
Fatty acids	*Polyunsaturated fatty acids*	*22:3n-x*	31.5	50.7	60.2	**0.00006**	**0.01882**
Fatty acids	*Polyunsaturated fatty acids*	*22:4n-6*	36.9	47.5	50.5	**0.00001**	**0.00347**
Fatty acids	*Polyunsaturated fatty acids*	*22:5n-3*	15.4	20.8	36.3	**0.0118**	**0.92574**
Oxidized fatty acids	*Hydroxy-octadecadienoic acids*	*x-HODE*	31.3	65	75.6	**0.00004**	**0.01402**

Oxidized fatty acids	*Hydroxy-octadecadienoic acids*	*x-HODE*	22.9	36.4	65.7	**0.00124**	**0.2811**
Fatty esters	*Acylcarnitines*	*AC(8:0)*	20.3	36.7	65.9	**0.02279**	**0.99071**
Fatty esters	*Acylcarnitines*	*AC(10:0)*	21.7	36.6	62.3	**0.01746**	**0.97391**
Fatty esters	*Acylcarnitines*	*AC(10:1n-x)*	25.1	39.9	59.1	**0.00362**	**0.5971**
Fatty esters	*Acylcarnitines*	*AC(14:1n-x)*	36.8	64.1	98.3	**0.00536**	**0.72316**
Fatty esters	*Acylcarnitines*	*AC(14:2n-x)*	42.3	68.9	98.8	**0.00084**	**0.20372**
Fatty esters	*Acylcarnitines*	*AC(16:0)*	14.9	18.7	35	**0.00471**	**0.681**
Fatty esters	*Acylcarnitines*	*AC(18:1n-9)*	33.4	31.8	27.5	**0**	**0.00055**
Fatty amides	*N-acyl ethanolamines*	*NAE(18:1n-9)*	10.1	21.7	40.4	**0.00239**	**0.45944**
Glycerolipids	*Diacylglycerols*	*DG(34:1)*	-24.6	-14.3	40.4	**0.00256**	**0.48051**
Glycerolipids	*Diacylglycerols*	*DG(36:2)*	-21.4	-12.8	40.3	**0.00387**	**0.61731**
Glycerolipids	*Triacylglycerols*	*TG(42:1)*	-63.9	-55.9	39.1	**0**	**0.00004**
Glycerolipids	*Triacylglycerols*	*TG(44:1)*	-64.1	-58.4	29.2	**0**	**0**
Glycerolipids	*Triacylglycerols*	*TG(45:0)*	-3	-7.8	16.6	**0.04977**	**0.99989**
Glycerolipids	*Triacylglycerols*	*TG(45:1)*	-23.1	-28	28.9	**0**	**0.00048**
Glycerolipids	*Triacylglycerols*	*TG(46:0)*	-16.8	-29	29.7	**0**	**0.00014**
Glycerolipids	*Triacylglycerols*	*TG(46:1)*	-64.7	-61.3	27.7	**0**	**0**
Glycerolipids	*Triacylglycerols*	*TG(46:2)*	-58.4	-59.2	25.1	**0**	**0**
Glycerolipids	*Triacylglycerols*	*TG(46:3)*	-54.3	-56.5	25.2	**0**	**0**
Glycerolipids	*Triacylglycerols*	*TG(46:3)*	-56.8	-57.9	24.6	**0**	**0**
Glycerolipids	*Triacylglycerols*	*TG(47:1)*	-30.7	-36.1	30.6	**0**	**0.00003**
Glycerolipids	*Triacylglycerols*	*TG(47:2)*	-33.3	-35.9	27.8	**0**	**0.00002**
Glycerolipids	*Triacylglycerols*	*TG(48:0)*	-20.8	-28.7	32.3	**0.00002**	**0.00548**
Glycerolipids	*Triacylglycerols*	*TG(48:1)*	-54.7	-48.5	40.9	**0**	**0.00002**
Glycerolipids	*Triacylglycerols*	*TG(48:2)*	-56.3	-52.3	33.3	**0**	**0.00001**
Glycerolipids	*Triacylglycerols*	*TG(48:3)*	-55.4	-54.1	25.5	**0**	**0**
Glycerolipids	*Triacylglycerols*	*TG(48:4)*	-64.6	-59.8	21.4	**0**	**0**
Glycerolipids	*Triacylglycerols*	*TG(49:0)*	-12	-18	24.3	**0.00008**	**0.02516**
Glycerolipids	*Triacylglycerols*	*TG(49:1)*	-51.2	-40.6	42.5	**0**	**0.00014**
Glycerolipids	*Triacylglycerols*	*TG(49:2)*	-43	-33.4	41	**0**	**0.00096**
Glycerolipids	*Triacylglycerols*	*TG(49:3)*	-42.4	-34.6	35.9	**0**	**0.00084**
Glycerolipids	*Triacylglycerols*	*TG(50:0)*	-25.5	-30.5	39	**0.00006**	**0.01882**
Glycerolipids	*Triacylglycerols*	*TG(50:1)*	-31.4	-12	74.6	**0.00066**	**0.16821**
Glycerolipids	*Triacylglycerols*	*TG(50:2)*	-35.7	-19.5	57	**0.0001**	**0.03023**
Glycerolipids	*Triacylglycerols*	*TG(50:3)*	-36.9	-23	52.1	**0.00005**	**0.01702**
Glycerolipids	*Triacylglycerols*	*TG(50:4)*	-37.7	-30.6	40.3	**0.00001**	**0.00273**
Glycerolipids	*Triacylglycerols*	*TG(50:5)*	-37.5	-37.4	25.4	**0**	**0.00001**

Glycerolipids	*Triacylglycerols*	*TG(51:1)*	-49.4	-31.6	53.7	**0.00001**	**0.00212**
Glycerolipids	*Triacylglycerols*	*TG(51:2)*	-43.6	-27.6	52.8	**0.00002**	**0.00548**
Glycerolipids	*Triacylglycerols*	*TG(51:3)*	-35.4	-18.7	48.9	**0.00066**	**0.16821**
Glycerolipids	*Triacylglycerols*	*TG(51:4)*	-29.3	-17.6	47.2	**0.00066**	**0.16821**
Glycerolipids	*Triacylglycerols*	*TG(52:0)*	-15	-22.7	39.2	**0.00133**	**0.29675**
Glycerolipids	*Triacylglycerols*	*TG(52:1)*	-45.6	-23.1	59.4	**0.00016**	**0.0471**
Glycerolipids	*Triacylglycerols*	*TG(52:2)*	-25.2	-9.2	57.7	**0.00387**	**0.61731**
Glycerolipids	*Triacylglycerols*	*TG(52:5)*	-19.6	-10.8	42.1	**0.0118**	**0.92574**
Glycerolipids	*Triacylglycerols*	*TG(52:6)*	-28.6	-26.5	38.6	**0.00004**	**0.01402**
Glycerolipids	*Triacylglycerols*	*TG(53:1)*	-43.1	-31	50	**0.00002**	**0.0068**
Glycerolipids	*Triacylglycerols*	*TG(53:2)*	-36.2	-23.9	50.3	**0.00004**	**0.01145**
Glycerolipids	*Triacylglycerols*	*TG(53:3)*	-30.7	-16.7	46.2	**0.00114**	**0.26509**
Glycerolipids	*Triacylglycerols*	*TG(53:4)*	-18.6	-8.5	42.8	**0.03092**	**0.9976**
Glycerolipids	*Triacylglycerols*	*TG(54:1)*	-43.5	-30.7	53	**0.00008**	**0.02516**
Glycerolipids	*Triacylglycerols*	*TG(54:2)*	-33.1	-24.7	40	**0.0001**	**0.03023**
Glycerolipids	*Triacylglycerols*	*TG(54:3)*	-23.5	-11.1	40.4	**0.02659**	**0.99532**
Glycerolipids	*Triacylglycerols*	*TG(55:2)*	-31.8	-28.1	38.1	**0.00001**	**0.00493**
Glycerolipids	*Triacylglycerols*	*TG(55:3)*	-27.3	-20.1	38.1	**0.00061**	**0.15658**
Glycerolipids	*Triacylglycerols*	*TG(55:4)*	-18.6	-13.6	30.4	**0.0088**	**0.86428**
Glycerolipids	*Triacylglycerols*	*TG(56:1)*	-38.9	-35.5	35.6	**0**	**0.00036**
Glycerolipids	*Triacylglycerols*	*TG(56:2)*	-35.5	-34.4	32.5	**0**	**0.00055**
Glycerolipids	*Triacylglycerols*	*TG(56:3)*	-26.4	-16.5	35.7	**0.00441**	**0.66006**
Glycerolipids	*Triacylglycerols*	*TG(56:6)*	-26.1	-10.3	37.8	**0.01051**	**0.90419**
Glycerolipids	*Triacylglycerols*	*TG(56:7)*	21.7	41.5	58.5	**0.00413**	**0.63881**
Glycerolipids	*Triacylglycerols*	*TG(56:7)*	-43.8	-28.3	41	**0.0001**	**0.03023**
Glycerolipids	*Triacylglycerols*	*TG(56:8)*	-37.1	-21.1	46.6	**0.0004**	**0.10794**
Glycerolipids	*Triacylglycerols*	*TG(58:1)*	-39.7	-38.1	33.6	**0**	**0.00016**
Glycerolipids	*Triacylglycerols*	*TG(58:2)*	-48.5	-44.5	37.8	**0**	**0.0001**
Glycerolipids	*Triacylglycerols*	*TG(58:3)*	-35.8	-31	38.4	**0.00002**	**0.00757**
Glycerolipids	*Triacylglycerols*	*TG(58:4)*	-18.9	-13.3	44.1	**0.01114**	**0.91495**
Glycerolipids	*Triacylglycerols*	*TG(58:5)*	-33.2	-24	36.2	**0.00001**	**0.0044**
Glycerolipids	*Triacylglycerols*	*TG(58:8)*	-25.2	-19.6	39.9	**0.0004**	**0.10794**
Glycerolipids	*Triacylglycerols*	*TG(58:9)*	-23.9	-11	45.6	**0.00828**	**0.85117**
Glycerolipids	*Triacylglycerols*	*TG(60:1)*	-46.6	-35.4	40.7	**0**	**0.00084**
Glycerolipids	*Triacylglycerols*	*TG(60:2)*	-47	-43.9	27.4	**0**	**0.00002**
Glycerolipids	*Triacylglycerols*	*TG(60:3)*	-38.4	-29	38.5	**0.00003**	**0.0084**
Sterols	*Cholesteryl Ester*	*ChoE(18:3)*	-34.3	-26.8	29.4	**0**	**0.00074**

Sterols	*Cholesteryl Ester*	*ChoE(20:3)*	-27.4	-18.4	28.9	**0.00007**	**0.02064**
Sterols	*Cholesteryl Ester*	*ChoE(20:5)*	-41.9	-27.5	54.4	**0.00001**	**0.0044**
Bile acids	*Taurine-conjugated bile acids*	*Taurodeoxycholic acid*	79.5	109.3	198.4	**0.00733**	**0.81736**
Bile acids	*Taurine-conjugated bile acids*	*Taurochenodeoxycholic acid*	32	112.4	185.8	**0.00608**	**0.76317**
Bile acids	*Taurine-conjugated bile acids*	*Taurolithocholic acid*	-46.7	-10.5	102.6	**0.0148**	**0.95637**
Sterols	*Steroid sulfates*	*androsterone sulfate + etiocholanolone sulfate*	12.8	16.4	38.7	**0.03092**	**0.9976**
Sterols	*Steroid sulfates*	*isomer androsterone sulfate*	14	*28.2*	60.7	**0.04977**	**0.99989**
Sterols	*Steroid sulfates*	*isomer androsterone sulfate*	7.5	*19.3*	35.6	**0.024**	**0.99261**
Sterols	*Steroid sulfates*	*isomer Dehydroepiandrosterone sulfate (DHEAS)*	12.8	*16*	26.2	**0.014**	**0.94964**
Glycerophospholipids	*PE*	*PE(36:3)*	-24.2	*-11.3*	35.9	**0.01574**	**0.96314**
Glycerophospholipids	*ether-PE*	*PE(18:2e/22:6)*	50.7	*67*	80.5	**0**	**0.00006**
Glycerophospholipids	*P-PE*	*PE(P-16:0/20:4)*	28.2	*38.1*	69.6	**0.024**	**0.99261**
Glycerophospholipids	*P-PE*	*PE(P-18:1/20:4)*	119	117.4	103.2	**0**	**0.00006**
Glycerophospholipids	*LPE*	*PE(14:0/0:0)*	-24.4	-24.5	21.8	**0**	**0.00036**
Glycerophospholipids	*LPE*	*PE(18:1/0:0)*	-7.8	-7.6	18.8	**0.02798**	**0.99616**
Glycerophospholipids	*LPE*	*PE(18:1/0:0)*	-45.3	-28.2	60.5	**0.00001**	**0.00391**
Glycerophospholipids	*LPE*	*PE(18:2/0:0)*	-10.2	-10.1	21.9	**0.01051**	**0.90419**
Glycerophospholipids	*LPE*	*PE(18:2/0:0)*	-43.1	-35.7	33.8	**0**	**0.0003**
Glycerophospholipids	*LPE*	*PE(18:3/0:0)*	-46.3	-31.7	55.1	**0.00002**	**0.0068**
Glycerophospholipids	*LPE*	*PE(20:3/0:0)*	-24.1	-20.2	28.2	**0.00005**	**0.01543**
Glycerophospholipids	*LPE*	*LPE(20:5)*	-34.8	-22.1	38	**0.00018**	**0.05144**
Glycerophospholipids	*LPE*	*PE(20:5/0:0)*	-33.7	-28.5	34.7	**0**	**0.00145**
Glycerophospholipids	*LPE*	*PE(22:5/0:0)*	-9.3	-2.3	.	**0.03558**	**0.99886**
Glycerophospholipids	*LPE*	*PE(22:5/0:0)*	-20.3	-11.9	35.2	**0.00608**	**0.76317**
Glycerophospholipids	*LPE*	*PE(0:0/18:1)*	-16.1	-13.9	26.7	**0.00648**	**0.7815**
Glycerophospholipids	*LPE*	*PE(0:0/18:2)*	-5.5	-8.3	22.6	**0.04977**	**0.99989**
Glycerophospholipids	*LPE*	*PE(0:0/20:3)*	-19.1	-16.3	34.7	**0.0002**	**0.05611**
Glycerophospholipids	*LPE*	*PE(0:0/20:4)*	13.1	12.6	22.7	**0.01323**	**0.94217**
Glycerophospholipids	*O-LPE*	*PE(O-16:0/0:0)*	19.6	48.8	82.4	**0.014**	**0.94964**
Glycerophospholipids	*P-LPE*	*PE(P-18:1/0:0)*	43.6	68.1	81.3	**0.00002**	**0.00757**
Glycerophospholipids	*P-LPE*	*PE(P-18:1/0:0)*	-24.7	-12.1	49.7	**0.0118**	**0.92574**
Glycerophospholipids	*P-LPE*	*PE(P-18:2/0:0)*	86.2	124.5	131	**0**	**0.00008**
Glycerophospholipids	*P-LPE*	*PE(P-20:2/0:0)*	35.9	61.9	114.5	**0.04977**	**0.99989**
Glycerophospholipids	*PC*	*PC(28:0)*	-84.4	-77.1	18.5	**0**	**0**
Glycerophospholipids	*PC*	*PC(30:0)*	-68.4	-58.6	27.7	**0**	**0**
Glycerophospholipids	*PC*	*PC(30:1)*	-65.8	-60.1	25.2	**0**	**0**

Glycerophospholipids	*PC*	*PC(31:0)*	-26.8	-20.1	32.1	**0.00002**	**0.00609**
Glycerophospholipids	*PC*	*PC(32:1)*	-35.5	-23.1	41.9	**0.00003**	**0.01036**
Glycerophospholipids	*PC*	*PC(32:2)*	-52.3	-46	25.1	**0**	**0**
Glycerophospholipids	*PC*	*PC(33:0)*	-53.2	-40.7	37.5	**0**	**0.00026**
Glycerophospholipids	*PC*	*PC(33:1)*	-39.2	-31.5	34.9	**0**	**0.00074**
Glycerophospholipids	*PC*	*PC(33:2)*	-36.5	-22.1	39.9	**0.00003**	**0.00936**
Glycerophospholipids	*PC*	*PC(34:1)*	-21	-11.2	37.2	**0.00239**	**0.45944**
Glycerophospholipids	*PC*	*PC(34:3)*	37.2	49.5	86.6	**0.04754**	**0.99984**
Glycerophospholipids	*PC*	*PC(34:3)*	-56	-41.2	49.2	**0**	**0.00022**
Glycerophospholipids	*PC*	*PC(34:4)*	-55.4	-48.2	27	**0**	**0**
Glycerophospholipids	*PC*	*PC(35:1)*	-27.9	-17.6	39.8	**0.00013**	**0.03937**
Glycerophospholipids	*PC*	*PC(35:2)*	-19.1	-5.9	41.5	**0.0125**	**0.93391**
Glycerophospholipids	*PC*	*PC(35:4)*	-26.3	-15.9	36.7	**0.00028**	**0.07863**
Glycerophospholipids	*PC*	*PC(36:1)*	-27.5	-18.3	37.5	**0.00033**	**0.09217**
Glycerophospholipids	*PC*	*PC(36:3)*	-21.3	-14.2	36	**0.00051**	**0.13521**
Glycerophospholipids	*PC*	*PC(36:5)*	-41.8	-27.7	51.4	**0**	**0.00074**
Glycerophospholipids	*PC*	*PC(36:5)*	-41.7	-27.6	51.4	**0**	**0.00074**
Glycerophospholipids	*PC*	*PC(36:6)*	-56.6	-50.1	23.2	**0**	**0**
Glycerophospholipids	*PC*	*PC(37:2)*	-26.2	-14.9	34	**0.00256**	**0.48051**
Glycerophospholipids	*PC*	*PC(37:3)*	-30.1	-18	50.2	**0.00012**	**0.03605**
Glycerophospholipids	*PC*	*PC(37:5)*	-56.2	-30.3	78.3	**0**	**0.00164**
Glycerophospholipids	*PC*	*PC(37:6)*	-25.5	-23.8	30.3	**0.00001**	**0.00212**
Glycerophospholipids	*PC*	*PC(38:2)*	-8.5	-3.9	37.8	**0.02942**	**0.99695**
Glycerophospholipids	*PC*	*PC(38:3)*	-31.7	-21	41.2	**0.00012**	**0.03605**
Glycerophospholipids	*PC*	*PC(38:5)*	-42.1	-29.9	56.6	**0**	**0.00145**
Glycerophospholipids	*PC*	*PC(38:6)*	-18.6	-5.7	50.2	**0.00689**	**0.80051**
Glycerophospholipids	*PC*	*PC(40:1)*	-26.6	-19.6	45	**0.00056**	**0.14559**
Glycerophospholipids	*PC*	*PC(40:4)*	-12.8	-6.1	50.5	**0.02051**	**0.98542**
Glycerophospholipids	*PC*	*PC(40:5)*	-34	-26.2	40	**0.00004**	**0.01264**
Glycerophospholipids	*PC*	*PC(40:7)*	-37.6	-20	44.9	**0.00031**	**0.08504**
Glycerophospholipids	*ether-PC*	*PC(18:1e/20:3)*	-28.5	-10.6	64.5	**0.00114**	**0.26509**
Glycerophospholipids	*O-PC*	*PC(O-16:0/14:0)*	-39.6	-31.6	34.8	**0**	**0.00008**
Glycerophospholipids	*O-PC*	*PC(O-16:0/18:2)*	94.1	160.2	158.6	**0**	**0.00005**
Glycerophospholipids	*O-PC*	*PC(O-16:0/20:4)*	69.6	83.7	113.6	**0.00001**	**0.00307**
Glycerophospholipids	*O-PC*	*PC(O-18:1/18:2)*	95.3	123.6	97.2	**0**	**0**
Glycerophospholipids	*O-PC*	*PC(O-18:1/22:4)*	23.7	35.5	52.3	**0.00004**	**0.01145**
Glycerophospholipids	*O-PC*	*PC(O-38:4)*	39.8	57	74.9	**0.00001**	**0.00273**

Glycerophospholipids	*O-PC*	*PC(O-38:5)*	37.3	58.8	78.5	**0**	**0.00164**
Glycerophospholipids	*P-PC*	*PC(P-16:0/14:0)*	-36.8	-32.5	24.6	**0**	**0.00001**
Glycerophospholipids	*P-PC*	*PC(P-16:0/18:1)*	-9.1	0.3	42.4	**0.03582**	**0.99887**
Glycerophospholipids	*P-PC*	*PC(P-17:0/20:4)*	-21.1	-5.3	55.6	**0.00441**	**0.66006**
Glycerophospholipids	*P-PC*	*PC(P-18:0/20:4)*	20.2	57.4	130.4	**0.00043**	**0.11585**
Glycerophospholipids	*LPC*	*PC(14:0/0:0)*	-47.7	-45.2	16.5	**0**	**0**
Glycerophospholipids	*LPC*	*PC(15:0/0:0)*	-24.6	-22	19.3	**0**	**0.00048**
Glycerophospholipids	*LPC*	*PC(16:1/0:0)*	-3.3	-4.8	34.3	**0.0454**	**0.99977**
Glycerophospholipids	*LPC*	*PC(16:1/0:0)*	-27.6	-22	28.6	**0.00002**	**0.00548**
Glycerophospholipids	*LPC*	*PC(17:1/0:0)*	-20	-11.3	33.2	**0.00275**	**0.50182**
Glycerophospholipids	*LPC*	*PC(18:1/0:0)*	-55.7	-44.5	45	**0**	**0.00014**
Glycerophospholipids	*LPC*	*PC(18:2/0:0)*	-22.9	-17.5	37.7	**0.00133**	**0.29675**
Glycerophospholipids	*LPC*	*PC(18:3/0:0)*	-35.1	-32.2	29.4	**0**	**0.00011**
Glycerophospholipids	*LPC*	*PC(20:1/0:0)*	-41.1	-27.2	49	**0.00002**	**0.00548**
Glycerophospholipids	*LPC*	*PC(20:2/0:0)*	-50.9	-37	35.3	**0**	**0.00074**
Glycerophospholipids	*LPC*	*PC(20:3/0:0)*	-19.2	-13.1	34.6	**0.00362**	**0.5971**
Glycerophospholipids	*LPC*	*PC(20:4/0:0)*	13.5	11.4	23.7	**0.03943**	**0.99941**
Glycerophospholipids	*LPC*	*PC(20:5/0:0)*	-26.1	-18.6	42.1	**0.00021**	**0.06115**
Glycerophospholipids	*LPC*	*PC(22:5/0:0)*	-23.5	-16.8	33.2	**0.00124**	**0.2811**
Glycerophospholipids	*LPC*	*PC(0:0/14:0)*	-49.9	-49.7	14.4	**0**	**0**
Glycerophospholipids	*LPC*	*PC(0:0/15:0)*	-31.4	-27.6	18.9	**0**	**0.00001**
Glycerophospholipids	*LPC*	*PC(0:0/17:0)*	-10.6	-7.3	26.9	**0.02527**	**0.99402**
Glycerophospholipids	*LPC*	*PC(0:0/17:1)*	-17.1	-7.3	36.1	**0.01323**	**0.94217**
Glycerophospholipids	*LPC*	*PC(0:0/18:1)*	-4	-5.3	18.1	**0.02659**	**0.99532**
Glycerophospholipids	*LPC*	*PC(0:0/18:3)*	-24.3	-14.3	47.2	**0.00413**	**0.63881**
Glycerophospholipids	*LPC*	*PC(0:0/20:3)*	-18	-16.8	22.5	**0.00003**	**0.01036**
Glycerophospholipids	*LPC*	*PC(0:0/20:5)*	-26	-18.1	41.2	**0.00071**	**0.17873**
Glycerophospholipids	*LPC*	*PC(0:0/22:5)*	-25.8	-19.8	35.3	**0.00071**	**0.17873**
Glycerophospholipids	*LPC*	*LPC(22:0)*	-11.4	-7.1	34.5	**0.02942**	**0.99695**
Glycerophospholipids	*O-LPC*	*PC(O-16:0/0:0)*	37.6	40.8	36.6	**0**	**0.00008**
Glycerophospholipids	*O-LPC*	*PC(O-18:1/0:0)*	25.8	28.5	30.2	**0.00002**	**0.00757**
Glycerophospholipids	*O-LPC*	*PC(O-18:1/0:0)*	-25.7	-21.7	36.8	**0.00004**	**0.01145**
Glycerophospholipids	*O-LPC*	*PC(O-20:2/0:0)*	34.1	43.2	62.8	**0.0078**	**0.83472**
Glycerophospholipids	*O-LPC*	*LPC(O-22:2)*	19.3	30.7	47.6	**0.00934**	**0.87778**
Glycerophospholipids	*O-LPC*	*PC(O-24:2/0:0)*	16.9	*20*	*38.2*	*0.04134*	**0.99954**
Glycerophospholipids	*P-LPC*	*PC(P-16:0/0:0)*	10.3	*12*	*25.9*	*0.03943*	**0.99941**
Glycerophospholipids	*P-LPC*	*PC(P-18:1/0:0)*	28.8	*37.9*	*51.6*	*0.00207*	**0.41702**

Glycerophospholipids	*P-LPC*	*PC(P-18:1/0:0)*	-14.7	*-3.3*	*50.4*	*0.03092*	**0.9976**
Glycerophospholipids	*P-LPC*	*PC(P-20:2/0:0)*	33.6	*70.3*	*120.6*	*0.02798*	**0.99616**
Glycerophospholipids	*PI*	*PI(36:4)*	-29.1	*-13.5*	*68.5*	*0.00021*	**0.06115**
Glycerophospholipids	*LPI*	*LPI(16:0)*	-22.1	*-20.1*	*35.8*	*0.00084*	**0.20372**
Glycerophospholipids	*LPI*	*LPI(16:0)*	-25.7	*-17*	*39.4*	*0.00091*	**0.21711**
Glycerophospholipids	*LPI*	*LPI(18:1)*	-14.5	*-12.5*	*29.4*	*0.00536*	**0.72316**
Glycerophospholipids	*LPI*	*LPI(18:1)*	-16.2	*-12.6*	*28.8*	*0.00828*	**0.85117**
Glycerophospholipids	*LPI*	*LPI(22:6)*	-17.6	-11.5	36.6	**0.00934**	**0.87778**
Sphingolipids	*Ceramides*	*Cer(d18:1/20:0)*	-17.2	-8.3	40.2	**0.01746**	**0.97391**
Sphingolipids	*Ceramides*	*Cer(d18:1/22:0)*	-25.6	-19.7	34.8	**0.00005**	**0.01543**
Sphingolipids	*Ceramides*	*Cer(40:2)*	-9.7	-8.3	18.9	**0.0125**	**0.93391**
Sphingolipids	*Ceramides*	*Cer(d18:1/23:0)*	-23.5	-15.1	44.5	**0.00036**	**0.0998**
Sphingolipids	*Ceramides*	*Cer(d18:1/24:0)*	-26.3	-21.6	36.5	**0.00001**	**0.00493**
Sphingolipids	*Ceramides*	*Cer(d18:1/25:0)*	-15.1	-9.2	41.2	**0.00207**	**0.41702**
Sphingolipids	*Sphingomyelin*	*SM(31:1)*	-41.4	-34.8	22	**0**	**0.00001**
Sphingolipids	*Sphingomyelin*	*SM(38:0)*	-13.3	-3	43.9	**0.02659**	**0.99532**
Sphingolipids	*Sphingomyelin*	*SM(42:1)*	-14	-8.8	36.5	**0.00077**	**0.19031**
Sphingolipids	*Sphingomyelin*	*SM(32:1)*	-30.1	-23.3	23.1	**0**	**0.00022**
Sphingolipids	*Sphingomyelin*	*SM(33:1)*	-15.4	-5.6	29.4	**0.0148**	**0.95637**
Sphingolipids	*Sphingomyelin*	*SM(d18:0/14:0)*	-46.5	-39.8	20.6	**0**	**0**
Sphingolipids	*Sphingomyelin*	*SM(d18:0/18:0)*	19.8	38.3	80	**0.00471**	**0.681**
Sphingolipids	*Sphingomyelin*	*SM(d18:1/12:0)*	-48.4	-47.3	14.2	**0**	**0**
Sphingolipids	*Sphingomyelin*	*SM(36:2)*	5.3	21.4	47.1	**0.03943**	**0.99941**
Sphingolipids	*Sphingomyelin*	*SM(38:1)*	-14.5	-6.2	34.4	**0.00179**	**0.3741**
Sphingolipids	*Sphingomyelin*	*SM(39:1)*	-24	-14.6	34	**0.00004**	**0.01402**
Sphingolipids	*Sphingomyelin*	*SM(d18:1/22:0)*	-13.5	-8.5	37.5	**0.00026**	**0.07238**
Sphingolipids	*Sphingomyelin*	*SM(d18:1/23:0)*	-15	-6.2	41.1	**0.00362**	**0.5971**
Sphingolipids	*Sphingomyelin*	*SM(d18:2/14:0)*	-37.5	-31.6	20.6	**0**	**0.00002**
Sphingolipids	*Sphingomyelin*	*SM(d18:2/22:0)*	-9.6	-3.9	39.2	**0.00503**	**0.70156**
Sphingolipids	*Monohexosylceramides*	*CMH(d18:1/22:0)*	-14.6	4.6	71	**0.0088**	**0.86428**

Supplementary Table S9. Description of all lipid species that passed nominal p-value in the Lipidomic analysis for beef test diet. Data are represented as median and mean FC (%) ± SD of the percentage of change (post versus pre-intervention values).

Class	Subclass A	Individual notation	Median FC (%)	Mean FC (%)	SD	Nominal p value	FDR-correction p value
Fatty acids	*Saturated fatty acids*	*15:00*	39.6	57.5	109.2	**0.0118**	**0.94674**
Fatty acids	*Saturated fatty acids*	*17:00*	34.5	62.5	107.1	**0.00471**	**0.71518**
Fatty acids	*Monounsaturated fatty acids*	*14:1n-5*	52.9	73.3	142	**0.0454**	**0.99991**
Fatty acids	*Monounsaturated fatty acids*	*16:1n-7*	47.8	55.8	70.2	**0.00167**	**0.3907**
Fatty acids	*Monounsaturated fatty acids*	*16:1n-9*	32.4	97.5	173.1	**0.00295**	**0.56628**
Fatty acids	*Monounsaturated fatty acids*	*17:1n-x*	57.6	90.3	149.8	**0.00275**	**0.54385**
Fatty acids	*Monounsaturated fatty acids*	*18:1n-9*	36.8	44	75.9	**0.01114**	**0.93854**
Fatty acids	*Monounsaturated fatty acids*	*19:1n-x*	45.1	67.4	112	**0.00471**	**0.71518**
Fatty acids	*Polyunsaturated fatty acids*	*18:2n-6*	14.4	35	65.9	**0.01945**	**0.9897**
Fatty acids	*Polyunsaturated fatty acids*	*18:2n-x*	21.8	60.3	115.6	**0.03092**	**0.9989**
Fatty acids	*Polyunsaturated fatty acids*	*18:3n-3*	22.7	33	54.2	**0.00536**	**0.75534**
Oxidized fatty acids	*Dihydroxy-octadecadenoic acids*	*12.13-DiHOME*	-16.5	-5.9	45.2	**0.01746**	**0.98541**
Glycerolipids	*Triacylglycerols*	*TG(42:1)*	-46.8	-13.8	206	**0**	**0.00178**
Glycerolipids	*Triacylglycerols*	*TG(44:1)*	-49.9	-37	80.2	**0**	**0.00038**
Glycerolipids	*Triacylglycerols*	*TG(45:1)*	-21.1	-19.9	26.9	**0.00012**	**0.04108**
Glycerolipids	*Triacylglycerols*	*TG(46:0)*	-17.4	-20.7	26	**0.00061**	**0.17428**
Glycerolipids	*Triacylglycerols*	*TG(46:1)*	-53.5	-35.1	93.2	**0**	**0.00038**
Glycerolipids	*Triacylglycerols*	*TG(46:2)*	-49.5	-35.6	78.2	**0**	**0.00028**
Glycerolipids	*Triacylglycerols*	*TG(46:3)*	-49.3	-33	92.3	**0**	**0.0002**
Glycerolipids	*Triacylglycerols*	*TG(46:3)*	-53.7	-34.7	92.5	**0**	**0.00038**
Glycerolipids	*Triacylglycerols*	*TG(47:1)*	-19.8	-21.7	31.1	**0.00018**	**0.05799**
Glycerolipids	*Triacylglycerols*	*TG(47:2)*	-17.2	-19.8	30.7	**0.0002**	**0.06327**
Glycerolipids	*Triacylglycerols*	*TG(48:0)*	-25.2	-20.3	29.6	**0.00077**	**0.21193**
Glycerolipids	*Triacylglycerols*	*TG(48:1)*	-43.8	-27.1	70.3	**0.00002**	**0.00816**
Glycerolipids	*Triacylglycerols*	*TG(48:2)*	-39.7	-25.7	75.5	**0.00002**	**0.00657**
Glycerolipids	*Triacylglycerols*	*TG(48:3)*	-43.3	-27.9	78.9	**0**	**0.00178**
Glycerolipids	*Triacylglycerols*	*TG(48:4)*	-44.3	-5.7	219.8	**0**	**0.00136**
Glycerolipids	*Triacylglycerols*	*TG(49:1)*	-13.9	-5.9	58	**0.01746**	**0.98541**
Glycerolipids	*Triacylglycerols*	*TG(49:2)*	-16	-9.4	46.5	**0.0088**	**0.89496**
Glycerolipids	*Triacylglycerols*	*TG(49:3)*	-20.3	-13.3	37.8	**0.00441**	**0.69429**
Glycerolipids	*Triacylglycerols*	*TG(50:0)*	-19.8	-15.6	40	**0.00441**	**0.69429**

Glycerolipids	*Triacylglycerols*	*TG(50:1)*	-13.6	-8.1	49.4	**0.01746**	**0.98541**
Glycerolipids	*Triacylglycerols*	*TG(50:2)*	-12.8	-6.5	48.8	**0.03412**	**0.99932**
Glycerolipids	*Triacylglycerols*	*TG(50:3)*	-23.5	-14	47.3	**0.00256**	**0.52027**
Glycerolipids	*Triacylglycerols*	*TG(50:4)*	-19.2	-14.3	49.6	**0.00316**	**0.58479**
Glycerolipids	*Triacylglycerols*	*TG(50:5)*	-27.2	-27.6	32	**0.00002**	**0.00736**
Glycerolipids	*Triacylglycerols*	*TG(52:1)*	-19.4	2.7	79.3	**0.02527**	**0.99676**
Glycerolipids	*Triacylglycerols*	*TG(52:6)*	-20	-23.8	32.4	**0.00106**	**0.27546**
Glycerolipids	*Triacylglycerols*	*TG(54:5)*	-25.3	7.2	104.8	**0.03092**	**0.9989**
Glycerolipids	*Triacylglycerols*	*TG(54:6)*	-23.1	-22.3	38.1	**0.00001**	**0.00527**
Glycerolipids	*Triacylglycerols*	*TG(56:1)*	-37.6	-23.7	49.5	**0.00007**	**0.02558**
Glycerolipids	*Triacylglycerols*	*TG(56:2)*	-19.9	-19.8	45.8	**0.00023**	**0.07419**
Glycerolipids	*Triacylglycerols*	*TG(56:6)*	-20.1	-16.8	36.2	**0.00061**	**0.17428**
Glycerolipids	*Triacylglycerols*	*TG(56:7)*	-42.1	-26	52	**0.00016**	**0.0531**
Glycerolipids	*Triacylglycerols*	*TG(56:8)*	-41.6	-27.3	54.2	**0.00026**	**0.0802**
Glycerolipids	*Triacylglycerols*	*TG(58:1)*	-30.3	-21.7	42.4	**0.00028**	**0.08686**
Glycerolipids	*Triacylglycerols*	*TG(58:2)*	-40	-30.7	46.9	**0.00001**	**0.00331**
Glycerolipids	*Triacylglycerols*	*TG(58:3)*	-35.6	-27.8	45.7	**0.00005**	**0.01712**
Glycerolipids	*Triacylglycerols*	*TG(58:4)*	-33.5	-24.7	47.3	**0.0001**	**0.03401**
Glycerolipids	*Triacylglycerols*	*TG(58:5)*	-23	-17.7	43.1	**0.00071**	**0.19899**
Glycerolipids	*Triacylglycerols*	*TG(58:8)*	-31.9	-21.6	44.9	**0.0002**	**0.06327**
Glycerolipids	*Triacylglycerols*	*TG(58:9)*	-32.1	-27	40.7	**0.00004**	**0.01549**
Glycerolipids	*Triacylglycerols*	*TG(58:10)*	-33.6	-21.9	43	**0.00091**	**0.24225**
Glycerolipids	*Triacylglycerols*	*TG(60:1)*	-32.7	-18.9	51.4	**0.00023**	**0.07419**
Glycerolipids	*Triacylglycerols*	*TG(60:2)*	-31.1	-28.3	38.6	**0.00001**	**0.0047**
Glycerolipids	*Triacylglycerols*	*TG(60:3)*	-26	-23.7	38.3	**0.00001**	**0.00295**
Sterols	*Cholesteryl Ester*	*ChoE(18:3)*	-27.7	-12.7	35.9	**0.00316**	**0.58479**
Sterols	*Cholesteryl Ester*	*ChoE(20:3)*	-27.1	-17.9	26.8	**0.00008**	**0.02803**
Sterols	*Cholesteryl Ester*	*ChoE(20:4)*	-15.3	-11.3	30.1	**0.0078**	**0.86621**
Sterols	*Cholesteryl Ester*	*ChoE(20:5)*	-33.2	-19.3	47	**0.00193**	**0.42765**
Sterols	*Cholesteryl Ester*	*ChoE(22:5)*	-21.1	-5.1	36	**0.03092**	**0.9989**
Bile acids	*Glycine-conjugated bile acids*	*Glycochenodeoxycholic acid*	48.9	64.7	123.5	**0.04333**	**0.99988**
Bile acids	*Glycine-conjugated bile acids*	*Glycodeoxycholic acid*	82.1	117.5	192.3	**0.00536**	**0.75534**
Bile acids	*Taurine-conjugated bile acids*	*Taurocholic acid*	50.1	122.8	196.8	**0.00362**	**0.62796**
Bile acids	*Taurine-conjugated bile acids*	*Taurodeoxycholic acid*	59	103.4	159.9	**0.00031**	**0.09424**
Bile acids	*Taurine-conjugated bile acids*	*Taurochenodeoxycholic acid*	73.3	95.6	126.2	**0.00167**	**0.3907**
Bile acids	*Taurine-conjugated bile acids*	*Taurolithocholic acid*	-22.6	-7.7	79.9	**0.00295**	**0.56628**
Sterols	*Steroid sulfates*	*isomer pregn-5-ene-3.20-diol sulfate*	-6.7	-4.1	25.2	**0.02798**	**0.99806**

Glycerophospholipids	*PE*	*PE(36:3)*	-22.4	-10.8	49	**0.02162**	**0.9933**
Glycerophospholipids	*ether-PE*	*PE(18:2e/22:6)*	-21	-19.5	21.9	**0.00004**	**0.01549**
Glycerophospholipids	*P-PE*	*PE(P-16:0/22:6)*	-8.6	-9	30.2	**0.03092**	**0.9989**
Glycerophospholipids	*P-PE*	*PE(P-18:1/20:4)*	-23.7	*-17.3*	*31.9*	**0.00026**	**0.0802**
Glycerophospholipids	*LPE*	*PE(14:0/0:0)*	-25.2	*-23.2*	*28.4*	**0.00021**	**0.06855**
Glycerophospholipids	*LPE*	*PE(16:0/0:0)*	-6.1	*-7.1*	*13.7*	**0.00295**	**0.56628**
Glycerophospholipids	*LPE*	*PE(18:0/0:0)*	-9.1	*-8.5*	*16.4*	**0.00338**	**0.60578**
Glycerophospholipids	*LPE*	*PE(18:2/0:0)*	-12.8	*-4.5*	*26.7*	**0.02659**	**0.99755**
Glycerophospholipids	*LPE*	*PE(18:3/0:0)*	-43.4	*-28.5*	*72.9*	**0**	**0.00017**
Glycerophospholipids	*LPE*	*PE(20:3/0:0)*	-16.6	*-16.5*	*29.6*	**0.0002**	**0.06327**
Glycerophospholipids	*LPE*	*LPE 20:5*	-17.4	*-12.6*	*32.2*	**0.00413**	**0.67432**
Glycerophospholipids	*LPE*	*PE(20:5/0:0)*	-24.6	*-17.8*	*29.4*	**0.00023**	**0.07419**
Glycerophospholipids	*LPE*	*PE(22:4/0:0)*	-22.4	*-15.4*	*34*	**0.00061**	**0.17428**
Glycerophospholipids	*LPE*	*PE(22:5/0:0)*	-21.8	*-20*	*35.8*	**0.00013**	**0.04488**
Glycerophospholipids	*LPE*	*PE(22:5/0:0)*	-25.8	*-19.8*	*29.8*	**0.00001**	**0.00202**
Glycerophospholipids	*LPE*	*PE(22:6/0:0)*	-6.4	*-6.9*	*17.3*	**0.024**	**0.99597**
Glycerophospholipids	*LPE*	*PE(0:0/16:0)*	-11.8	*-11.6*	*20.3*	**0.00047**	**0.14017**
Glycerophospholipids	*LPE*	*PE(0:0/18:0)*	-6.1	*-7.3*	*18.7*	**0.01945**	**0.9897**
Glycerophospholipids	*LPE*	*PE(0:0/20:3)*	-18.6	*-14.2*	*30.9*	**0.00071**	**0.19899**
Glycerophospholipids	*LPE*	*PE(0:0/22:4)*	-19.2	*-8.4*	*39.7*	**0.01323**	**0.96122**
Glycerophospholipids	*LPE*	*PE(0:0/22:6)*	-10.5	*-7.6*	*19.3*	**0.02162**	**0.9933**
Glycerophospholipids	*O-LPE*	*PE(O-16:0/0:0)*	-23.4	*-17.6*	*29.5*	**0.00114**	**0.29234**
Glycerophospholipids	*P-LPE*	*PE(P-16:1/0:0)*	-15.6	*-10.2*	*28.7*	**0.02051**	**0.99167**
Glycerophospholipids	*P-LPE*	*PE(P-18:1/0:0)*	-29.4	*-24.9*	*25.1*	**0.00001**	**0.00331**
Glycerophospholipids	*P-LPE*	*PE(P-18:1/0:0)*	27	*63.1*	*96.9*	**0.02942**	**0.99855**
Glycerophospholipids	*P-LPE*	*PE(P-18:2/0:0)*	-30.8	*-29.7*	*23.6*	**0**	**0.00004**
Glycerophospholipids	*P-LPE*	*PE(P-20:0/0:0)*	-18.2	*-16*	*22.2*	**0.00021**	**0.06855**
Glycerophospholipids	*P-LPE*	*PE(P-20:1/0:0)*	-38.1	*-33.6*	*30.8*	**0**	**0.00051**
Glycerophospholipids	*P-LPE*	*PE(P-20:2/0:0)*	-34.5	*-30.3*	*24*	**0**	**0.00028**
Glycerophospholipids	*PC*	*PC(28:0)*	-78.3	*-72*	*28*	**0**	**0.00001**
Glycerophospholipids	*PC*	*PC(30:0)*	-57.5	*-54.6*	*18.6*	**0**	**0**
Glycerophospholipids	*PC*	*PC(30:1)*	-56.5	*-49.7*	*30.2*	**0**	**0.00003**
Glycerophospholipids	*PC*	*PC(31:0)*	-17.1	*-17*	*22.1*	**0.00007**	**0.02558**
Glycerophospholipids	*PC*	*PC(32:0)*	-13.3	*-11.6*	*21.3*	**0.00167**	**0.3907**
Glycerophospholipids	*PC*	*PC(32:1)*	-24.5	*-20.2*	*29.2*	**0.00013**	**0.04488**
Glycerophospholipids	*PC*	*PC(32:2)*	-48	*-45.6*	*21.2*	**0**	**0**
Glycerophospholipids	*PC*	*PC(33:2)*	-14.5	*-13*	*27.2*	**0.00316**	**0.58479**

Glycerophospholipids	*PC*	*PC(34:0)*	-11.6	*-7.2*	*24.4*	**0.024**	**0.99597**
Glycerophospholipids	*PC*	*PC(34:1)*	-8.5	*-8.2*	*24.4*	**0.03582**	**0.99949**
Glycerophospholipids	*PC*	*PC(34:2)*	-9.8	*-8.4*	*21.3*	**0.01051**	**0.92947**
Glycerophospholipids	*PC*	*PC(34:3)*	-26.8	*-7.6*	*68*	**0.00503**	**0.73555**
Glycerophospholipids	*PC*	*PC(34:4)*	-58.8	*-53.7*	*18.4*	**0**	**0**
Glycerophospholipids	*PC*	*PC(35:1)*	17.8	*29.2*	*45.7*	**0.00098**	**0.25849**
Glycerophospholipids	*PC*	*PC(35:2)*	20.1	*28*	*37.9*	**0.00051**	**0.15061**
Glycerophospholipids	*PC*	*PC(35:4)*	-22.6	*-19.5*	*23.9*	**0.00002**	**0.0059**
Glycerophospholipids	*PC*	*PC(36:2)*	-12.8	*-8.3*	*23.9*	**0.01843**	**0.98737**
Glycerophospholipids	*PC*	*PC(36:3)*	-11.6	*-11.8*	*23.8*	**0.00295**	**0.56628**
Glycerophospholipids	*PC*	*PC(36:4)*	-16.5	*-14*	*20.9*	**0.00114**	**0.29234**
Glycerophospholipids	*PC*	*PC(36:4)*	-16.8	*-6.6*	*43.8*	**0.01746**	**0.98541**
Glycerophospholipids	*PC*	*PC(36:5)*	-24.6	*-20.6*	*38*	**0.00167**	**0.3907**
Glycerophospholipids	*PC*	*PC(36:5)*	-24.7	*-20.5*	*38*	**0.00167**	**0.3907**
Glycerophospholipids	*PC*	*PC(36:6)*	-51.7	*-49.9*	*18.8*	**0**	**0**
Glycerophospholipids	*PC*	*PC(37:2)*	32.9	*48.3*	*66.2*	**0.00007**	**0.02558**
Glycerophospholipids	*PC*	*PC(37:6)*	-15.4	*-12.9*	*25.8*	**0.00413**	**0.67432**
Glycerophospholipids	*PC*	*PC(38:2)*	-17	*-18.8*	*23.7*	**0.00002**	**0.00736**
Glycerophospholipids	*PC*	*PC(38:3)*	-23.6	*-22.3*	*27.6*	**0.00001**	**0.00295**
Glycerophospholipids	*PC*	*PC(38:4)*	-13.3	*-8.7*	*20.8*	**0.01323**	**0.96122**
Glycerophospholipids	*PC*	*PC(38:5)*	-29.7	*-26.8*	*39.1*	**0.00003**	**0.0113**
Glycerophospholipids	*PC*	*PC(38:6)*	-12.4	*-10.8*	*24.9*	**0.00239**	**0.49689**
Glycerophospholipids	*PC*	*PC(38:6)*	-19.8	*-20.4*	*23.4*	**0.00002**	**0.0059**
Glycerophospholipids	*PC*	*PC(40:1)*	-23.8	*-21.3*	*29.4*	**0.00007**	**0.02319**
Glycerophospholipids	*PC*	*PC(40:4)*	-19.3	*-18.2*	*28.4*	**0.00061**	**0.17428**
Glycerophospholipids	*PC*	*PC(40:5)*	-10.2	*-8.1*	*26.1*	**0.01565**	**0.97765**
Glycerophospholipids	*PC*	*PC(40:5)*	-25.5	*-21.5*	*29.5*	**0.00001**	**0.00331**
Glycerophospholipids	*PC*	*PC(40:6)*	-10.2	*-8.7*	*22.6*	**0.0088**	**0.89496**
Glycerophospholipids	*PC*	*PC(40:7)*	-34.8	*-27.7*	*29.9*	**0.00001**	**0.00295**
Glycerophospholipids	*PC*	*PC(40:8)*	-10.7	*-12*	*22.1*	**0.00295**	**0.56628**
Glycerophospholipids	*ether-PE*	*PC(18:1e/20:3)*	73.7	*129*	*235.5*	**0.00106**	**0.27546**
Glycerophospholipids	*O-PC*	*PC(O-16:0/14:0)*	-31.9	*-31.7*	*20.1*	**0**	**0.00001**
Glycerophospholipids	*O-PC*	*PC(O-16:0/20:4)*	-14.1	*-7.8*	*31.8*	**0.04333**	**0.99988**
Glycerophospholipids	*O-PC*	*PC(O-16:0/22:4)*	-16.8	*-9.3*	*33.9*	**0.04977**	**0.99996**
Glycerophospholipids	*O-PC*	*PC(O-22:0/20:4)*	-10.9	*-8.6*	*24.6*	**0.02051**	**0.99167**
Glycerophospholipids	*P-PC*	*PC(P-16:0/14:0)*	-27.9	*-27.4*	*19.4*	**0**	**0.00004**
Glycerophospholipids	*P-PC*	*PC(P-16:0/18:2)*	36.9	*43.8*	*51.3*	**0.00015**	**0.0487**

Glycerophospholipids	*P-PC*	*PC(P-16:0/20:4)*	29.8	*33*	*47.7*	**0.01114**	**0.93854**
Glycerophospholipids	*P-PC*	*PC(P-16:0/22:6)*	29.4	*40.9*	*48.4*	**0.00021**	**0.06855**
Glycerophospholipids	*P-PC*	*PC(P-17:0/20:4)*	59.3	*63.2*	*67.4*	**0.00001**	**0.0047**
Glycerophospholipids	*P-PC*	*PC(P-18:0/20:4)*	34.7	*46.6*	*64.9*	**0.00608**	**0.79419**
Glycerophospholipids	*P-PC*	*PC(P-36:2)*	30.3	*40.5*	*53.5*	**0.00077**	**0.21193**
Glycerophospholipids	*LPC*	*PC(14:0/0:0)*	-45.2	*-42.6*	*16.7*	**0**	**0**
Glycerophospholipids	*LPC*	*PC(15:0/0:0)*	-13.7	*-14*	*16.9*	**0.00002**	**0.00736**
Glycerophospholipids	*LPC*	*PC(16:0/0:0)*	-3.7	*-4.1*	*7.9*	**0.00362**	**0.62796**
Glycerophospholipids	*LPC*	*PC(16:1/0:0)*	-23.6	*-21.8*	*21.2*	**0**	**0.00044**
Glycerophospholipids	*LPC*	*PC(17:1/0:0)*	20.3	*25.5*	*45.9*	**0.00828**	**0.88111**
Glycerophospholipids	*LPC*	*PC(18:0/0:0)*	-5.9	*-7.2*	*12*	**0.00047**	**0.14017**
Glycerophospholipids	*LPC*	*PC(18:3/0:0)*	-43.6	*-38.1*	*34.3*	**0**	**0.00005**
Glycerophospholipids	*LPC*	*PC(20:0/0:0)*	-23.3	*-20.4*	*22.6*	**0.00002**	**0.00816**
Glycerophospholipids	*LPC*	*PC(20:1/0:0)*	-9.3	*-10.5*	*20.8*	**0.01051**	**0.92947**
Glycerophospholipids	*LPC*	*PC(20:1/0:0)*	-30	*-9.4*	*52.2*	**0.0088**	**0.89496**
Glycerophospholipids	*LPC*	*PC(20:2/0:0)*	-18.6	*-18.7*	*21.1*	**0**	**0.00038**
Glycerophospholipids	*LPC*	*PC(20:2/0:0)*	-26.2	*-19.2*	*44.4*	**0.00056**	**0.16211**
Glycerophospholipids	*LPC*	*PC(20:3/0:0)*	-16.3	*-11.5*	*34.9*	**0.00013**	**0.04488**
Glycerophospholipids	*LPC*	*PC(20:3/0:0)*	-34.7	*-30.1*	*29.8*	**0**	**0.00051**
Glycerophospholipids	*LPC*	*PC(20:5/0:0)*	-23.1	*-13*	*40.5*	**0.00167**	**0.3907**
Glycerophospholipids	*LPC*	*PC(22:4/0:0)*	-26.9	*-18.3*	*37.8*	**0.00002**	**0.00736**
Glycerophospholipids	*LPC*	*PC(22:5/0:0)*	-29.6	*-21.6*	*31.8*	**0.00001**	**0.00229**
Glycerophospholipids	*LPC*	*PC(0:0/14:0)*	-46.2	*-44.9*	*19.8*	**0**	**0**
Glycerophospholipids	*LPC*	*PC(0:0/15:0)*	-20	*-22.1*	*17.5*	**0**	**0.00006**
Glycerophospholipids	*LPC*	*PC(0:0/16:0)*	-7.8	*-7.9*	*9.7*	**0.00002**	**0.0059**
Glycerophospholipids	*LPC*	*PC(0:0/17:0)*	11.4	*24.2*	*49.6*	**0.02942**	**0.99855**
Glycerophospholipids	*LPC*	*PC(0:0/18:0)*	-12.1	*-11.9*	*15.6*	**0.00005**	**0.01712**
Glycerophospholipids	*LPC*	*PC(0:0/18:1)*	-3.6	*-3.2*	*19*	**0.04134**	**0.99983**
Glycerophospholipids	*LPC*	*PC(0:0/18:2)*	-6.9	*-2.8*	*19.9*	**0.03092**	**0.9989**
Glycerophospholipids	*LPC*	*PC(0:0/20:0)*	-23	*-21.2*	*22.9*	**0.00001**	**0.00372**
Glycerophospholipids	*LPC*	*PC(0:0/20:1)*	-7	*-7.6*	*23.2*	**0.03412**	**0.99932**
Glycerophospholipids	*LPC*	*PC(0:0/20:2)*	-23	*-22.6*	*28.8*	**0**	**0.00032**
Glycerophospholipids	*LPC*	*PC(0:0/20:3)*	-14.9	*-12.3*	*20.4*	**0.00013**	**0.04488**
Glycerophospholipids	*LPC*	*PC(0:0/20:4)*	-9.8	*-9.8*	*24*	**0.00733**	**0.85028**
Glycerophospholipids	*LPC*	*PC(0:0/20:5)*	-13.7	*-10.8*	*41.5*	**0.00338**	**0.60578**
Glycerophospholipids	*LPC*	*PC(0:0/22:5)*	-12.1	*-7*	*24.9*	**0.01323**	**0.96122**
Glycerophospholipids	*LPC*	*PC(0:0/22:5)*	-24.6	*-19*	*30*	**0.00001**	**0.00295**

Glycerophospholipids	*LPC*	*LPC(22:0)*	-27.7	*-24.3*	*25.3*	**0**	**0.00058**
Glycerophospholipids	*LPC*	*LPC(22:1)*	-23.6	*-16.9*	*45.4*	**0.00179**	**0.40585**
Glycerophospholipids	*O-LPC*	*PC(O-18:1/0:0)*	-11.3	*-4.7*	*19*	**0.04134**	**0.99983**
Glycerophospholipids	*O-LPC*	*PC(O-20:0/0:0)*	-4.1	*-8.5*	*21.3*	**0.02659**	**0.99755**
Glycerophospholipids	*O-LPC*	*PC(O-20:2/0:0)*	-15.8	*-6.1*	*34.9*	**0.03759**	**0.99964**
Glycerophospholipids	*O-LPC*	*PC(O-22:0/0:0)*	-16.6	*-14.3*	*14.8*	**0.00001**	**0.00372**
Glycerophospholipids	*O-LPC*	*PC(O-24:2/0:0)*	-9.8	*-8.1*	*21.8*	**0.0118**	**0.94674**
Glycerophospholipids	*P-LPC*	*PC(P-16:0/0:0)*	13.7	*12.1*	*23.2*	**0.01843**	**0.98737**
Glycerophospholipids	*P-LPC*	*PC(P-18:0/0:0)*	21.6	*21.1*	*42.2*	**0.0454**	**0.99991**
Glycerophospholipids	*P-LPC*	*PC(P-18:1/0:0)*	109.7	*117.1*	*118*	**0.00015**	**0.0487**
Glycerophospholipids	*P-LPC*	*PC(P-20:1/0:0)*	-20.2	*-10.4*	*38.4*	**0.024**	**0.99597**
Glycerophospholipids	*P-LPC*	*PC(P-20:2/0:0)*	-17	*-9.9*	*48.1*	**0.0118**	**0.94674**
Glycerophospholipids	*PI*	*PI(36:4)*	-17	*-17.1*	*28.3*	**0.00071**	**0.19899**
Glycerophospholipids	*LPI*	*LPI(16:0)*	-30.7	*-24.2*	*32.5*	**0**	**0.0009**
Glycerophospholipids	*LPI*	*LPI(16:0)*	-31.6	*-21.7*	*39.4*	**0.00001**	**0.00527**
Glycerophospholipids	*LPI*	*LPI(18:0)*	-12.6	*-6*	*41.1*	**0.03249**	**0.99909**
Glycerophospholipids	*LPI*	*LPI(18:0)*	-12.4	*-5.6*	*32*	**0.02659**	**0.99755**
Glycerophospholipids	*LPI*	*LPI(18:2)*	-9.1	*-4.5*	*35.2*	**0.03249**	**0.99909**
Glycerophospholipids	*LPI*	*LPI(20:3)*	-11	*-7.2*	*40.2*	**0.00413**	**0.67432**
Glycerophospholipids	*LPI*	*LPI(22:6)*	-16.4	*-10*	*34.4*	**0.00571**	**0.77449**
Sphingolipids	*Ceramides*	*Cer(d18:1/20:0)*	-16.4	*-9.4*	*37.2*	**0.00223**	**0.47381**
Sphingolipids	*Ceramides*	*Cer(d18:1/22:0)*	-27.2	*-21.8*	*22.2*	**0.00001**	**0.00372**
Sphingolipids	*Ceramides*	*Cer(40:2)*	-9.8	*-6.9*	*21.1*	**0.00503**	**0.73555**
Sphingolipids	*Ceramides*	*Cer(d18:1/23:0)*	-23.1	*-16.3*	*26.4*	**0.00033**	**0.10214**
Sphingolipids	*Ceramides*	*Cer(d18:1/24:0)*	-32.1	*-24.5*	*22.7*	**0**	**0.00078**
Sphingolipids	*Ceramides*	*Cer(d18:1/24:1)+Cer(d18:2/24:0)*	-12	*-10.1*	*25.5*	**0.00275**	**0.54385**
Sphingolipids	*Sphingomyelin*	*SM(31:1)*	-28.7	*-29.8*	*15.6*	**0**	**0.00002**
Sphingolipids	*Sphingomyelin*	*SM(38:0)*	-15.9	*-13.5*	*27.7*	**0.00133**	**0.32882**
Sphingolipids	*Sphingomyelin*	*SM(42:1)*	-21.3	*-16*	*19.5*	**0.00003**	**0.0113**
Sphingolipids	*Sphingomyelin*	*SM(32:1)*	-26.4	*-26.1*	*13.6*	**0**	**0**
Sphingolipids	*Sphingomyelin*	*SM(d18:0/14:0)*	-43.5	*-42.7*	*13.7*	**0**	**0**
Sphingolipids	*Sphingomyelin*	*SM(d18:0/16:0)*	-13.4	-12.1	16.3	**0.00012**	**0.04108**
Sphingolipids	*Sphingomyelin*	*SM(d18:0/22:0)*	-17.9	-12.2	27.4	**0.00155**	**0.36976**
Sphingolipids	*Sphingomyelin*	*SM(d18:1/12:0)*	-46	-44.7	14.3	**0**	**0**
Sphingolipids	*Sphingomyelin*	*SM(d18:1/17:0)*	20	19.3	27.6	**0.00114**	**0.29234**
Sphingolipids	*Sphingomyelin*	*SM(36:2)*	16.7	19	28.3	**0.00167**	**0.3907**
Sphingolipids	*Sphingomyelin*	*SM(38:1)*	-14.6	-14.1	16.9	**0.00004**	**0.01254**

Sphingolipids	*Sphingomyelin*	*SM(39:1)*	-15.9	-13.1	19.6	**0.00036**	**0.11058**
Sphingolipids	*Sphingomyelin*	*SM(d18:1/22:0)*	-16.8	-15.6	18.6	**0.00003**	**0.00908**
Sphingolipids	*Sphingomyelin*	*SM(d18:1/23:0)*	-9.4	-7.4	23.2	**0.01746**	**0.98541**
Sphingolipids	*Sphingomyelin*	*SM(d18:2/14:0)*	-32.5	-30.8	15.1	**0**	**0**
Sphingolipids	*Sphingomyelin*	*SM(d18:2/22:0)*	-13.1	-11.9	19	**0.00061**	**0.17428**
Sphingolipids	*Monohexosylceramides*	*CMH(d18:1/22:0)*	-14.1	-7.5	30	**0.00991**	**0.9187**
Sphingolipids	*Monohexosylceramides*	*CMH(d18:1/24:0)*	-9.3	-5.4	26.7	**0.03759**	**0.99964**

Supplementary Table S10. Description of all lipid species that passed nominal p-value in the Lipidomic analysis for the Gouda cheese test diet. Data are represented as median and mean FC (%) ± SD of the percentage of change (post versus pre-intervention values).

**Class**	**Subclass A**	**Individual notation**	**Median FC (%)**	**Mean FC (%)**	**SD**	**Nominal p value**	**FDR-correction p value**
Oxidized fatty acids	*Hydroxy-octadecadienoic acids*	*x-HODE*	20.4	89.3	247	**0.00414**	**0.77629**
Oxidized fatty acids	*Dihydroxy-octadecadenoic acids*	*12.13-DiHOME*	-27.2	1.8	89.4	**0.03936**	**1**
Glycerolipids	*Triacylglycerols*	*TG(52:0)*	-15.9	4.2	129.7	**0.01243**	**0.98561**
Glycerolipids	*Triacylglycerols*	*TG(54:1)*	-32.9	-10.6	104.1	**0.00806**	**0.94067**
Glycerolipids	*Triacylglycerols*	*TG(54:2)*	-22.8	-9.5	69.3	**0.01977**	**0.9986**
Glycerolipids	*Triacylglycerols*	*TG(56:1)*	-17.7	-8.6	96.5	**0.00806**	**0.94067**
Glycerolipids	*Triacylglycerols*	*TG(56:2)*	-26.4	-11	90.9	**0.0025**	**0.60166**
Glycerolipids	*Triacylglycerols*	*TG(58:1)*	-13.3	-7.2	79.8	**0.03743**	**0.99999**
Glycerolipids	*Triacylglycerols*	*TG(58:2)*	-30.7	-13.7	84.7	**0.0025**	**0.60166**
Glycerolipids	*Triacylglycerols*	*TG(58:3)*	-29.6	1.1	103.6	**0.01668**	**0.99618**
Glycerolipids	*Triacylglycerols*	*TG(58:5)*	-26.7	-9.5	63.3	**0.01977**	**0.9986**
Glycerolipids	*Triacylglycerols*	*TG(60:1)*	-31.8	-6.3	109.3	**0.00544**	**0.85666**
Glycerolipids	*Triacylglycerols*	*TG(60:2)*	-27.5	-8.2	90.4	**0.01485**	**0.99325**
Glycerolipids	*Triacylglycerols*	*TG(60:3)*	-26.3	-4.4	102.8	**0.00859**	**0.94945**
Sterols	*Cholesteryl Ester*	*ChoE(18:1)*	-11.9	-12.2	15.9	**0.00009**	**0.03387**
Sterols	*Cholesteryl Ester*	*ChoE(18:2)*	-8.4	-2.2	24.2	**0.04563**	**1**
Sterols	*Cholesteryl Ester*	*ChoE(18:3)*	-15.7	-9.6	34	**0.0027**	**0.62675**
Sterols	*Cholesteryl Ester*	*ChoE(20:3)*	-20.9	-18.2	20.8	**0.00008**	**0.03065**
Sterols	*Cholesteryl Ester*	*ChoE(20:4)*	-18.8	-13.3	31.2	**0.00859**	**0.94945**
Sterols	*Cholesteryl Ester*	*ChoE(20:5)*	-29.1	-11.4	63.1	**0.01036**	**0.97159**
Sterols	*Cholesteryl Ester*	*ChoE(22:5)*	-9.6	-10.5	31.6	**0.02744**	**0.99987**

Bile acids	*Secondary bile acids*	*Deoxycholic acid*	24.5	78.7	155.2	**0.00475**	**0.81998**
Bile acids	*Glycine-conjugated bile acids*	*Glycochenodeoxycholic acid*	20.7	37.8	69.4	**0.03211**	**0.99997**
Bile acids	*Glycine-conjugated bile acids*	*Glycocholic acid*	52.1	102.8	153.9	**0.00386**	**0.75435**
Bile acids	*Glycine-conjugated bile acids*	*Glycodeoxycholic acid*	26.9	70.4	131.3	**0.03936**	**1**
Bile acids	*Taurine-conjugated bile acids*	*Taurodeoxycholic acid*	39.5	72	148.6	**0.03743**	**0.99999**
Bile acids	*Taurine-conjugated bile acids*	*Taurolithocholic acid*	-45.6	-5.4	141.6	**0.00059**	**0.20154**
Sterols	*Steroid sulfates*	*isomer androsterone sulfate*	15	34.4	79.2	**0.02091**	**0.99898**
Glycerophospholipids	*ether-PE*	*PE(18:1e/22:6)*	-47.2	-37.2	30	**0**	**0.00107**
Glycerophospholipids	*ether-PE*	*PE(18:2e/22:6)*	-32.2	-20.5	40.1	**0.00008**	**0.03065**
Glycerophospholipids	*P-PE*	*PE(P-16:0/18:2)*	-42.9	-26.1	61.6	**0.00004**	**0.01609**
Glycerophospholipids	*P-PE*	*PE(P-16:0/20:4)*	-38.9	-23.7	48.2	**0.00035**	**0.12474**
Glycerophospholipids	*P-PE*	*PE(P-18:0/18:1)*	-47	-38	30.9	**0.00001**	**0.00221**
Glycerophospholipids	*P-PE*	*PE(P-16:0/22:6)*	-28.2	-14.3	42.1	**0.00125**	**0.37497**
Glycerophospholipids	*P-PE*	*PE(P-18:0/20:4)*	-47.3	-34.1	43.8	**0.00018**	**0.06724**
Glycerophospholipids	*P-PE*	*PE(P-18:1/20:4)*	-44.2	-29.7	44.5	**0.00009**	**0.03387**
Glycerophospholipids	*P-PE*	*PE(P-18:0/22:5) + PE(P-20:1/20:4)*	-32.9	-17.3	54.1	**0.00147**	**0.42105**
Glycerophospholipids	*LPE*	*PE(16:0/0:0)*	9.8	12.9	26.1	**0.03211**	**0.99997**
Glycerophospholipids	*LPE*	*PE(18:1/0:0)*	76.3	87.9	70.6	**0**	**0.00001**
Glycerophospholipids	*LPE*	*PE(18:3/0:0)*	37.5	44.4	73.6	**0.0221**	**0.9993**
Glycerophospholipids	*LPE*	*PE(18:3/0:0)*	-20	-4.7	123.2	**0.00038**	**0.13521**
Glycerophospholipids	*LPE*	*LPE(20:5)*	-24.4	-7.2	50.2	**0.014**	**0.99125**
Glycerophospholipids	*O-LPE*	*PE(O-16:0/0:0)*	-22	-12.6	34.6	**0.00475**	**0.81998**
Glycerophospholipids	*P-LPE*	*PE(P-18:0/0:0)*	-10.5	-10.8	28.3	**0.00582**	**0.874**
Glycerophospholipids	*P-LPE*	*PE(P-18:1/0:0)*	-26.9	-10.3	45.7	**0.00664**	**0.90416**
Glycerophospholipids	*PC*	*PC(31:0)*	17.4	28.3	51.6	**0.00475**	**0.81998**
Glycerophospholipids	*PC*	*PC(33:2)*	17.2	30.9	76.4	**0.00859**	**0.94945**
Glycerophospholipids	*PC*	*PC(34:0)*	-12.5	-3.8	43	**0.02334**	**0.99951**
Glycerophospholipids	*PC*	*PC(34:1)*	-16.6	-10.7	29.4	**0.00582**	**0.874**
Glycerophospholipids	*PC*	*PC(34:4)*	-29	-7.1	73.4	**0.00709**	**0.91704**
Glycerophospholipids	*PC*	*PC(36:1)*	-21.4	-20.2	38.2	**0.00004**	**0.01443**
Glycerophospholipids	*PC*	*PC(36:2)*	-10.1	-7.8	44.8	**0.00335**	**0.70522**
Glycerophospholipids	*PC*	*PC(36:3)*	-10.8	-3.7	42.6	**0.04789**	**1**
Glycerophospholipids	*PC*	*PC(36:4)*	-14.2	-10.6	30.9	**0.03936**	**1**
Glycerophospholipids	*PC*	*PC(36:5)*	-14.8	-6.5	56.2	**0.04563**	**1**
Glycerophospholipids	*PC*	*PC(36:6)*	-22.2	2.5	84.3	**0.02334**	**0.99951**
Glycerophospholipids	*PC*	*PC(37:2)*	-21.9	-14.5	31.5	**0.0132**	**0.98879**
Glycerophospholipids	*PC*	*PC(38:2)*	-23.1	-14.3	44.3	**0.00107**	**0.33021**

Glycerophospholipids	*PC*	*PC(38:3)*	-27.1	-21.1	33.5	**0.00024**	**0.08809**
Glycerophospholipids	*PC*	*PC(38:4)*	-16.2	-17.6	25.3	**0.00011**	**0.0414**
Glycerophospholipids	*PC*	*PC(38:5)*	-8.7	-8	29.4	**0.04345**	**1**
Glycerophospholipids	*PC*	*PC(38:5)*	-40	-22.4	48.6	**0.0009**	**0.28944**
Glycerophospholipids	*PC*	*PC(38:6)*	-28	-17.5	47.4	**0.00026**	**0.09623**
Glycerophospholipids	*PC*	*PC(40:1)*	-26.7	-18	46.4	**0.00233**	**0.57646**
Glycerophospholipids	*PC*	*PC(40:4)*	-30.8	-25.9	32.8	**0.00003**	**0.01294**
Glycerophospholipids	*PC*	*PC(40:5)*	-19.7	-15.5	31.6	**0.00125**	**0.37497**
Glycerophospholipids	*PC*	*PC(40:5)*	-31.9	-26.4	30.3	**0.00002**	**0.0063**
Glycerophospholipids	*PC*	*PC(40:6)*	-10.1	-10.2	33.3	**0.0221**	**0.9993**
Glycerophospholipids	*PC*	*PC(40:7)*	-22.2	-20	39.3	**0.00125**	**0.37497**
Glycerophospholipids	*ether-PC*	*PC(18:1e/20:3)*	-42.9	-40.9	23.3	**0**	**0.00001**
Glycerophospholipids		*PC(O-16:0/18:2)*	-38.5	-21.2	58.3	**0.00018**	**0.06724**
Glycerophospholipids		*PC(O-16:0/20:3)*	-38	-28.8	32.3	**0.00002**	**0.00806**
Glycerophospholipids		*PC(O-16:0/20:4)*	-27.7	-21.2	33.6	**0.00003**	**0.01294**
Glycerophospholipids		*PC(O-18:0/18:2)*	-45.4	-37.7	41.8	**0.00001**	**0.00491**
Glycerophospholipids		*PC(O-18:0/20:4)*	-30.6	-21.7	28.1	**0.00003**	**0.01294**
Glycerophospholipids		*PC(O-18:0/22:4)*	-21.8	-16.6	34.1	**0.00018**	**0.06724**
Glycerophospholipids		*PC(O-18:1/18:2)*	-24.2	-15.4	52.4	**0.00022**	**0.08054**
Glycerophospholipids		*PC(O-18:1/22:4)*	-14.1	-11.7	28.4	**0.00622**	**0.88937**
Glycerophospholipids		*PC(O-20:0/20:4)*	-11.8	-7.9	29.5	**0.02893**	**0.99991**
Glycerophospholipids		*PC(O-22:0/20:4)*	-13.3	-10.7	25.6	**0.00806**	**0.94067**
Glycerophospholipids		*PC(O-34:0)*	-15.2	-7.8	36.5	**0.00664**	**0.90416**
Glycerophospholipids		*PC(O-34:1)*	-9.8	-8.1	27.1	**0.00386**	**0.75435**
Glycerophospholipids		*PC(O-38:4)*	-26.3	-19.9	30.3	**0.00013**	**0.05036**
Glycerophospholipids		*PC(O-38:5)*	-16.7	-15.3	32.4	**0.00012**	**0.04569**
Glycerophospholipids		*PC(O-40:5)*	-7.7	-8.1	29.2	**0.01574**	**0.99485**
Glycerophospholipids		*PC(P-16:0/16:0)*	-6.7	-5.8	26.7	**0.03381**	**0.99998**
Glycerophospholipids		*PC(P-16:0/18:1)*	-18.7	-14.2	31.9	**0.00006**	**0.02226**
Glycerophospholipids		*PC(P-16:0/18:2)*	-28.9	-17.8	34.6	**0.00004**	**0.01797**
Glycerophospholipids		*PC(P-16:0/20:4)*	-29	-22.7	27.1	**0.00001**	**0.00491**
Glycerophospholipids		*PC(P-16:0/22:6)*	-23	-21.3	27.8	**0.00004**	**0.01443**
Glycerophospholipids		*PC(P-17:0/20:4)*	-23.8	-15.8	42.3	**0.00172**	**0.47123**
Glycerophospholipids		*PC(P-18:0/20:4)*	10.2	17.9	31.3	**0.00915**	**0.95724**
Glycerophospholipids		*PC(P-18:0/20:4)*	-34	-29.6	24.7	**0**	**0.00079**
Glycerophospholipids		*PC(P-36:2)*	-30.3	-21.6	32.6	**0.00001**	**0.0038**
Glycerophospholipids	*LPC*	*PC(15:0/0:0)*	19	22.1	25	**0.00005**	**0.02004**
Glycerophospholipids	*LPC*	*PC(18:1/0:0)*	55.1	123.1	225.7	**0**	**0.00025**

Glycerophospholipids	*LPC*	*PC(18:3/0:0)*	19.7	29	55.4	**0.0132**	**0.98879**
Glycerophospholipids	*LPC*	*PC(18:3/0:0)*	-16.5	-19.7	38.6	**0.00029**	**0.10498**
Glycerophospholipids	*LPC*	*PC(19:0/0:0)*	20.8	26.7	48.1	**0.00475**	**0.81998**
Glycerophospholipids	*LPC*	*PC(20:1/0:0)*	24.9	57.6	90.3	**0.00216**	**0.55029**
Glycerophospholipids	*LPC*	*PC(20:2/0:0)*	-23.7	-8.4	60.6	**0.01036**	**0.97159**
Glycerophospholipids	*LPC*	*PC(20:3/0:0)*	-16.4	-18.6	30.2	**0.00076**	**0.25207**
Glycerophospholipids	*LPC*	*PC(0:0/15:0)*	31.4	30.6	39	**0.00045**	**0.15937**
Glycerophospholipids	*LPC*	*PC(0:0/17:0)*	12.7	17.6	33	**0.03381**	**0.99998**
Glycerophospholipids	*LPC*	*PC(0:0/20:4)*	-9	-7.6	33.5	**0.03048**	**0.99995**
Glycerophospholipids	*LPC*	*PC(0:0/22:5)*	-23.2	-19	34.2	**0.00054**	**0.18679**
Glycerophospholipids	*P-LPC*	*PC(P-18:0/0:0)*	-10.9	-5.4	42.4	**0.02744**	**0.99987**
Glycerophospholipids	*P-LPC*	*PC(P-18:1/0:0)*	-19	-8.1	57.6	**0.014**	**0.99125**
Sphingolipids	*Ceramides*	*Cer(d18:1/16:0)*	-11.3	-4.5	30	**0.04563**	**1**
Sphingolipids	*Ceramides*	*Cer(d18:1/22:0)*	-18.5	-12.9	36.7	**0.00035**	**0.12474**
Sphingolipids	*Ceramides*	*Cer(d18:1/23:0)*	-4	-5.3	33	**0.04789**	**1**
Sphingolipids	*Ceramides*	*Cer(d18:1/24:0)*	-14.5	-15	31.9	**0.00005**	**0.02004**
Sphingolipids	*Sphingomyelin*	*SM(42:1)*	-14.7	-11	27	**0.00233**	**0.57646**
Sphingolipids	*Sphingomyelin*	*SM(d18:0/22:0)*	-20.2	-5.1	47.3	**0.04136**	**1**
Sphingolipids	*Sphingomyelin*	*SM(d18:1/12:0)*	-12.3	-7.6	24.8	**0.01977**	**0.9986**
Sphingolipids	*Sphingomyelin*	*SM(38:1)*	-10.8	-6.8	26.7	**0.01485**	**0.99325**
Sphingolipids	*Sphingomyelin*	*SM(d18:1/22:0)*	-15.1	-12	29.6	**0.00083**	**0.27032**
Sphingolipids	*Sphingomyelin*	*SM(d18:2/22:0)*	-8.7	-8	29.6	**0.01036**	**0.97159**
Sphingolipids	*Monohexosylceramides*	*CMH(d18:1/22:0)*	-18.2	-13	28.7	**0.0009**	**0.28944**
Sphingolipids	*Monohexosylceramides*	*CMH(d18:1/24:0)*	-7.7	-7.9	25.5	**0.01668**	**0.99618**

Supplementary Table S11. Description of all lipid species that passed nominal p-value in the Lipidomic analysis for the Goutaler-type cheese test diet. Data are represented as median and mean FC (%) ± SD of the percentage of change (post versus pre-intervention values).

Class	Subclass A	Individual notation	Median FC (%)	Mean FC (%)	SD	Nominal p value	FDR-correction p value
Fatty acids	*Saturated fatty acids*	*14:00*	81.4	137.7	229.2	**0.01746**	**0.99212**
Fatty acids	*Saturated fatty acids*	*15:00*	62.5	104	157.4	**0.00536**	**0.80477**
Fatty acids	*Saturated fatty acids*	*16:00*	27.5	39	65.4	**0.02942**	**0.99958**
Fatty acids	*Saturated fatty acids*	*17:00*	57.2	75.6	108.3	**0.00133**	**0.35428**
Fatty acids	*Saturated fatty acids*	*18:00*	12.8	21	31.2	**0.00275**	**0.58348**
Fatty acids	*Monounsaturated fatty acids*	*16:1n-7*	67.5	99.3	144.6	**0.04754**	**0.99999**
Fatty acids	*Monounsaturated fatty acids*	*16:1n-x*	33.3	86.4	189.2	**0.03412**	**0.99987**
Fatty acids	*Monounsaturated fatty acids*	*18:1n-9*	49.1	66	109.4	**0.03582**	**0.99992**
Fatty acids	*Monounsaturated fatty acids*	*18:1n-x*	50.9	83.1	126.7	**0.00013**	**0.04693**
Fatty acids	*Monounsaturated fatty acids*	*19:1n-x*	92.5	102.3	138.7	**0.00991**	**0.94263**
Fatty acids	*Monounsaturated fatty acids*	*20:1n-6*	60.1	79.7	122.2	**0.03943**	**0.99996**
Fatty acids	*Polyunsaturated fatty acids*	*18:2n-6*	53	54.7	84.7	**0.01945**	**0.99502**
Fatty acids	*Polyunsaturated fatty acids*	*18:2n-6 trans*	46	80.8	119.1	**0.00207**	**0.48634**
Fatty acids	*Polyunsaturated fatty acids*	*18:2n-x*	69.7	92.1	135.6	**0.03249**	**0.99981**
Fatty acids	*Polyunsaturated fatty acids*	*18:3n-3*	34	60.6	96.6	**0.03582**	**0.99992**
Oxidized fatty acids	*Hydroxy-octadecadienoic acids*	*x-HODE*	35.6	42	74.2	**0.02659**	**0.99914**
Oxidized fatty acids	*Hydroxy-octadecadienoic acids*	*x-HODE*	37.5	54.6	83.4	**0.00155**	**0.39464**
Fatty esters	*Acylcarnitines*	*AC(16:0)*	29	29.7	43.7	**0.00413**	**0.72402**
Glycerolipids	*Triacylglycerols*	*TG(52:6)*	-19.6	-1.7	66.1	**0.03582**	**0.99992**
Glycerolipids	*Triacylglycerols*	*TG(54:1)*	-16.5	-17.1	47.4	**0.00571**	**0.82271**
Glycerolipids	*Triacylglycerols*	*TG(54:2)*	-31.3	-18.2	41.3	**0.00295**	**0.60653**
Glycerolipids	*Triacylglycerols*	*TG(54:3)*	-18.5	-14.8	44.3	**0.00536**	**0.80477**
Glycerolipids	*Triacylglycerols*	*TG(54:6)*	-13.9	-9.9	47.4	**0.01945**	**0.99502**
Glycerolipids	*Triacylglycerols*	*TG(56:1)*	-27.4	-28.2	31.5	**0.00003**	**0.01069**
Glycerolipids	*Triacylglycerols*	*TG(56:2)*	-35.3	-29.3	32	**0.00005**	**0.01798**
Glycerolipids	*Triacylglycerols*	*TG(56:3)*	-16.2	-18.1	48.1	**0.00106**	**0.29665**
Glycerolipids	*Triacylglycerols*	*TG(56:6)*	-11.5	-9.8	42.4	**0.01843**	**0.99389**
Glycerolipids	*Triacylglycerols*	*TG(58:1)*	-24.6	-28.1	30.9	**0.00002**	**0.00766**
Glycerolipids	*Triacylglycerols*	*TG(58:2)*	-41.5	-36.7	33.2	**0**	**0.00106**
Glycerolipids	*Triacylglycerols*	*TG(58:3)*	-35.2	-32.2	32.7	**0.00002**	**0.00686**

Glycerolipids	*Triacylglycerols*	*TG(58:4)*	-21.8	-16.2	42.6	**0.00828**	**0.91261**
Glycerolipids	*Triacylglycerols*	*TG(58:8)*	-19.2	-17.8	50.4	**0.00167**	**0.41657**
Glycerolipids	*Triacylglycerols*	*TG(58:9)*	-15.3	-17.7	40.7	**0.00338**	**0.65453**
Glycerolipids	*Triacylglycerols*	*TG(58:10)*	-12.9	-13.3	42	**0.04134**	**0.99997**
Glycerolipids	*Triacylglycerols*	*TG(60:1)*	-26	-21.5	34.9	**0.00071**	**0.21427**
Glycerolipids	*Triacylglycerols*	*TG(60:2)*	-39.8	-33.7	32.7	**0**	**0.0006**
Glycerolipids	*Triacylglycerols*	*TG(60:3)*	-37.4	-31.1	29.8	**0.00002**	**0.00686**
Sterols	*Cholesteryl Ester*	*ChoE(18:1)*	-18.2	-15.7	15.6	**0**	**0.00052**
Sterols	*Cholesteryl Ester*	*ChoE(18:3)*	-26.7	-9.9	46.9	**0.00571**	**0.82271**
Sterols	*Cholesteryl Ester*	*ChoE(20:3)*	-18.3	-16	27	**0.00016**	**0.05601**
Sterols	*Cholesteryl Ester*	*ChoE(20:4)*	-20.8	-16.8	30.1	**0.00051**	**0.16101**
Sterols	*Cholesteryl Ester*	*ChoE(20:5)*	-41.9	-29.4	36.1	**0.00005**	**0.01798**
Sterols	*Cholesteryl Ester*	*ChoE(22:5)*	-16.7	-13.5	30.8	**0.00413**	**0.72402**
Bile acids	*Taurine-conjugated bile acids*	*Taurodeoxycholic acid*	54.1	74.4	107.4	**0.0454**	**0.99999**
Bile acids	*Taurine-conjugated bile acids*	*Taurolithocholic acid*	-38.1	-17.1	76	**0.00338**	**0.65453**
Glycerophospholipids	*PE*	*PE(38:6)*	11.8	15.7	38.4	**0.04977**	**1**
Glycerophospholipids	*ether-PE*	*PE(18:1e/22:6)*	-42.4	-39.5	21.1	**0**	**0**
Glycerophospholipids	*ether-PE*	*PE(18:2e/22:6)*	-30.8	-30.3	17.3	**0**	**0.00001**
Glycerophospholipids	*P-PE-*	*PE(P-16:0/18:2)*	-26.5	-28.9	25.3	**0**	**0.0001**
Glycerophospholipids	*P-PE*	*PE(P-16:0/20:4)*	-29.1	-23.8	32.7	**0.00026**	**0.08561**
Glycerophospholipids	*P-PE*	*PE(P-18:0/18:1)*	-39.8	-37.1	25.6	**0**	**0.00009**
Glycerophospholipids	*P-PE*	*PE(P-16:0/22:6)*	-23.4	-22.9	22.1	**0**	**0.0002**
Glycerophospholipids	*P-PE*	*PE(P-18:0/20:4)*	-39	-36.4	31.3	**0.00001**	**0.00266**
Glycerophospholipids	*1P-PE*	*PE(P-18:1/20:4)*	-36.3	-32.7	27.9	**0.00001**	**0.00206**
Glycerophospholipids	*P-PE*	*PE(P-18:0/22:5) + PE(P-20:1/20:4)*	-32.9	-27.8	31.7	**0.00006**	**0.02189**
Glycerophospholipids	*LPE*	*PE(18:1/0:0)*	-13.1	-10.2	22.6	**0.00338**	**0.65453**
Glycerophospholipids	*LPE*	*PE(18:1/0:0)*	55	83.3	100	**0.00001**	**0.00266**
Glycerophospholipids	*LPE*	*PE(18:3/0:0)*	-24.9	-12.7	56.5	**0.01843**	**0.99389**
Glycerophospholipids	*LPE*	*LPE(20:5)*	-22.8	-23.9	30.2	**0.00005**	**0.01985**
Glycerophospholipids	*LPE*	*PE(20:5/0:0)*	-22.4	-13.5	37.1	**0.00503**	**0.7849**
Glycerophospholipids	*LPE*	*PE(0:0/18:1)*	-13.7	-16.5	26.2	**0.00061**	**0.18571**
Glycerophospholipids	*O-LPE*	*PE(O-16:0/0:0)*	-26.9	-19.7	35.3	**0.00179**	**0.43902**
Glycerophospholipids	*P-LPE*	*PE(P-16:1/0:0)*	-16.9	-13.3	35.2	**0.01843**	**0.99389**
Glycerophospholipids	*P-LPE*	*PE(P-18:0/0:0)*	-16.2	-19.2	28.5	**0.00091**	**0.26258**
Glycerophospholipids	*P-LPE*	*PE(P-18:1/0:0)*	-28.6	-24.9	37.3	**0.00026**	**0.08561**
Glycerophospholipids	*P-LPE*	*PE(P-18:2/0:0)*	-18.8	-14.3	50.2	**0.01843**	**0.99389**

Glycerophospholipids	*P-LPE*	*PE(P-20:1/0:0)*	-29.8	-16.7	49.7	**0.00934**	**0.93358**
Glycerophospholipids	*PC*	*PC(31:0)*	19.5	20.2	39.7	**0.00733**	**0.88764**
Glycerophospholipids	*PC*	*PC(34:0)*	-14.7	-12.1	24.7	**0.0078**	**0.90063**
Glycerophospholipids	*PC*	*PC(34:1)*	-15.8	-10.6	26.8	**0.00441**	**0.74498**
Glycerophospholipids	*PC*	*PC(34:4)*	-25.1	-11.4	50.6	**0.00934**	**0.93358**
Glycerophospholipids	*PC*	*PC(35:1)*	-18	-11.5	30.5	**0.00295**	**0.60653**
Glycerophospholipids	*PC*	*PC(36:1)*	-31.1	-26.9	21.3	**0**	**0.00009**
Glycerophospholipids	*PC*	*PC(36:2)*	-19	-17.5	21	**0.00006**	**0.02189**
Glycerophospholipids	*PC*	*PC(36:3)*	-13.2	-10.6	25.8	**0.0125**	**0.97119**
Glycerophospholipids	*PC*	*PC(36:4)*	-15.2	-12.2	29.7	**0.00441**	**0.74498**
Glycerophospholipids	*PC*	*PC(36:4)*	-14.5	-13.9	27.5	**0.01565**	**0.98733**
Glycerophospholipids	*PC*	*PC(36:5)*	-28	-22.3	35.2	**0.00023**	**0.07916**
Glycerophospholipids	*PC*	*PC(36:5)*	-28	-22.4	35.1	**0.00023**	**0.07916**
Glycerophospholipids	*PC*	*PC(36:6)*	-30.4	-15	42.4	**0.014**	**0.98069**
Glycerophospholipids	*PC*	*PC(37:2)*	-17.1	-16.6	32.1	**0.00133**	**0.35428**
Glycerophospholipids	*PC*	*PC(37:5)*	-28.8	-16.1	49.1	**0.00124**	**0.33413**
Glycerophospholipids	*PC*	*PC(38:2)*	-23.5	-25.1	19.7	**0**	**0.00002**
Glycerophospholipids	*PC*	*PC(38:3)*	-27.1	-22.9	32.3	**0.00008**	**0.0293**
Glycerophospholipids	*PC*	*PC(38:4)*	-21.9	-23.3	21.3	**0**	**0.0002**
Glycerophospholipids	*PC*	*PC(38:5)*	-20.9	-15.1	26.3	**0.00021**	**0.07272**
Glycerophospholipids	*PC*	*PC(38:5)*	-43.4	-38.2	28.8	**0**	**0.00009**
Glycerophospholipids	*PC*	*PC(38:6)*	-31.9	-32	20	**0**	**0.00001**
Glycerophospholipids	*PC*	*PC(40:0)*	-9.4	-10.1	17.1	**0.00144**	**0.37419**
Glycerophospholipids	*PC*	*PC(40:1)*	-43	-38	36.3	**0**	**0.0002**
Glycerophospholipids	*PC*	*PC(40:4)*	-33.8	-29.2	28.7	**0**	**0.0008**
Glycerophospholipids	*PC*	*PC(40:4)*	-22.2	-18.3	39.1	**0.00091**	**0.26258**
Glycerophospholipids	*PC*	*PC(40:5)*	-27.7	-25.8	21	**0**	**0.0002**
Glycerophospholipids	*PC*	*PC(40:5)*	-38.6	-26.4	33.7	**0.00001**	**0.0034**
Glycerophospholipids	*PC*	*PC(40:6)*	-14	-18.4	20.4	**0.00001**	**0.00432**
Glycerophospholipids	*PC*	*PC(40:7)*	-10.9	-12.9	28.5	**0.0088**	**0.92359**
Glycerophospholipids	*PC*	*PC(40:7)*	-31.7	-21.9	40.2	**0.00018**	**0.06117**
Glycerophospholipids	*PC*	*PC(40:8)*	-20.3	-20.9	19.7	**0**	**0.00182**
Glycerophospholipids		*PC(18:1e/20:3)*	-45.8	-43.6	25	**0**	**0**
Glycerophospholipids		*PC(O-16:0/18:2)*	-30	-27	34.5	**0.00002**	**0.00614**
Glycerophospholipids		*PC(O-16:0/20:3)*	-26.6	-21.6	41.7	**0.00009**	**0.03225**
Glycerophospholipids		*PC(O-16:0/20:4)*	-27	-23.1	26	**0.00001**	**0.00206**
Glycerophospholipids		*PC(O-18:0/18:2)*	-43.5	-35	39.2	**0.00002**	**0.00614**

Glycerophospholipids		*PC(O-18:0/20:4)*	-27.7	-24.1	25.7	**0**	**0.00182**
Glycerophospholipids		*PC(O-18:0/22:4)*	-29.5	-19.8	34.3	**0.00005**	**0.01985**
Glycerophospholipids		*PC(O-18:1/18:2)*	-24.1	-21	25.4	**0.00007**	**0.02666**
Glycerophospholipids		*PC(O-18:1/22:4)*	-13.5	-16.6	21.2	**0.00003**	**0.01069**
Glycerophospholipids		*PC(O-20:0/20:4)*	-17.7	-13.7	24.9	**0.00023**	**0.07916**
Glycerophospholipids		*PC(O-22:0/20:4)*	-16	-15	24.1	**0.00031**	**0.1006**
Glycerophospholipids		*PC(O-22:1/20:4)*	-10.3	-10	24.9	**0.0078**	**0.90063**
Glycerophospholipids		*PC(O-24:1/20:4)*	-13	-10.4	25.3	**0.00471**	**0.76419**
Glycerophospholipids		*PC(O-34:0)*	-13	-13.8	26.5	**0.00441**	**0.74498**
Glycerophospholipids		*PC(O-34:1)*	-10.4	-12	19.5	**0.00056**	**0.1728**
Glycerophospholipids		*PC(O-38:4)*	-18.1	-16.7	29.2	**0.00013**	**0.04693**
Glycerophospholipids		*PC(O-38:5)*	-19.1	-19.7	22.1	**0**	**0.00122**
Glycerophospholipids		*PC(O-40:5)*	-15	-15.5	22.2	**0.00009**	**0.03225**
Glycerophospholipids		*PC(O-42:6)*	-12.2	-11.5	20.9	**0.00098**	**0.27858**
Glycerophospholipids		*PC(P-16:0/16:0)*	-6.1	-10.6	22.2	**0.00648**	**0.85772**
Glycerophospholipids		*PC(P-16:0/18:1)*	-18.7	-18.5	19.5	**0.00001**	**0.003**
Glycerophospholipids		*PC(P-16:0/18:2)*	-20.6	-19.8	23.5	**0.00004**	**0.01459**
Glycerophospholipids		*PC(P-16:0/20:4)*	-26.6	-26.1	21.8	**0**	**0.00009**
Glycerophospholipids		*PC(P-16:0/22:6)*	-24.1	-26.6	17.2	**0**	**0**
Glycerophospholipids		*PC(P-17:0/20:4)*	-32.2	-26.7	36	**0.00003**	**0.01069**
Glycerophospholipids		*PC(P-18:0/20:4)*	-36.9	-32.7	21	**0**	**0**
Glycerophospholipids		*PC(P-36:2)*	-25.4	-23.2	21.7	**0**	**0.00122**
Glycerophospholipids	*LPC*	*PC(16:1/0:0)*	-17.1	-12.4	28.5	**0.00207**	**0.48634**
Glycerophospholipids	*LPC*	*PC(17:0/0:0)*	-9.1	-7.6	23	**0.02527**	**0.99881**
Glycerophospholipids	*LPC*	*PC(18:0/0:0)*	-9.3	-10.1	15.1	**0.00043**	**0.13855**
Glycerophospholipids	*LPC*	*PC(18:1/0:0)*	44.4	86.2	154.8	**0.00114**	**0.31466**
Glycerophospholipids	*LPC*	*PC(18:3/0:0)*	18.1	32.8	72.2	**0.04977**	**1**
Glycerophospholipids	*LPC*	*PC(18:3/0:0)*	-24.3	-23	30.6	**0.00043**	**0.13855**
Glycerophospholipids	*LPC*	*PC(20:0/0:0)*	-24.5	-20.2	24.5	**0.00003**	**0.01069**
Glycerophospholipids	*LPC*	*PC(20:1/0:0)*	-8.4	-11.3	29.1	**0.02798**	**0.99939**
Glycerophospholipids	*LPC*	*PC(20:2/0:0)*	-18.9	-17.4	39.5	**0.0088**	**0.92359**
Glycerophospholipids	*LPC*	*PC(20:3/0:0)*	-18.8	-19.7	33.1	**0.00012**	**0.04282**
Glycerophospholipids	*LPC*	*PC(20:4/0:0)*	-4.6	-7.9	26.9	**0.04333**	**0.99998**
Glycerophospholipids	*LPC*	*PC(20:5/0:0)*	-29.3	-18.4	39.2	**0.00144**	**0.37419**
Glycerophospholipids	*LPC*	*PC(22:4/0:0)*	-14.5	-11.3	35.3	**0.01114**	**0.95847**
Glycerophospholipids	*LPC*	*PC(22:5/0:0)*	-11.2	-11	35.4	**0.014**	**0.98069**
Glycerophospholipids	*LPC*	*PC(0:0/17:1)*	-11	-11.8	32.3	**0.01323**	**0.97631**

Glycerophospholipids	*LPC*	*PC(0:0/18:0)*	-11.7	-16.5	22.1	**0.00007**	**0.02666**
Glycerophospholipids	*LPC*	*PC(0:0/18:1)*	-12.4	-13.7	21.5	**0.0004**	**0.12829**
Glycerophospholipids	*LPC*	*PC(0:0/20:0)*	-24.9	-23	26.7	**0.00001**	**0.00547**
Glycerophospholipids	*LPC*	*PC(0:0/20:1)*	-12.9	-10.3	35.9	**0.04977**	**1**
Glycerophospholipids	*LPC*	*PC(0:0/20:4)*	-15.2	-13	28.6	**0.00689**	**0.87276**
Glycerophospholipids	*LPC*	*PC(0:0/20:5)*	-21.9	-17.3	40	**0.00239**	**0.53399**
Glycerophospholipids	*LPC*	*PC(0:0/22:5)*	-27.1	-18	33.5	**0.00091**	**0.26258**
Glycerophospholipids	*LPC*	*LPC(22:0)*	-27.6	-27.9	20.9	**0**	**0.00005**
Glycerophospholipids	*LPC*	*LPC(22:1)*	-32.6	-17.3	75.8	**0.00077**	**0.2294**
Glycerophospholipids	*O-LPC*	*PC(O-16:0/0:0)*	-4.8	-7.2	17.9	**0.03582**	**0.99992**
Glycerophospholipids	*O-LPC*	*PC(O-18:0/0:0)*	-16.9	-19.8	22.1	**0.00003**	**0.01069**
Glycerophospholipids	*O-LPC*	*PC(O-18:1/0:0)*	-6.8	-10.2	20.9	**0.00733**	**0.88764**
Glycerophospholipids	*O-LPC*	*PC(O-20:0/0:0)*	-11.3	-9.6	22.6	**0.01051**	**0.95076**
Glycerophospholipids	*O-LPC*	*PC(O-20:1/0:0)*	-11.2	-10.4	28.2	**0.02051**	**0.99605**
Glycerophospholipids	*O-LPC*	*PC(O-22:0/0:0)*	-13.1	-13.6	18.6	**0.00026**	**0.08561**
Glycerophospholipids	*O-LPC*	*PC(O-24:1/0:0)*	-7.3	-6.3	21.5	**0.04333**	**0.99998**
Glycerophospholipids	*P-LPC*	*PC(P-16:0/0:0)*	-6.2	-8.7	16.9	**0.00828**	**0.91261**
Glycerophospholipids	*P-LPC*	*PC(P-18:0/0:0)*	-22.2	-22.7	28.4	**0.00003**	**0.01069**
Glycerophospholipids	*P-LPC*	*PC(P-18:1/0:0)*	-13.8	-13.3	32.9	**0.02279**	**0.99778**
Glycerophospholipids	*P-LPC*	*PC(P-18:1/0:0)*	-24	-24.8	32	**0.00015**	**0.05122**
Glycerophospholipids	*P-LPC*	*PC(P-20:1/0:0)*	-19.9	-13.9	44.3	**0.0118**	**0.96527**
Glycerophospholipids	*PI*	*PI(36:4)*	-13	-5.6	39.6	**0.03759**	**0.99994**
Glycerophospholipids	*PI*	*PI(38:4)*	-9.1	-9.8	26.8	**0.02162**	**0.99702**
Glycerophospholipids	*LPI*	*LPI(18:1)*	-8.8	-11.7	24.8	**0.03759**	**0.99994**
Sphingolipids	*Ceramides*	*Cer(d18:1/20:0)*	-17.2	-11.6	30.2	**0.00275**	**0.58348**
Sphingolipids	*Ceramides*	*Cer(d18:1/22:0)*	-20.6	-19.4	17.7	**0**	**0.00052**
Sphingolipids	*Ceramides*	*Cer(40:2)*	-7.8	-6.3	17.7	**0.03943**	**0.99996**
Sphingolipids	*Ceramides*	*Cer(d18:1/24:0)*	-20.5	-19.1	20.6	**0.00001**	**0.00383**
Sphingolipids	*Sphingomyelin*	*SM(42:1)*	-17.1	-17	18.6	**0.00001**	**0.00432**
Sphingolipids	*Sphingomyelin*	*SM(d18:0/16:0)*	-14.3	-7.6	23.5	**0.01945**	**0.99502**
Sphingolipids	*Sphingomyelin*	*SM(d18:0/22:0)*	-19.3	-12.1	38.5	**0.00648**	**0.85772**
Sphingolipids	*Sphingomyelin*	*SM(d18:1/12:0)*	-15.7	-5.9	34.2	**0.01653**	**0.98996**
Sphingolipids	*Sphingomyelin*	*SM(d18:1/16:0)*	-8	-6.8	20.3	**0.014**	**0.98069**
Sphingolipids	*Sphingomyelin*	*SM(38:1)*	-12.9	-11.5	18.4	**0.00051**	**0.16101**
Sphingolipids	*Sphingomyelin*	*SM(39:1)*	-9.7	-7.5	23.2	**0.00991**	**0.94263**
Sphingolipids	*Sphingomyelin*	*SM(d18:1/22:0)*	-17.9	-18.8	17.6	**0**	**0.00045**
Sphingolipids	*Sphingomyelin*	*SM(d18:1/23:0)*	-9.9	-6.1	26.2	**0.03582**	**0.99992**

Sphingolipids	*Sphingomyelin*	*SM(d18:2/14:0)*	-9.9	-5.8	26.4	**0.024**	**0.99836**
Sphingolipids	*Sphingomyelin*	*SM(d18:2/22:0)*	-12.7	-13.7	16.6	**0.00002**	**0.00766**
Sphingolipids	*Monohexosylceramides*	*CMH(d18:1/22:0)*	-18.7	-15.8	26.5	**0.00106**	**0.29665**
Sphingolipids	*Monohexosylceramides*	*CMH(d18:1/24:0)*	-17.9	-14.4	20.2	**0.0002**	**0.06673**

Supplementary Figure S2. Heatmap representing the results of the lipidomics' analysis for free fatty acids according to the four test diets (Gouda and Goutaler-type cheese. pork. and beef). Data are represented as median ± SD of the percentage of change (post versus pre-intervention values) (adjusted Wilcoxon signed-rank test. Holm-Sidak method).

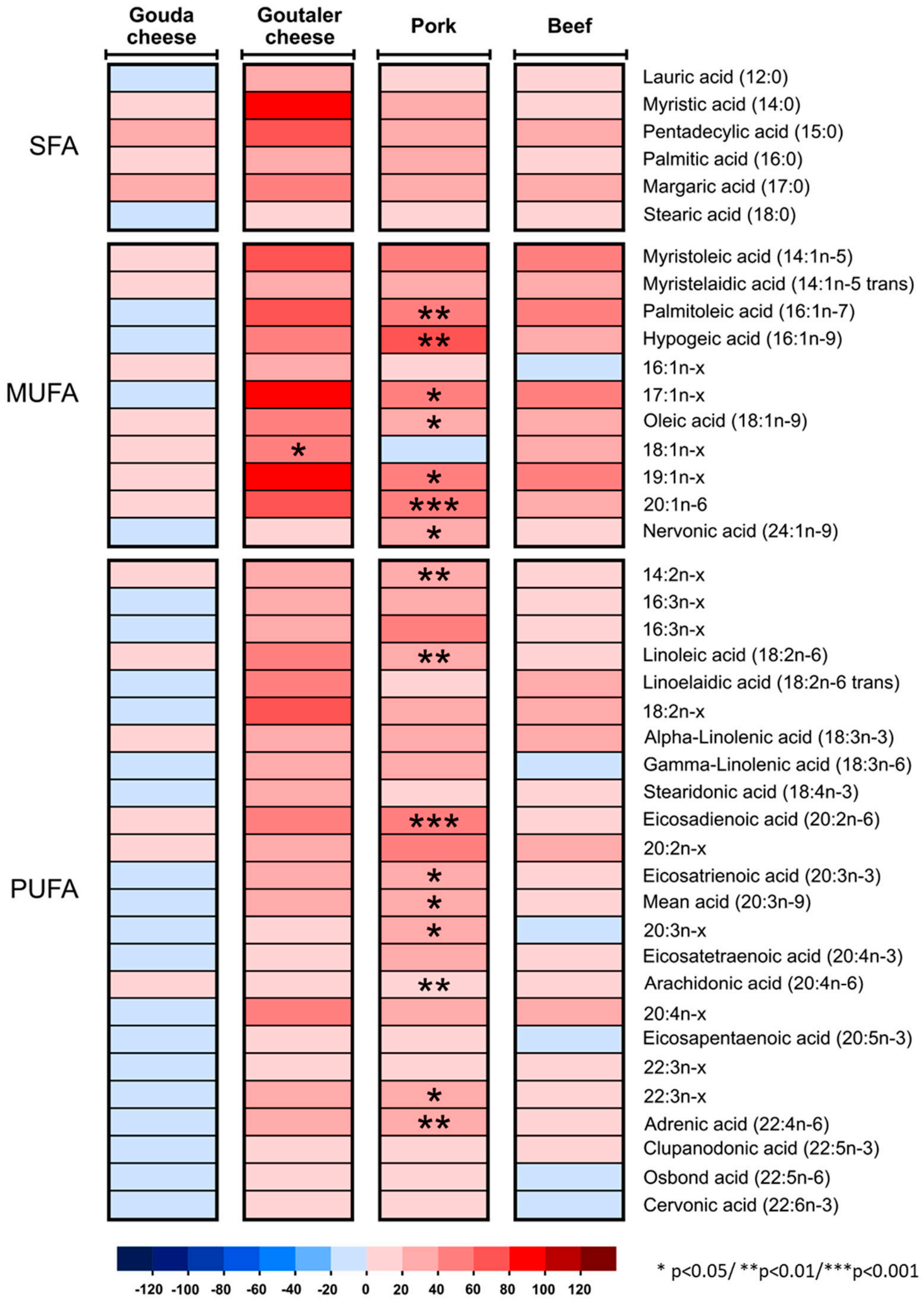

Supplementary Figure S3. Heatmap representing the results (median ± SD of the percentage of change post versus pre-intervention values) of the ceramides from the lipidomic analysis for the four test diets (Gouda-type cheese and Goutaler-type cheese. pork. and beef meat) (A): comparisons between test diets for ceramides' class (B) and for Cer 18:1/24:0 (C) and Cer 18:1/22:0 (D).

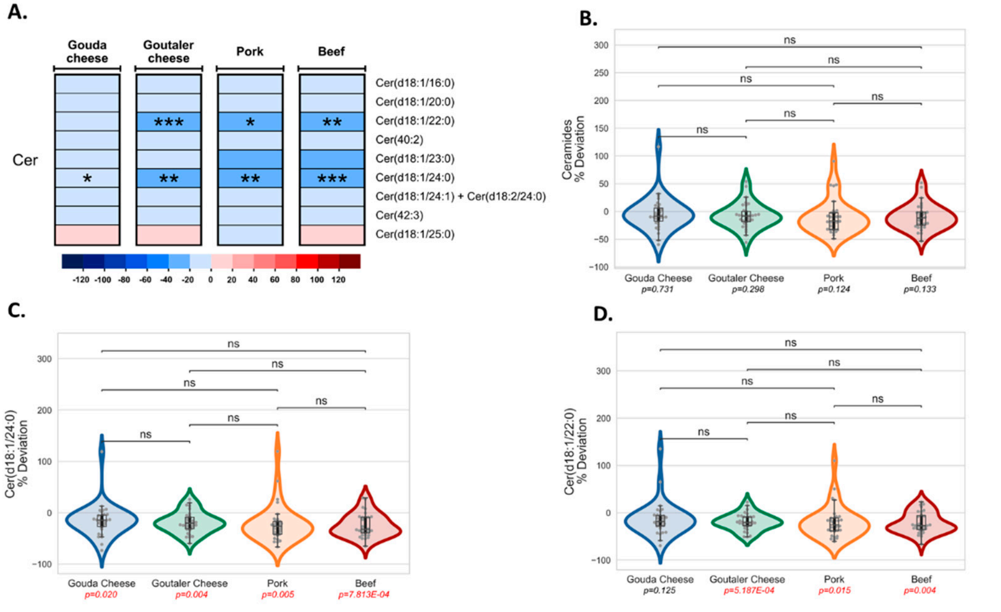

* p<0.05/ **p<0.01/***p<0.001/#p<0.10/ns:non-significant

Supplementary Figure S4. Comparisons between diets for NMR Lipoprotein parameters. cholesterol non-HDL (A). VLDL-c and IDL-c. HDL-c (C). VLDL-TG (D). IDL-TG (E). HDL-TG (F). VLDL-P (G). small VLDL-P (H). large VLDL-P (I). HDL-P (J). small HDL-P (K). LDL-P/HDL-P (L) and total cholesterol in lipoproteins (M).

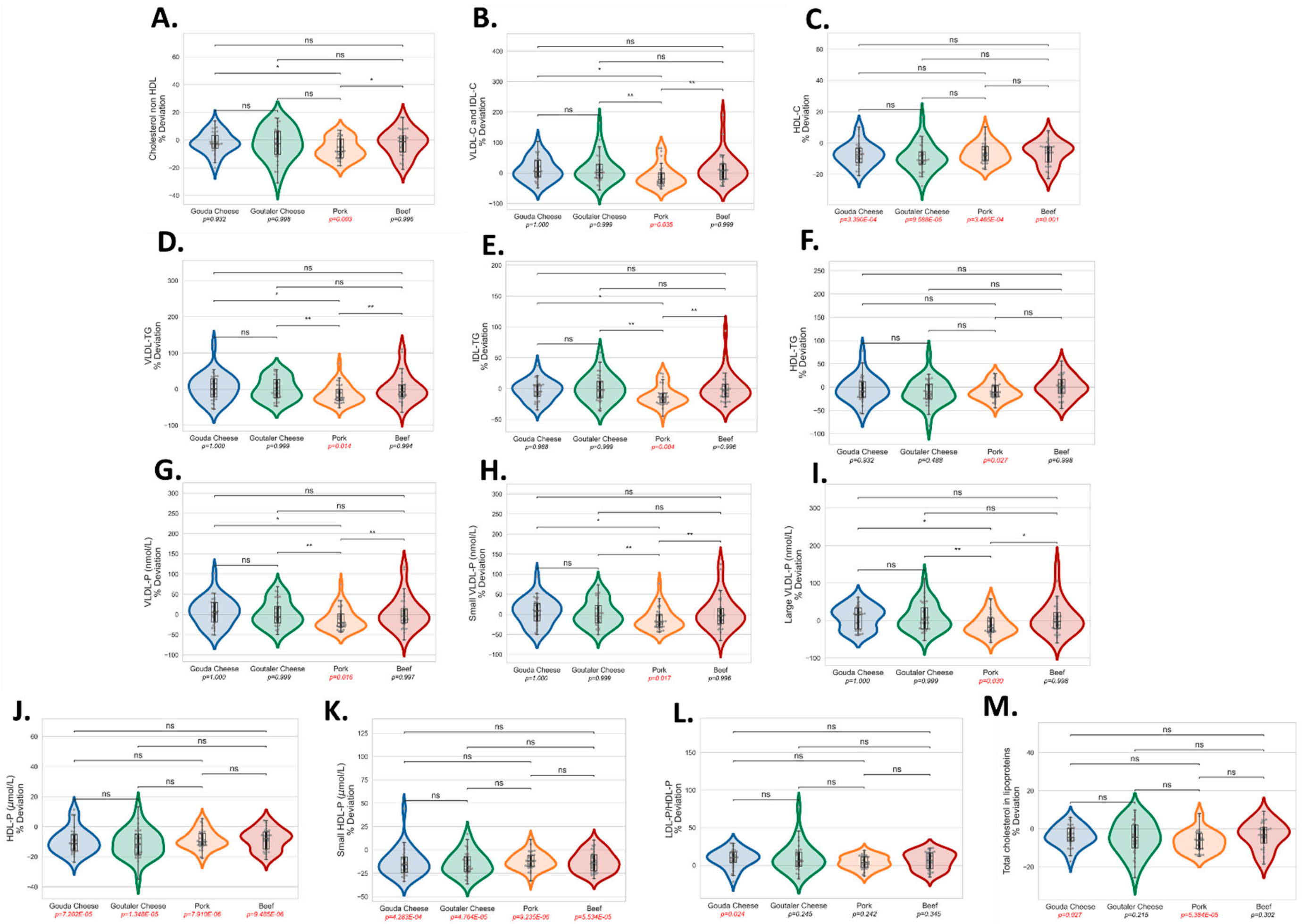

* p<0.05/ **p<0.01/***p<0.001/#p<0.10/ns:non-significant

References

1. Campos-Giménez E, Fontannaz P, Trisconi MJ, Kilinc T, Gimenez C, Andrieux P. Determination of vitamin B12 in food products by liquid chromatography/UV detection with immunoaffinity extraction: single-laboratory validation. J AOAC Int. 2008 Jul-Aug;91(4):786-93. PMID: 18727538.
2. Jørgensen CE, Abrahamsen RK, Rukke EO, Johansen AG, Schüller RB, Skeie SB, Improving the structure and rheology of high protein, low fat yoghurt with undenatured whey proteins. International Dairy Journal, 2015. **47**: p. 6-18.DOI: 10.1016/j.idairyj.2015.02.002.
3. HUBRO-protocol. Protocol for The Oslo Health Study. In Norwegian: Protokoll for Helseundersøkelsen i Oslo (HUBRO) 2000-2001 – del A. Vedlegg 7 - Protokoll-tillegg 10: Rutiner for hjerte-karundersøkelser. Revisjon 2000. Oslo: Statens helseundersøkelser, 2002.
4. Barr J, Caballería J, Martínez-Arranz I, Domínguez-Díez A, Alonso C, Muntané J, et al. Obesity-dependent metabolic signatures associated with nonalcoholic fatty liver disease progression. J Proteome Res. 2012 Apr 6;11(4):2521-32. doi: 10.1021/pr201223p. Epub 2012 Mar 15. PMID: 22364559; PMCID: PMC3321123.
5. Martínez-Arranz I, Mayo R, Pérez-Cormenzana M, Mincholé I, Salazar L, Alonso C, Mato JM. Enhancing metabolomics research through data mining. J Proteomics. 2015 Sep 8;127(Pt B):275-88. doi: 10.1016/j.jprot.2015.01.019. Epub 2015 Feb 7. PMID: 25668325.
6. Mallol R, Amigó N, Rodríguez MA, Heras M, Vinaixa M, Plana N. Liposcale: a novel advanced lipoprotein test based on 2D diffusion-ordered 1H NMR spectroscopy. J Lipid Res. 2015 Mar;56(3):737-746. doi: 10.1194/jlr.D050120. Epub 2015 Jan 7. PMID: 25568061; PMCID: PMC4340320.
7. McKinney, W., Pandas: a python data analysis library.
8. Virtanen P, Gommers R, Oliphant TE, Haberland M, Reddy T, Cournapeau D, et al; SciPy 1.0 Contributors. SciPy 1.0: fundamental algorithms for scientific computing in Python. Nat Methods. 2020 Mar;17(3):261-272. doi: 10.1038/s41592-019-0686-2. Epub 2020 Feb 3. Erratum in: Nat Methods. 2020 Feb 24;: PMID: 32015543; PMCID: PMC7056644.
9. Vallat, R. Pingouin: statistics in Python . J Open Source Softw 2018. **3**, https://doi.org/10.21105/joss.01026.
